# Viral oncogenesis in cancer: from mechanisms to therapeutics

**DOI:** 10.1038/s41392-025-02197-9

**Published:** 2025-05-12

**Authors:** Qing Xiao, Yi Liu, Tingting Li, Chaoyu Wang, Sanxiu He, Liuyue Zhai, Zailin Yang, Xiaomei Zhang, Yongzhong Wu, Yao Liu

**Affiliations:** 1 Chongqing Key Laboratory of Translational Research for Cancer Metastasis and Individualized Treatment, Department of Hematology-Oncology, Chongqing University Cancer Hospital, Chongqing, China; 2 Department of Radiation Oncology, Chongqing University Cancer Hospital, Chongqing, China

**Keywords:** Tumour virus infections, Oncogenes

## Abstract

The year 2024 marks the 60th anniversary of the discovery of the Epstein-Barr virus (EBV), the first virus confirmed to cause human cancer. Viral infections significantly contribute to the global cancer burden, with seven known Group 1 oncogenic viruses, including hepatitis B virus (HBV), human papillomavirus (HPV), EBV, Kaposi sarcoma-associated herpesvirus (KSHV), hepatitis C virus (HCV), human T-cell leukemia virus type 1 (HTLV-1), and human immunodeficiency virus (HIV). These oncogenic viruses induce cellular transformation and cancer development by altering various biological processes within host cells, particularly under immunosuppression or co-carcinogenic exposures. These viruses are primarily associated with hepatocellular carcinoma, gastric cancer, cervical cancer, nasopharyngeal carcinoma, Kaposi sarcoma, lymphoma, and adult T-cell leukemia/lymphoma. Understanding the mechanisms of viral oncogenesis is crucial for identifying and characterizing the early biological processes of virus-related cancers, providing new targets and strategies for treatment or prevention. This review first outlines the global epidemiology of virus-related tumors, milestone events in research, and the process by which oncogenic viruses infect target cells. It then focuses on the molecular mechanisms by which these viruses induce tumors directly or indirectly, including the regulation of oncogenes or tumor suppressor genes, induction of genomic instability, disruption of regular life cycle of cells, immune suppression, chronic inflammation, and inducing angiogenesis. Finally, current therapeutic strategies for virus-related tumors and recent advances in preclinical and clinical research are discussed.

## Introduction

Cancer is a major public health and economic concern of the 21st century. Globally, almost 1.4 million new cancer cases are ascribed to viral infections, accounting for 8% of all cancer cases. While this attributable estimate is large, it is likely to be significantly underestimated, as developing countries are particularly severely affected by virus-related cancers, but often lack cancer registries. The International Agency for Research on Cancer (IARC) has identified seven viruses that are classified as Group 1 carcinogens (Carcinogenic to humans), including human papillomavirus (HPV), hepatitis B virus (HBV), Hepatitis C virus (HCV), Epstein–Barr virus (EBV), Kaposi sarcoma-associated herpesvirus (KSHV), human T cell lymphotropic virus type-1 (HTLV-1), and human immunodeficiency virus (HIV). Each group of viruses can cause various cancers.^1^

Most people will contract at least one known human oncogenic virus during their lifetime.^2^ The majority of viral infections are self-limiting and resolve within a few months without any oncogenic potential. However, infected cells can acquire oncogenesis features, especially when immunosuppressed or exposed to co-oncogenic stimuli.^2,3^ In fact, there is a long latency period between primary infection and the occurrence of cancer. This means that the onset of cancer is not part of the viral replication cycle, but the combination of multiple factors is necessary for the virus to manifest its oncogenic potential in a susceptible host.^4^ Viral oncogenesis is mainly achieved through multifaceted intricate mechanisms, including insertion of viral genes, regulation of host cell genomes and signaling pathways mediated by viral proteins, chronic inflammation resulting from persistent infection, and disorder of tumor suppressor genes and host oncogenes involved in cell growth.^5,6^ The oncogenic mechanisms of viruses are complex and have not been fully elucidated.

Understanding the mechanisms targeting viral oncogenesis has provided vital clues for developing new therapeutic strategies. Current treatment strategies primarily involve antiviral medications, inhibition of viral-encoded proteins to disrupt viral oncogenic mechanisms, or developing vaccines to control viral infections and prevent virus-related cancers.^7,8^ Nevertheless, current treatment outcomes remain challenging. Consequently, it is essential to have a thorough knowledge of the mechanisms by which oncogenic viruses function and cutting-edge treatment approaches. This article aims to comprehensively summarize the characteristics of oncogenic viruses, the mechanisms by which they induce tumors, and the therapeutic strategies for targeting virus-induced oncogenesis. This review will first provide an overview of the worldwide prevalence of oncogenic-virus-related tumors, then review the milestone events in the research history of oncogenic viruses. Subsequently, it will describe in detail the mechanisms of initial infection of target cells by oncogenic viruses and the molecular mechanisms that mediate tumorigenesis during the long-term chronic infection process. Finally, current targeted therapeutic strategies for virus-induced oncogenesis, as well as the progress in pre-clinical and clinical studies, will be introduced. This knowledge is expected to significantly contribute to the future development of basic research and clinical translation in virus-induced tumorigenesis.

## The prevalence of virus-related cancer globally

More than 1.4 million cancer cases are caused by viral infections every year, which brings a significant health burden to the whole world.^9,10^ Data from 2018 indicated that among virus-related cancers, 49% were induced by HPV, 26% were triggered by HBV, 11% were related to HCV, 11% were caused by EBV, and 3% were associated with other viruses (KSHV and HTLV-1).^11^ The age-standardized incidence rates (ASIR) of virus-related cancers show significant regional differences in global distribution (Table 1), and are affected to some extent by gender and national income.

**Table 1:** The age-standardized global incidence and number of cancer cases attributable to viral infections in 2022, by oncoviruses and geographical region

Region	HPV		HBV		HCV		EBV		KSHV		HTLV-1	
	ASIR	NNC	ASIR	NNC	ASIR	NNC	ASIR	NNC	ASIR	NNC	ASIR	NNC
Africa	30.0	137,000	4.6	40,000	2.3	21000	1.1	24,000	2.3	27,000	-	<500
Latin America and the Caribbean	18.5	75,000	2.7	23,000	1.1	9700	0.2	8300	0.4	2800	-	<500
Northern America	14.1	40,000	3.7	26,000	2.6	19000	0.3	5800	0.2	1200	-	<1000
Europe	17.2	99,000	2.8	48,000	1.9	31000	0.3	12,000	0.2	2700	-	<1000
Pacific Ocean	15.4	4400	4.1	2600	1.8	1100	0.4	<500	0.1	<100	-	<100
Asia	16.1	460,000	5.5	330,000	2.5	150000	1.8	120,000	0.1	2500	-	2000
Global	18.5	820,000	3.9	470,000	2.0	230000	1.4	170,000	0.4	36,000	-	4000

Data are ASIR per 100, 000 person-years. The number of cases have been rounded to two significant digits. ASIR, age-standardized incidence rate; NNC, Number of New Cases. “-” represents an incidence too low to calculate. The list of countries for each region or subregion can be found at Cancer today’s data & methods section (https://gco.iarc.fr/today)

In 2018, HBV was responsible for 360,000 new cancer cases (ASIR 4.1 per 100,000 person-years); HCV led to 160,000 new cancer cases (ASIR 1.7 per 100,000 person-years), mainly hepatocellular carcinoma (HCC) (90%) and non-Hodgkin lymphoma (NHL) (10%).^11^ 15%–40% of untreated chronic HBV patients progress to cirrhosis and HCC, with higher male risk.^12^ Owing to the inadequacy of early prevention, screening, and antiviral treatment, less developed regions, such as Asia and Africa, have become the main endemic areas for HBV and HCV.^13,14^ The promotion of HBV vaccination has reduced the prevalence of HBsAg (hepatitis B surface antigen) in children under 5 years old from over 5% to 0.9%.^12,15^ Although no preventive HCV vaccine is available currently, direct-acting antivirals (DAAs) treatment has enabled over 98% of HCV patients to achieve viral clearance.^16^ From 2010–2019, ASIR of HBV-related and HCV-related HCC declined except in Americas, due to vaccination and antiviral therapy success.^17^ However, the number of HCV-related HCC cases and disability-adjusted life years (DALYs) are increasing because of large early infection base.^18,19^

The ASIR of HPV-attributable cancer is five or more per 100,000 person-years in most countries and regions except the Middle East.^11^ HPV is predominantly prevalent in Asia, Africa, and the Americas. The number of infection cases in these regions accounts for 54%, 19%, and 16% of the global total, respectively.^20^ HPV types 16 and 18 account for approximately 72% of all HPV-induced cancer cases, while HPV types 31, 33, 45, 52, and 58 account for an additional 17%.^11,21^ Infection with HPV is responsible for nearly all cases of cervical cancer (CC) and a significant proportion of oropharyngeal (31%), anal cancers (88%).^22^ The ASIR of CC shows a significant negative correlation with income levels, a trend primarily influenced by the accessibility of CC screening.^11,23^ Over the past few decades, the incidence and mortality of CC have steadily declined, particularly in many high-income countries in North America and Europe. However, the incidence of oropharyngeal and anal cancers has been rising in most countries, with a significantly higher burden in more developed countries compared to less developed ones.^20,22^ 70%–90% of HPV-attributable cancer cases can be prevented by widespread high-coverage HPV vaccination.^24,25^ Universal vaccination and early screening are the keys to preventing the majority of HPV-attributable cancer cases.^26^

EBV is a highly prevalent and persistent human viral infection, with about 95% of the global population having asymptomatic EBV infections lifelong.^27,28^ EBV-associated cancers account for around 1.3–1.9% of the global cancer burden, encompassing nasopharyngeal carcinoma (NPC), Burkitt lymphoma (BL), Hodgkin lymphoma (HL), and gastric cancer (GC).^29^ Among the four EBV-attributable cancers, males have a higher proportion than females.^30^ 95% of NPC cases are attributable to EBV, with a predominant occurrence in East Asia, Southeast Asia, and the Middle East.^31^ The estimated number of new EBV-attributable BL cases in 2018 was 6,600, mainly in children aged 5–10 years.^32^ EBV-attributable HL makes up about 40% of global HL cases. Its incidence is positively correlated with a country’s social demographic index (SDI), but the mortality burden is mainly in low and low middle SDI countries, indicating insufficient HL treatment resources in low-income areas.^33^ The EBV prevalence in GC is about 8%, and EBV infection increases the risk of GC by more than 18 times.^34,35^ Given the current lack of effective anti-EBV drugs or vaccines, EBV-attributable cancers pose a challenging aspect of the global disease burden.^36^

The prevalence of HTLV-1 varies from less than one case per 10,000 individuals to over 10%, with the highest prevalence observed in Japan, South America, the Caribbean, Central Australia, and Western, Central, and Southern Africa.^37^ Adult T-cell leukemia/lymphoma (ATLL), a rare tumor induced by HTLV-1, had 3,600 new cases in 2018.^11^ Another rare virus-related cancer is Kaposi sarcoma (KS), which is closely associated with KSHV infection and had 42,000 new cases in 2018.^11^ HIV, a Group 1 carcinogen by IARC, has indirect carcinogenicity, complicating the classification of HIV-related cancers.^38,39^ In general, NHL, HL, and oropharyngeal cancer are more prevalent in men living with HIV, while CC and NHL are more incident in women living with HIV.^40–42^

## The research history and milestone events of viral oncogenesis in cancers

The year 2024 commemorates the 60th anniversary of the human discovery of EBV, the first virus proven to cause cancer in humans. The history of the finding of viruses that cause cancer in humans dates back to the early 1900s. In 1908, when Ellerman and Bang tested whether cancer could be an infectious disease, they found that leukemia could be transmitted from one bird to another by injection.^43^ In addition, they obtained avian leukosis virus (ALV), the first known tumor virus.^44^ For a variety of reasons, their work was not given due attention at the time. In 1910, Rous successfully transplanted sarcoma from a barred Plymouth Rock hen to other chickens.^45^ By 1911, his research had demonstrated that cancer could be transmitted through one virus, which he named Rous sarcoma virus (RSV).^46^ As before, this work was not generally accepted as evidence that a virus could cause cancer. Rous’s contributions to the field were not widely acknowledged until 1966, when he was awarded the Nobel Prize in Physiology or Medicine. In 1933, Shope and Hurst found rabbit papillomavirus (CRPV) in wild cottontail rabbits and those infected rabbits, which exhibited weird protrusions on their heads and necks.^47^ In 1951, Gross discovered that murine leukemia virus (MLV) could cause spontaneous leukemia in C3H mice.^48^ In 1962, Eddy et al. found that simian virus 40 (SV40) was shown to induce tumors in rhesus monkeys.^49^ These studies further strengthened the association between viruses and cancer, thus triggering the first strong interest in tumor virology.

In 1964, Epstein and Barr et al. first found EBV in BL patients.^50^ EBV was later shown to cause mononucleosis and nasopharyngeal carcinoma.^51^ In 1984, Baer et al. sequenced and analyzed the expression of EBV genome.^52^ In 1965, Blumberg identified a novel antigen in the blood of an Aboriginal Australian, which was subsequently designated HBsAg.^53^ This groundbreaking achievement earned him the 1976 Nobel Prize. In 1981, a study in Taiwan found that HBV infection was associated with HCC.^54^ And in the same year, the first human anti-tumor vaccine, the HBV vaccine, was approved. In 1970, Temin and Baltimore proposed independent but related hypotheses for reverse transcriptase. Temin proposed a role for reverse transcriptase in the replication of RSV viruses.^55^ Baltimore independently proposed a similar idea and revealed that the RNA oncolytic virus has an enzyme in its viral particles that synthesizes DNA from RNA templates.^56^ In 1975, together with their mentor Dulbecco, they received a Nobel Prize for discovering transcriptase. Combined with the fact that Rous won the Nobel Prize in 1966 for the discovery of RSV, an event that prompted Peter Duesburg (a retroviralist) to jest, “One sick chicken, two Nobel Prizes.” But that’s not the end of the story.

**Fig. 1:**
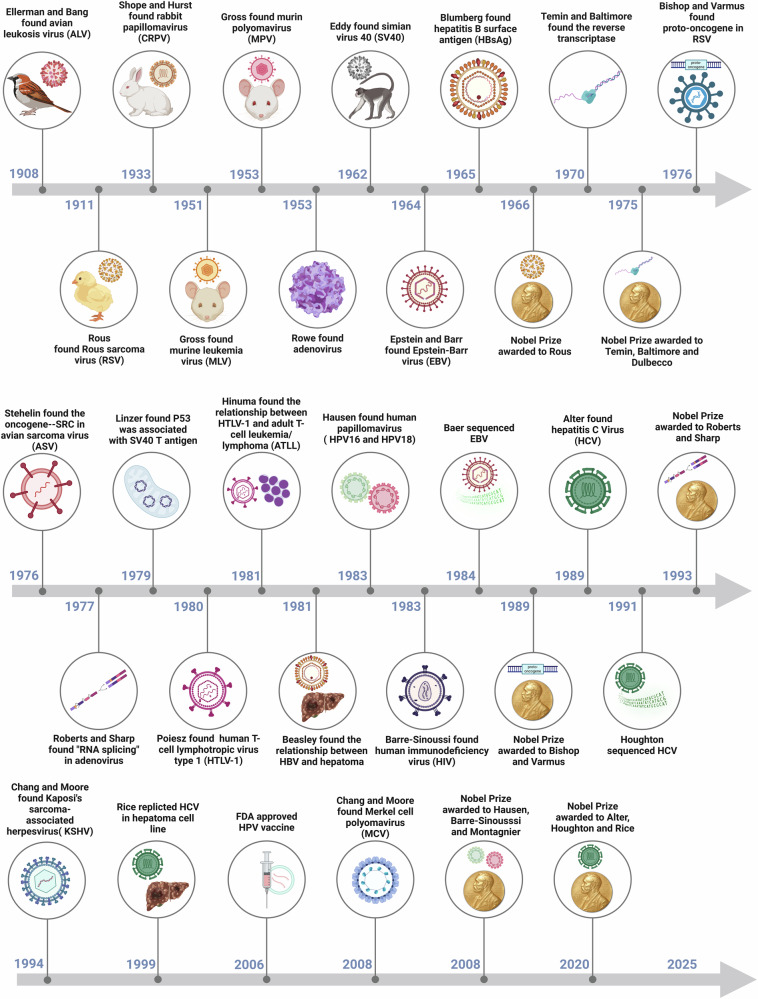
History of research and milestones in viral oncogenesis. The exploration embarked in the early 20th century. Initially, the discovery of the ALK laid the groundwork for oncovirus research. Subsequently, the RSV came to the fore, and in due sequence, the EBV and HBV emerged. A cascade of milestone accomplishments followed. For example, the identification of oncogenes revolutionized the field; the proposition that HPV induces cancer spurred the development of the cervical cancer vaccine. Moreover, viruses like HCV, KSHV, and MCV were unearthed. Collectively, these events have vividly traced the trajectory of the unceasing deepening of scientific research and the ceaseless efforts to battle cancer. This figure was created with BioRender.com

The discovery of proto-oncogenes by Bishop and Varmus in 1976, during their research on RSV, initiated the perception that viral infections cause cancer cell growth.^57^ After their research was published, one oncogene after another was discovered. They were awarded the 1989 Nobel Prize for this work. In 1976, Stehelin et al. identified the transforming gene, SRC, in avian sarcoma viruses (ASV) as a key oncogene in poultry.^57^ This study pioneered the discovery of the relationship between viral oncogenes and the host genome. In 1953, Rowe et al. discovered adenoviruses.^58^ In 1977, Roberts and Sharp discovered “RNA Splicing” in adenoviruses and were awarded the Nobel Prize in 1993. In 1979, Linzer et al. found that the tumor antigen p53 was associated with the replication and transformation of the SV40 virus.^59^ Lane et al. found that the SV40 virus T-antigen interacted with many proteins in the host cell, which may affect key biological processes.^60^ These two studies suggest the complexity of virus-host cell interactions and contribute to understanding the mechanisms of viral cancers. In 1980, Poiesz et al. identified HTLV-1 from T cell lymphoblastoid cell lines and cutaneous T cell lymphoma patients.^61^ In 1981, Hinuma et al. found an association between HTLV-1 and ATLL.^62^ In 1976, Hausen proposed the hypothesis that HPV was the leading cause of cervical cancer.^63^ In 1983, two high-risk viruses, HPV16 and HPV18, were identified in cervical cancer, thus laying a solid foundation for the development of the cervical cancer vaccine.^64^ In 2006, the FDA approved the HPV vaccine. Hausen, known as the “father of the HPV vaccine” was awarded the Nobel Prize in 2008. In 1983, Barré-Sinoussi et al. first reported the discovery of HIV.^65^ In 2012, the IARC identified HIV as an oncogenic virus, (IARC data, https://monographs.iarc.who.int/agents-classified-by-the-iarc/) associated explicitly with KS and Non-Hodgkin’s lymphoma. In 1989, Alter et al. cloned a cDNA sequence from a hepatitis patient, marking the discovery of HCV.^66^ In 1991, Houghton et al. successfully discerned the HCV genome.^67^ The 2020 Nobel Prize was awarded to Alter, Houghton, and Rice for their contributions to the discovery of HCV. Chang and Moore discovered KSHV in AIDS patients in 1994,^68^ and isolated KSHV from primary effusion lymphoma (PEL) cells in 1995.^69^ Subsequently, they discovered Merkel Cell Polyomavirus (MCV) in 2008.^70^ On the whole, viruses have contributed to our understanding of the molecular basis of oncogenesis (Fig. 1).

## The characteristics and infection mechanisms of oncogenic viruses

**Fig. 2:**
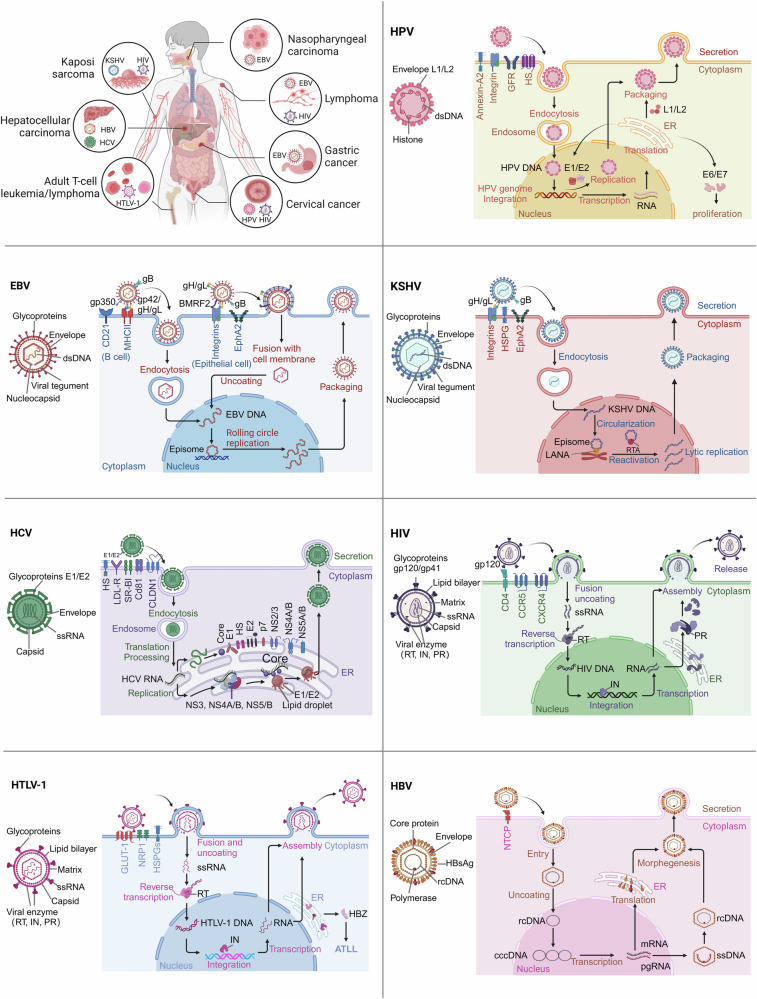
Types of virus-related cancer and mechanisms of viral infection. Major steps in the process of viral infection include Attachment, Entry, Uncoating, Replication and Transcription, Protein Synthesis, Assembly, and Release. Newly released viral particles can infect neighboring cells, continuing the infection process. Persistent viral infection increases the risk of virus-related cancer, including hepatocellular carcinoma, gastric cancer, cervical cancer, nasopharyngeal carcinoma, Kaposi sarcoma, lymphoma, and adult T-cell leukemia**/**lymphoma. Text in host cell color: intracellular components; Text in virus color: viral infection process; Black text: viral components. This figure was created with BioRender.com

Human oncogenic viruses exhibit a range of different characteristics.^71^ Some viruses have large double-stranded DNA genomes, such as EBV and KSHV. Others have small double-stranded DNA genomes, such as HPV and HBV. In addition, some viruses have positive-sense single-stranded RNA genomes, such as HCV, while HTLV-1 and HIV belong to the retrovirus family.^72^ Viral diversity is associated with different pathogenesis of DNA and RNA viruses, and this diversity contributes to rapid intrahost evolution.^73^ Most human oncogenic viruses infect their hosts with chronic monoclonal infections that persist for years after initial infection, which suggests that infection is only one factor in oncogenesis under a multifactorial environment and complex process (Fig. 2 and Table 2).^74^

**Table 2:** Summary of the oncogenic proteins and cancers caused by all oncogenic virus

Oncogenic Viruses	Oncoproteins	Target cells	Main target molecules	Function of viral proteins	Cancers	Ref
HBV	HBx, HBs Ag	Hepatocytes, bile duct epithelial cells, pancreatic cells, lymphocytes	p53, NF-κB, JAK/STAT	Inducing chronic inflammation, tissue injury, oxidative DNA damage, metastasis, and proliferation	Hepatocellular carcinoma, intrahepatic cholangiocarcinoma	^191,198^
HCV	Core, NS1-5	Hepatocytes, monocytes, macrophages, lymphocytes, bile duct epithelial cells	p53, NF-κB	Inducing ER stress, chronic inflammation, tissue injury	Hepatocellular carcinoma, Non-Hodgkin’s lymphoma	^259,270,983^
HPV	E5, E6, E7	Basal cells, spinous layer cells, genital, respiratory and oral mucosal epithelial cells	p53, pRB	Inhibiting cell cycle checkpoints and apoptosis	Cervix, vagina, vulva, anus, penis, intraepithelial neoplasia and a subset of head and neck cancer	^390,394,984^
EBV	LMP1, LMP2, EBNA-1,2	B lymphocytes, T lymphocytes, oropharyngeal epithelial cells, natural killer cells	NF-κB, JAK/STAT c-Myc	Inducing proliferation and resistance to apoptosis	B-lymphoproliferative diseases, T/NK-LPD, Burkitt lymphoma, Hodgkin lymphoma, diffuse large B-cell lymphoma, Plasmablastic lymphoma, T/NK cell lymphoma, nasopharyngeal carcinoma, gastric carcinoma, leiomyosarcoma, primary effusion lymphoma	^310,470^
KSHV	Viral interleukins viral chemokines LANA, K1	Vascular endothelial cells, lymphatic endothelial cells, lymphocytes, macrophages, oral and skin epithelial cells	p53 pRB GSK-3β	Inducing angiogenesis, cell proliferation, and cell transformation, and inhibiting cell cycle checkpoints	Kaposi sarcoma, primary effusion lymphoma	^515,520,985^
HTLV-1	Tax HBZ	Lymphocytes, monocytes, macrophages, dendritic cells, nerve cells	NF-κB	Inducing proliferation, senescence and genomic instability	Adult T-cell leukemia/lymphoma	^635,652^

### DNA virus

#### HBV

HBV is a partially double-stranded, hepatophilic DNA virus that primarily infects human hepatocytes, causing liver diseases.^75^ The HBV envelope encapsulates a nucleocapsid that contains partially double-stranded, relaxed circular DNA (rcDNA).^76^ The virus binds to the cell surface via pre-S glycoprotein and interacts with the primary receptor, the hepatic bile acid transporter sodium taurocholate cotransporting polypeptide (NTCP).^77^ Upon entry into hepatocytes, the rcDNA genome is translocated into the nucleus and converted into covalently closed circular DNA (cccDNA).^78^ Subsequently, cccDNA serves as a transcriptional template to form pregnenomic RNA (pgRNA) and messenger RNA (mRNA) in the presence of the regulatory HBV X protein (HBx).^79^ The pgRNA is selectively packaged into the nucleocapsid and serves as a template for the reverse transcription of HBV DNA in the cytoplasm of the liver, generating the core proteins and the polymerase.^80^ It also serves as a template for reverse transcription to produce new rcDNA. Viral envelope assembly takes place in multivesicular bodies (MVBs) to produce and secrete viral particles, which can also be recirculated into the nucleus to participate in cccDNA amplification and maintenance, so that cccDNA libraries can be sustained without requiring the entry of new HBV.^81^ Most acute HBV infections achieve viral clearance in a non-cytolytic manner (without directly killing infected hepatocytes), mainly involving cytokine-mediated inhibition of HBV replication via Interferon-gamma (IFN-γ) and tumor necrosis factor (TNF) secreted by virus-specific CD8^+^ T cells.^76^ In contrast, patients with chronic HBV infection usually pursue a lifelong course.^82^

#### HPV

HPV is a non-enveloped, double-stranded, circular DNA virus of the *papillomavirus* family comprising three regions: the long control region (LCR), the early region encoding virions (E1-E7), and the late region encoding virions (L1 and L2).^83^ More than 200 HPV genotypes have been identified, and these include mainly high-risk types (HPV16, HPV18), which cause cancer, and low-risk types (HPV6, HPV11), which do not cause cancer.^84^ Despite discrepancies in the size and number of open reading frames (ORFs) of different HPV molecules, all HPVs contain conserved core genes that are involved in viral replication (E1 and E2) and packaging (L1 and L2); the rest of the genes (E4, E5, E6, and E7) function in driving the cell cycle, immune escape, and viral release.^22^ HPV predominantly infects basal epithelial cells, binding to the extracellular matrix (ECM) or cell surface via L1.^3,85^ This process has been shown to involve multiple receptors/co-receptors, including heparan sulfate (HS), α6β4 integrin, growth factor receptors and annexin-A2.^86,87^ Upon internalization, the capsid is disassembled in endosomes, and the multifunctional L2 protein directs viral DNA to the host cell nucleus.^88,89^ Once infection is established in the cell, the viral life cycle is regulated to maintain host-viral protein homeostasis.^90^ Most HPV infections are self-limiting, while persistent high-risk HPV infections can increase the risk of cervical intraepithelial neoplasia (CIN) and CC.^91^

#### EBV

EBV is a γ-herpesvirus whose structure consists mainly of a core and an envelope. The core contains a linear double-stranded DNA involved in viral replication and transcription. The envelope consists of a variety of glycoproteins (gp350/gp220 and gp42) and membrane proteins (BLLF1, glycoprotein B (gB), and glycoprotein H/glycoprotein L (gH/gL)), which mediate the viral entry into the host cell.^92^ The target cells of EBV are B lymphocytes and epithelial cells, and its mechanism for entering host cells is similar to that of other members of the *herpesvirus* family.^93^ The infection of B cells by EBV is initiated by the binding of the EBV envelope protein gp350 to B cell surface receptor CD21. Upon binding to CD21, EBV gp42 interacts with the host cell surface MHC-II, resulting in its binding to the heterodimerization protein gH/gL. EBV gH/gL then activates the EBV fusion protein gB, which directly mediates the fusion of the virus with the host cell membrane. At this point, the viral glycoprotein gB and the heterodimeric gH/gL form the core of the EBV fusion machinery, which ultimately allows the virus to be translocated into the nucleus.^94,95^ Upon EBV infection of epithelial cells, EBV BMRF2 binds to integrins. Subsequently, gH/gL binds to integrins and ephrin receptor A2, triggering activation of gB and fusion of the viral envelope to the plasma membrane of the epithelial cell.^96^ EBV infection is categorized into latent and lytic infections. During latent infection, four modes are classified as latency III, latency II, latency I, and latency 0 based on how these genes are expressed in different combinations in EBV-infected cells, which is characterized by a gradual restriction of the viral gene expression pattern to evade immune surveillance.^97^ Eventually, EBV establishes a persistent residency in memory B cells characterized by a lack of viral antigen expression (latency 0), thus evading T cell recognition and acting as a viral reservoir.^98^ EBV can periodically transit to the lytic cycle, leading to viral replication, shedding, and subsequent dissemination, which is involved in B cell transformation and oncogenesis.^95,99^ EBV exhibits four distinct latency phases, each contributing uniquely to carcinogenesis.^100^ In latency III, expressing all 6 latent genes, it is implicated in severely immunosuppressed diffuse large B-cell lymphoma.^101^ Latency I, with only EBNA1 expressed, aids in maintaining the EBV genome and is associated with cancers like Burkitt lymphoma.^102^ Latency II, expressing EBNA1 and LMPs, correlates with cancers like nasopharyngeal carcinoma, where LMP1 impacts cell growth and apoptosis.^102^ Even latency 0, despite having no antigen expression, might play a part in cancer development under specific circumstances.^103^ Besides latency, EBV’s lytic replication also plays a role in carcinogenesis. Some lytic proteins, like BZLF1 and BRLF1, can activate genes involved in DNA amplification and virus production, potentially disrupting normal cell functions and fueling cancer development.^104^

#### KSHV

KSHV, also known as human herpesvirus 8 (HHV-8), belongs to the γ-*herpesvirus* family.^105^ KSHV is a linear double-stranded DNA virus with a central coding region surrounded by two sides of non-coding terminal repeat units with high GC content on both sides.^106^ KSHV is a broadly cytophilic virus that can infect B cells, fibroblasts, epithelial cells, and endothelial cells.^51,107^ The glycoprotein gB on the viral envelope mediates virus-cell binding and entry by interacting with HS and entry receptors on the surface of host target cells.^108^ After the virus enters the host cell, the capsid of the virus particle detaches and releases its genome into the cytoplasm, followed by the import of the KSHV genome into the nucleus.^109^ KSHV is often found in the host in both latent and lytic states, and the latency-associated nuclear antigen (LANA) is the essential protein that maintains its latency. The latent KSHV can attach its genome to the host chromosome and be retained as a free body mediated by LANA.^110^ However, various stimuli can cause the virus to withdraw from latency. The regulator of transcription activator (RTA) is a nuclear DNA-binding protein that regulates the transition of the virus from latency to lytic replication. The expression of RTA initiates the KSHV gene expression in a lytic cascade. Subsequently, the replication of the genome and the production of new viruses are facilitated.^111^ Chronic infection with KSHV is one of the etiologic causes of KS, B cell lymphoma and PEL.^112^

### RNA virus

#### HCV

HCV is a single-stranded, positive-sense hepatotropic RNA virus, consisting of an envelope and a nucleocapsid. The main components of the envelope are glycoproteins E1 and E2, which mediate the internalization of virus particles.^113,114^ E1 acts as a fusion protein during internalization, while E2 is responsible for host cell receptor binding.^113,115,116^ Once internalized, the viral RNA is released and translated into a variety of viral proteins, including three structural proteins (Core, E1, and E2) and seven non-structural proteins (p7, NS2, NS3, NS4A, NS4B, NS5A, and NS5).^117^ The structural proteins are used for the assembly of the zygotic virosome.^118^ In contrast, non-structural proteins can bind to lipid droplets, forming highly lipidated lipoviral particles that enable viral replication and transmission.^119^ Various errors are observed during the replication process, mainly attributable to the uncorrected function of RNA polymerase and the absence of an effective mechanism for error correction.^120^ This ultimately contributes to the high degree of heterogeneity observed in HCV.^121^ Additionally, the envelope protein E2/NS1 region, which stimulates the production of neutralizing antibodies, exhibits the highest degree of variability, resulting in a robust capacity of the virus to evade adaptive immunity.^122^ In the acute phase of infection, the clearance of viral particles is predominantly contingent upon intrinsic immunity. The lysis and clearance of most HCV-infected cells is mediated by perforin and granzyme B, which are released by cytotoxic T-lymphocytes (CTLs) and natural killers (NKs), respectively.^123^ A minority of HCV-infected cells are cleared non-lytically by IFN-γ secreted by CTLs and NK cells.^124^ The majority of individuals infected with HCV will eventually progress to chronic infection. The proliferation of HCV-specific CTLs during chronic infection is impaired, accompanied by a diminished ability to produce IFN-γ and an increased susceptibility to exhaustion. This increases the probability that the patients will develop HCC.^125,126^

#### HIV

HIV is a single-stranded RNA virus that predominantly infects CD4^+^ T cells, macrophages, dendritic cells, and others.^127,128^ HIV comprises two distinct components: the Gag polyprotein and the envelope. Gag polyproteins are composed of a capsid protein (CA),^129,130^ which contains two identical viral single-stranded positive-stranded RNAs; a nucleocapsid protein (NC); and a collection of enzymes essential for viral replication, including reverse transcriptase (RT), integrase (IN), and protease (PR).^131^ The outermost layer of the virus is the envelope, which is embedded with the outer membrane glycoprotein gp120 and the transmembrane glycoprotein gp41. Beneath the envelope structure are matrix proteins (MA) that form the viral inner capsid.^132^ Following the binding of the viral outer membrane protein gp120 to the CC-chemokine receptor/CXC chemokine receptor 4 (CCR5/CXCR4) on the membrane surface of the target cell, the virus initiates its invasion of the target cell. Upon entering the target cell, the virus releases its NC.^133,134^ With the effect of RT, HIV RNA is reverse-transcribed in the host cytoplasm to form DNA, which further migrates into the host cell nucleus.^135^ However, as RT cannot correct errors, the HIV genome is susceptible to mutation during the reverse transcription process.^136^ Typically, most of the viral genome is integrated into silent and non-replicating genomic regions that serve as long-term HIV reservoirs without being destroyed by immune surveillance. Nevertheless, minor quantities of viral DNA may integrate into more active genome regions.^137^ Finally, Gag drives the assembly and release of nascent HIV particles and causes damage and exhaustion of target cells.^131,138^ The course of HIV infection can be categorized as acute, asymptomatic, or AIDS phases. With the use of antiretroviral therapy (ART), there has been a notable reduction in people living with HIV (PLWH) progressing to the AIDS Phase.^139,140^ However, the persistent damage to the immune system caused by HIV infection still increases the risk of cancer in PLWH.^141^

#### HTLV-1

HTLV-1 is a delta retrovirus that infects peripheral blood mononuclear cells.^142,143^ Like most retroviruses, HTLV-1 is composed of an envelope and a capsid. The capsid contains enzymes essential for viral replication and a nucleocapsid with the viral genome.^144^ HTLV-1 is mainly transmitted through cell-to-cell contact. Virological synapse, a key structure in this process, promotes the binding of HTLV-1 viral envelope glycoproteins to specific receptors on target cells and mediates the endocytosis of viral particles into cells.^142^ Neuropilin 1 (NRP1), glucose transporter 1 (GLUT-1) and heparan sulfate proteoglycans (HSPGs) are HTLV-1-specific receptors.^145,146^ With the fusion of the viral envelope with the cytoplasmic membrane, HTLV-1 RNA is reverse transcribed into HTLV-1 DNA and integrated into the host DNA, which is transcribed and translated along with the host DNA.^147^ HTLV-1 DNA contains multiple coding regions and long terminal repeats (LTRs) located at the 5’ and 3’ ends of the genome. The 5’ LTR cis-sequence contains all viral structural genes and most regulatory genes; the 3’ LTR is a reverse transcription of the original viral gene that produces the essential zipper protein (HBZ).^148^ HBZ, a highly conserved protein, is expressed in all HTLV-1-infected cells and is vital for maintaining the malignant phenotype of ATLL.^149,150^ HTLV-1 replication is active only during the initial stages of infection, after which it transitions to a latent state.^151^ The vast majority of HTLV-1-infected individuals remain carriers for long periods or for life; only a minority develop ATLL or lymphoma.^152^

## Oncogenic mechanisms in specific cancer types

**Fig. 3:**
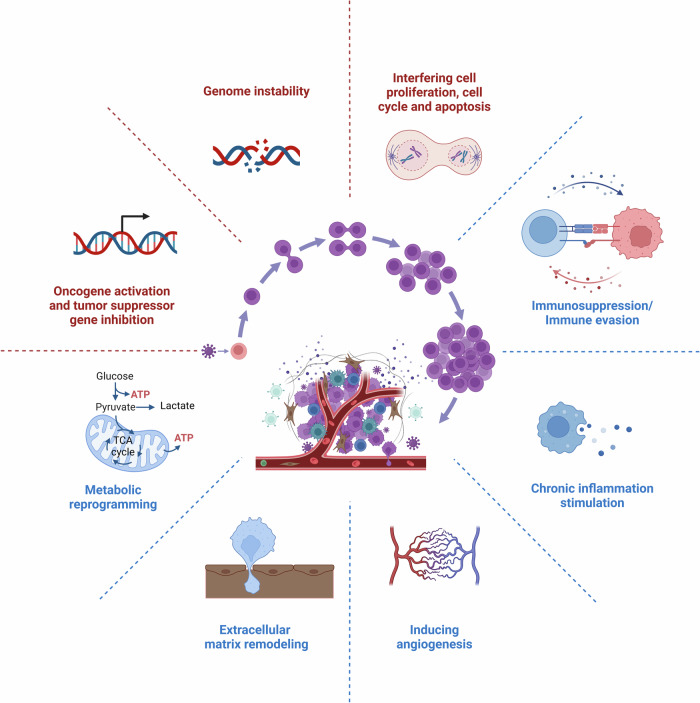
An overview of viral oncogenic mechanisms. Oncoviruses induce cellular transformation by hijacking the homeostasis of host cells. The specific mechanisms include: oncogene activation and tumor suppressor gene inhibition; induction of genomic instability; interfering with cell proliferation, cell cycle and apoptosis; immunosuppression and immune evasion; chronic inflammation stimulation; extracellular matrix remodeling; inducing angiogenesis; metabolic reprogramming. These mechanisms work together to drive tumor formation and metastatic spread. Red text: direct oncogenesis; Blue text: indirect oncogenesis. This figure was created with BioRender.com

Oncoviruses induce cellular transformation by hijacking host cell homeostasis.^6^ They mainly exert their biological functions by encoding oncogenic proteins of viral origin, and the specific mechanisms include: (1) Activation of oncogenes or inactivation of tumor suppressor genes: Oncoviruses hijack host cells and induce tumorigenesis by activating oncogenes or inactivating tumor suppressor genes.^1^ (2) Inducing genomic instability: Oncoviruses trigger genomic instability in host cells, including point mutations, insertions/deletions, structural variations, and DNA damage.^153^ (3) Interference with normal cell life processes: Oncoviruses disrupt critical signaling pathways, such as cell proliferation, cell cycle regulation, and apoptosis, leading to abnormal host cell growth and proliferation.^5^ For instance, EBV latency proteins LMP1, LMP2a, EBNA2, EBNA3A, EBNA3B, EBNA3C, and EBNA LP intersect and manipulate several cellular signaling pathways, which ultimately contribute to EBV-mediated B cell proliferation.^154,155^ (4) Evolving immune evasion strategies: Oncoviruses evolve multiple immune evasion strategies, including inhibition of antigen presentation, upregulation of immune checkpoint molecules like PD-L1, and activation of regulatory T cells, thereby suppressing the immune system’s ability to effectively recognize and eliminate tumor cells.^156,157^ (5) Persistent inflammation and oxidative stress: Oncoviruses promote the release of inflammatory factors and chemokines, leading to a disrupted cellular microenvironment, and induce abnormal oxidative stress, resulting in oxidative damage to DNA, proteins, and lipids, further driving the carcinogenic process.^158,159^ (6) Remodeling the ECM: Oncoviruses alter the composition and structure of the ECM, affecting the surrounding microenvironment and promoting tumor growth and invasion.^160–162^ (7) Regulating angiogenesis: Oncoviruses regulate angiogenesis-related signaling pathways. For instance, the oncoproteins E6 and E7 of HPV can induce the expression of vascular endothelial growth factor (VEGF), which promotes the formation of new blood vessels.^163–165^ (8) Oncoviruses induce metabolic changes, including enhancing glycolysis and lipid metabolism, to support the rapid growth and proliferation of transformed cells.^166,167^ For example, HPV early-phase proteins E1, E2, E5, E6 and E7 can activate plethora of metabolic pathways and directly influence enzymes of the glycolysis pathway to promote the Warburg effect by increasing glucose uptake, activating glycolysis and pentose phosphate pathway.^168^ Generally, the complex and unique features that drive tumor formation and metastatic spread are summarized into ten hallmarks of cancer, including self-sufficiency in growth signals, insensitivity to anti-growth signals, resisting cell death, limitless replicative potential, sustained angiogenesis, tissue invasion and metastasis, avoiding immune destruction, tumor-promoting inflammation, deregulating cellular energetics, and genome instability and mutation (Fig. 3).^169,170^

### Hepatocellular carcinoma (HCC)

#### HBV-related HCC

**Fig. 4:**
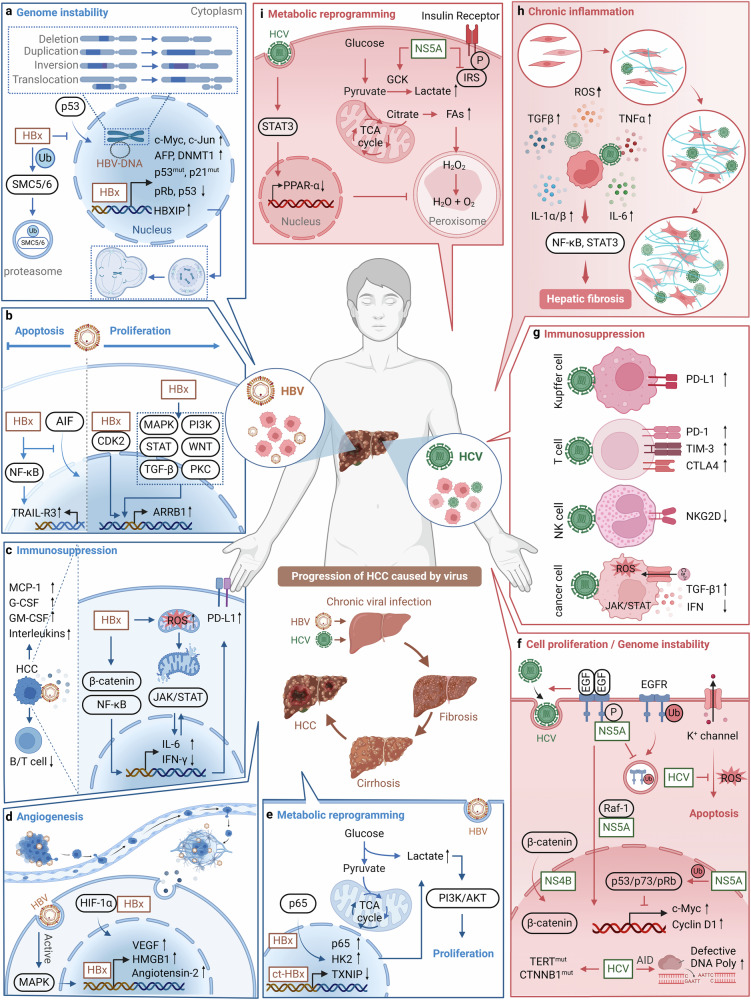
Oncogenic mechanisms of HBV and HCV in hepatocellular carcinoma. **a** HBV infection causes chromosomal abnormalities, mitotic defects, and increased genetic mutations. **b** HBx activates glycolysis via the NF-κB/HK2 pathway, and excess lactic acid enhances HCC proliferation through the PI3K/AKT pathway. **c** HBV evades T cell responses by enhancing the expression of PD-L1 in HCC cells, and its related factors construct an inflammatory microenvironment while the infection leads to ROS accumulation and triggers oxidative stress. **d** HBV promotes angiogenesis in HCC by increasing HIF-1α, HMGB1, and VEGF expression, and activates MAPK pathways to facilitate metastasis. **e** HBx protein upregulates p65 and HK2, downregulates TXNIP to enhance host cell glycolysis. **f** HCV promotes HCC proliferation by inhibiting EGFR degradation, activating Wnt/β-catenin signaling, inducing c-Myc and cyclin D1 expression, blocking K^+^ channels, and enhancing the expression of abnormal DNA polymerase. **g** HCV suppresses IFN signaling, promotes TGF-β1 secretion, and increases inhibitory receptor expression on immune cells, thereby dampening the immune response. **h** HCV infection enriches inflammatory factors in the TME, contributing to liver fibrosis. **i** HCV can enhance glycolysis, increase the accumulation of long-chain fatty acids, and interfere with insulin signaling. Blue part: HBV carcinogenic mechanism; Red part: HCV carcinogenic mechanism; Text in virus color: viral components; Black text: host cell components; Direct oncogenesis: **a**, **b** (HBV), **f** (HCV); Indirect oncogenesis: **c**, **d** (HBV), **g**–**i** (HCV). This figure was created with BioRender.com

HBV is the most diverse DNA virus and its genome consists of four open reading frames (ORFs): the presurface antigen/surface antigen gene (preS/S), the precore/core gene (preC/C), the polymerase gene (P), and the X gene (X).^171^ HBV infection leads to liver fibrosis and cirrhosis, and ultimately to HCC.^172^ Integration of viral DNA into the host genome is thought to be the initiating event for HBV-induced carcinogenesis, with integration preferentially targeting promoters and coding regions.^173^ In HCC, the most common recurrent HBV DNA integrations occur at the telomerase reverse transcriptase (TERT), mixed-lineage leukemia 4 (MLL4), cyclin E1 (CCNE1), and fibronectin (FN1) loci.^174^ Integration is amplified when integrating cells undergo mitosis. This allows HBV DNA integration to persist and become a major source of HBV antigen in the later stages of chronic infection.^175^ Although HBV DNA integration is often unable to transcribe pre-core mRNAs and pgRNAs due to loss of the upstream basic core promoter, the promoters of the S and X ORFs are intact, allowing synthesis of functional HBsAg and C-terminally truncated HBx proteins.^176^ Since HBx is mainly localized in the cytoplasm, it regulates multiple signaling pathways, such as mitogen-activated protein kinase (MAPK), NF-κB, rat sarcoma virus (Ras), rapidly accelerated fibrosarcoma (Raf) and JAK-STAT.^177–180^ HBx is also involved in cell cycle regulation, DNA repair, apoptosis, and immunosuppression, and plays an important role in HCC development (Fig. 4).^181^

##### Direct oncogenesis: leading to the emergence of virus-related tumors

*Oncogene activation and tumor suppressor gene inhibition:* Proto-oncogenes regulate the cell cycle, control cell growth, and division, and their abnormal activation can lead to uncontrolled cell proliferation, ultimately causing cellular transformation into cancer.^182^ HBx protein can trans-activate multiple proto-oncogenes, including c-Myc and c-Jun, thereby contributing to HCC development.^178,183–186^ HBV DNA integration can also transform proto-oncogenes into oncogenes or cause mutations in tumor suppressor genes, leading to the loss of their normal biological functions, disrupting normal hepatocyte proliferation, and causing HCC development.^187^ Tumorigenesis is closely associated with oncogene activation and the functional inhibition of tumor suppressor genes.^188^ The p53 protein is a well-known tumor suppressor that plays a crucial role in promoting apoptosis, repairing DNA damage, and other biological functions. The downregulation or inactivation of p53 is often linked to tumor formation.^189^ Research has shown that the HBx protein can bind to p53 in the cytoplasm, forming a complex that hinders p53’s translocation to the nucleus, thereby reducing its nuclear concentration and impairing its ability to induce apoptosis.^190^ The C-terminal domain of p53, which is responsible for binding to damaged DNA, is competitively bound by HBx, obstructing p53’s DNA repair function.^191^ Additionally, HBx binding to the C-terminal of p53 leads to the inactivation of the promoter of another tumor suppressor gene, pRb, resulting in cell cycle alterations that signal the onset of carcinogenesis. Collectively, HBx protein inhibits the activity of tumor suppressor genes p53 and pRb through multiple pathways, promoting the development of HCC (Fig. 4a).

*Genome instability:* Random integration of HBV DNA into the human genome contributes to host genomic instability, including chromosomal deletions, amplifications, breaks, inversions, and translocations.^192^ During the early stages of clonal tumor expansion, HBV integrates into the host genome at sites of double-stranded DNA breaks, leading to cis-acting interference, chromosomal instability, and the expression of mutated HBV genes, which are characteristic of HCC cells.^193^ HBx affects centrosome replication and cell division by regulating the hepatitis B X-interacting protein (HBXIP), leading to mitotic abnormalities and loss of genetic stability, thereby promoting HCC development.^194^ HBx also interacts with TAX1-binding protein 2 (TAX1BP2), disrupting centrosome-microtubule dynamics and leading to chromosomal instability.^195^ For proper chromosomal duplication and segregation, the higher-order chromosome structure must be organized by structural maintenance of chromosomes (SMC) complexes, which include cohesin, condensin, and SMC5/6.^196^ HBx binds to the cellular E3 ligase complex containing DNA damage-binding protein 1 (DDB1), leading to the degradation of SMC 5/6 proteins and promoting host cell genetic instability.^197,198^ Additionally, HBx interacts with the epigenetic regulator WD repeat domain 5 protein (WDR5), affecting H3K4me3 modification and further influencing HBV transcription and HCC progression (Fig. 4a).^199^

*Interfering cell proliferation, cell cycle and apoptosis:* HBx interferes with cell proliferation, cell cycle, and apoptosis through various mechanisms during the occurrence of HCC. First, HBx can activate multiple molecular pathways and critical proteins involved in cell proliferation, including (MAPK)/Ras/Raf/c-Jun, NF-κB, JAK-STAT, protein kinase C, SMAD4, DEAD-box RNA helicase 17 (DDX17)/ZW10 interacting protein (ZWINT), Src, decorin, decorin-derived peptides, survivin, and PI3K cascades.^200–203^ Additionally, HBx can shift hepatocytic TGF-β signaling from the tumor-suppressive pSmad3 pathway to the oncogenic pSmad3 pathway during the early stages of carcinogenesis.^204,205^ Other studies have found that HBx promotes hepatocyte division by upregulating cyclooxygenase-2 (COX-2), 5-lipoxygenase (5-LOX), and phosphorylated extracellular signal-regulated protein kinases 1/2 (p-ERK1/2).^206^ Additionally, HBx can regulate various miRNAs and lncRNAs, leading to pathway dysregulation, including RB1-TP53, PI3K-MAPK, WNT-β/catenin, and JAK/STAT, which are involved in cell proliferation, angiogenesis, and metastasis.^190,191^.

HBx can extend the S phase of the cell cycle by binding to DDB1, inducing mitotic abnormalities and genomic instability in hepatocytes, which promotes HCC development.^207,208^ Additionally, HBx can directly interact with cyclin-dependent kinase 2 (CDK2), promoting HCC development. This interaction induces phosphorylation of ubiquitin-like with PHD and ring finger domains 2 (UHRF2) at serine 643, inhibiting UHRF2 ubiquitination.^209^ CDK2 is a cyclin-dependent kinase, and UHRF2 is a nuclear protein involved in cell cycle regulation, both playing critical roles in the development of HCC.^210,211^ Furthermore, HBx can upregulate arrestin beta 1 (ARRB1) protein, which drives HBV-related HCC by regulating autophagy and the CDKN1B-CDK2-CCNE1-E2F1 axis.^212^

Research indicates that HBx can inhibit hepatocyte apoptosis and increase HBV replication by activating the NF-κB signaling pathway, leading to the upregulation of TNF-related apoptosis-inducing ligand receptor 3 (TRAIL-R3), thereby providing favorable conditions for HCC development.^213^ HBx also interacts with apoptosis-inducing factor (AIF) and the homologous AIF-homolog mitochondrion-associated inducer of death (AMID), inhibiting the translocation of AIF from the mitochondria to the nucleus, resulting in a significant downregulation of caspase-independent apoptosis pathways.^214^ The apoptosis-inducing effects of HBx can vary, partly due to HBx mutations, such as C-terminal truncation (trHBx).^215^ Additionally, HBx can influence cell proliferation, migration, apoptosis, and the cell cycle by regulating centromere protein M (CENPM), thereby promoting HCC development.^216^ Known HBx mutants can also increase the risk of HCC development. For instance, the HBx mutation F30V, localized in the HBx N terminus, enhances the anti-apoptotic activity of HBx in vitro, promoting the survival of infected hepatocytes and thereby initiating HBV-driven HCC (Fig. 4b).^217^

##### Indirect oncogenesis: promoting the progression of virus-related tumors

*Immunosuppression:* HBx primarily exerts its immunosuppressive effects by increasing HBV replication, stabilizing cccDNA, and inhibiting IFN production in the host.^218^ HBx can enhance PD-L1 expression by modulating the PTEN/β-catenin/c-Myc signaling pathway, inhibiting T-cell responses and promoting HBV immune evasion.^219^ HBV-specific CD8^+^ T cells exhibit T cell exhaustion in patients with chronic infection, impairing their ability to clear the virus effectively, thus failing to control viral replication.^220–222^ HBx has also been reported to interact with Spindlin1, shifting chromatin from an H3K9me3-repressed state to an H3K4me3-active state, thereby promoting the transcription of the HBV chromatin genome.^81^ Additionally, the HBx protein can bind to cccDNA microchromosomes, playing a crucial role in initiating and maintaining cccDNA transcription, with accumulated cccDNA contributing to HCC development.^81,223^ HBx downregulates IFN-γ production by targeting critical signaling components, including RIG I, TRAF3, Cardif, TRIF, Nemo, TBK1, IKKε, interferon regulatory factor 3 (IRF3), and cGAS.^224–226^ This interference by HBx helps create a favorable environment for HBV replication and survival within the host. Furthermore, HBx has been shown to directly bind to and impair the function of IFN-γ, counteracting the antiviral activity of MHC II transactivator (CIITA). This may represent a novel immune evasion mechanism employed by HBx, further illustrating its multifaceted approach to hindering host immune responses.^227^

The host’s innate and adaptive immune systems play a crucial role in clearing HBV after infection. However, HBV has evolved and developed effective strategies to evade the host’s immune surveillance, resulting in persistent infection.^228^ Most HBV infections occur in neonates with immune deficiencies, characterized by lower quality and quantity of HBV-specific T cells and B cells. In addition, maternal HBeAg can induce PD-L1 upregulation in Kupffer cells (KCs) in offspring, thereby inhibiting the HBV-specific CD8^+^ T cell response and thus supporting the persistence of HBV after birth.^229^ In chronic viral hepatitis, the upregulation of immune checkpoint, including PD-1/PD-L1, CTLA-4 and TIM-3, plays an important role in immunosuppression by inhibiting the T cell response.^230^ HBV infection induces immunosuppression, and then peripheral immune tolerance occurs as chronic infection progresses; finally, due to impaired immune surveillance, it mediates the occurrence of HCC (Fig. 4c).^231^

*Chronic inflammation stimulation:* Accumulation of ROS and excessive oxidative stress are detected in almost all cancers and are considered to induce tumor initiation and progression.^232^ ROS production induced by the C-terminal region of HBx leads to mitochondrial DNA damage in hepatocytes, which may contribute to HCC development.^233^ During HBV-induced inflammatory responses, the HBx protein can upregulate the transcription level of interleukin-6 (IL-6) by activating the NF-κB pathway.^234^ Activated IL-6 can trigger the JAK/STAT signaling pathway, leading to the upregulation of VEGF expression, thereby promoting angiogenesis.^235^ Additionally, the activated STAT pathway can induce the overproduction of IL-6 and other oncogenic inflammatory cytokines or chemokines, creating a vicious cycle that ultimately leads to HCC.^236^ Several other inflammatory factors, such as granulocyte colony-stimulating factor (G-CSF), granulocyte-macrophage colony-stimulating factor (GM-CSF), VEGF, monocyte chemoattractant protein-1 (MCP-1), and interleukins, contribute to the inflammatory microenvironment essential for HCC development.^237^ These inflammatory factors promote other cancer hallmarks by delivering bioactive molecules to the microenvironment, such as anti-apoptotic survival factors, growth factors, ECM-modifying enzymes driving angiogenesis, pro-angiogenic factors, and signals that facilitate cellular transformation, thereby promoting tumorigenesis.^170,238^

Non-resolving inflammation is driven by external factors (e.g., pathogen-associated molecular patterns (PAMPs) released by gut microbiota) and internal factors (e.g., damage-associated molecular patterns (DAMPs) released by apoptotic hepatocytes). These DAMPs include nuclear and cytoplasmic proteins, such as histones, IL-1, high-mobility group box 1 (HMGB1), and heat shock proteins (HSPs). Intrinsic pathways (including those where the tumor itself attracts inflammatory cells) also contribute to HCC development through chronic inflammation stimulation and oxidative stress induced by HBV.^239,240^ In summary, HBV-induced chronic inflammation and oxidative stress increase the risk of HCC. Persistent inflammatory states lead to the continuous release of inflammatory factors, promoting abnormal cell proliferation. Oxidative stress causes DNA damage, further exacerbating the carcinogenic process in hepatocytes (Fig. 4c). Since the liver is directly connected to the intestines through the hepatic portal vein circulation, the pathogenesis of HCC is associated with alterations in the gut microbiota.^241^ Compared with non-viral-related HCC patients, those with HBV-related HCC exhibit significantly greater richness in gut microbiota species.^242^ In patients with HBV-related HCC, there is a notable shift in the bacterial composition of the gut. Specifically, there are more potentially anti-inflammatory bacteria, such as *Prevotella* and *Faecalibacterium*, and fewer pro-inflammatory bacteria, like *Escherichia-Shigella* and *Enterococcus*. This divergence strongly implies that the gut microbiota plays a crucial role in the progression of HBV-related HCC.^242^

*Inducing angiogenesis:* HBx promotes angiogenesis through various pathways, a critical process in the growth and metastasis of HCC.^243^ Studies indicate that HBx enhances the expression of HMGB1, promoting epithelial-mesenchymal transition (EMT) and angiogenesis in HBV-related HCC.^244^ Furthermore, HBx stabilizes and activates hypoxia-inducible factor 1-alpha (HIF-1α) and the MAPK pathway, leading to increased VEGF expression and angiogenesis.^245^ HBx also cooperates with lysine-deficient protein kinase-1 (WNK1) to promote tumor-induced angiogenesis. Specifically, WNK1 activates downstream effectors oxidative stress-response kinase-1 (OSR1) to stimulate the migration of endothelial cells and HCC cells^246^ These findings highlight the complex molecular regulatory mechanisms through which HBx, including HMGB1, HIF-1α, WNK1, and their downstream effectors, plays a crucial role in promoting angiogenesis (Fig. 4d).

*Metabolic reprogramming:* Viral infections often alter the energy metabolism of host cells to support their replication and survival.^247^ This metabolic reprogramming may involve enhanced glycolysis, altered lipid metabolism, impact on calcium channels, and regulation of mitochondrial function, providing essential nutrients and energy for the virus while promoting its replication and pathogenicity.^248^ Studies have found that C-terminal truncated HBx (Ct-HBx) initiates HCC by downregulating thioredoxin-interacting protein (TXNIP), a key regulator of glucose sensing and the redox system, and reprogramming glucose metabolism.^249^ TXNIP is considered a tumor suppressor, and its expression is low or redundant in HCC cells.^250^ Additionally, HBx induces lactate fermentation metabolism by activating the NF-κBp65/hexokinase 2 (HK2) signaling pathway, with excessive lactate significantly promoting HBx-induced spontaneous HCC via the PI3K/AKT pathway.^178^ HBx also enhances insulin signaling sensitivity by promoting the expression of HER2, thereby facilitating HCC cell growth and migration.^251^ Moreover, HBx stabilizes glucose-regulated protein 78 (GRP78) through TRIM25, promoting the expression of mannosidase alpha class 1B member 1 (MAN1B1), a process critical in HBV-induced tumorigenesis. Additionally, HBx amplifies TRPM7-mediated calcium influx through hsa-miR-128-3p/SPG21, thereby activating the c-Jun N-terminal kinase (JNK) pathway and contributing to liver oncogenesis (Fig. 4e).^252^

#### HCV-related HCC

Chronic HCV infection can also cause progressive liver fibrosis, cirrhosis, and HCC.^253^ Unlike other oncogenic viruses, HCV does not integrate into the host genome or stably exists as an episome. Instead, it maintains its presence by forming complexes with low-density lipoproteins and other molecules.^254^ During natural infection, HCV typically exhibits transient replication within individual cells but requires continuous infection of new cells to establish persistent infection.^255^ HCV produces five proteins associated with oncogenesis: the core protein and four nonstructural proteins (NS3, NS4B, NS5A, and NS5B). Specifically, the core protein can regulate intracellular pathways (e.g., NF-κB) and upregulate various cellular proteins (e.g., IL-6, STAT-3), whose dysregulation may induce hepatocyte transformation.^256^ The nonstructural proteins of HCV also have oncogenic effects by inhibiting hepatocyte apoptosis and increasing cellular proliferation.^257^ HCV-related HCC is typically the result of chronic inflammation and fibrosis caused by long-term infection, a process that may take decades.^258^

##### Direct oncogenesis: leading to the emergence of virus-related tumors

*Oncogene activation and tumor suppressor gene inhibition:* The oncogene epidermal growth factor receptor (EGFR) assists HCV in entering host cells. EGFR signaling is active during ligand-induced receptor dimerization and internalization and is regulated by phosphatases and endosomal recycling/degradation.^259^ HCV has evolved mechanisms to exploit EGFR signaling to enhance its survival and replication. HCV infection induces EGFR signaling and alters the expression of other erythroblastic oncogene B (ERBB) receptors in early endosomes via NS5A, sustaining EGFR signaling and leading to the accumulation of more EGFR in infected cells.^260^ HCV-NS5A promotes viral replication by binding to Raf-1 kinase, indicating that HCV infection leads to direct virus-host dependencies and pathway-related transcriptional changes.^261^ In summary, oncogene activation is a crucial strategy for HCV to hijack host cell signaling pathways and drive malignant transformation.

Like other oncogenic viruses, one of the functions of the HCV core protein is to inactivate tumor suppressors, such as p53 and pRb, to enhance genomic instability and induce uncontrolled cell proliferation.^262^ The core protein can bind to tumor suppressor proteins p53, p73, and pRb,^263^ while NS3 and NS5B bind to pRb, interfering with its suppressive function.^264,265^ NS5A physically interacts with p53 in vitro and in vivo, sequestering p53 in the perinuclear membrane. The subsequent reduction in nuclear p53 may lead to the downregulation of p53-mediated gene expression necessary for normal cell growth.^266^ NS5B interacts with RB in the perinuclear region, forming a stable complex and degrading it before its transport to the nucleus. Conversely, the HCV core protein appears to lower pRb levels by downregulating RB mRNA.^267^ Collectively, the HCV core protein and nonstructural proteins enhance genomic instability and promote cell proliferation by binding to and interfering with tumor suppressor proteins (Fig. 4f).

*Genome instability:* HCV can induce genomic instability in at least three ways: (i) by directly interfering with mitotic regulatory proteins, (ii) by integrating the viral genome into multiple sites in the host genome, and (iii) by causing persistent inflammation in the liver.^268^ The mutational profile of HCV-related HCC generally lacks specificity, but drive mutations in the telomerase reverse transcriptase (TERT) promoter and CTNNB1 (β-catenin) are ubiquitous.^269,270^ Perez et al. found an enrichment of C- > T transcriptional mutations in HCV-related HCC, suggesting that this contributes to the increased susceptibility to HCC in chronic HCV-infected individuals.^271^ The overexpressed HCV viral protein NS5A can interfere with the mitotic process and cause genomic instability. Interfering with these mitotic regulatory proteins can lead to chromosomal instability.^268^ HCV promotes genomic instability by activating activation-induced cytidine deaminase (AICDA), leading to DNA repair defects and an increased mutational phenotype in cellular genes, such as tumor suppressors or oncogenes, although no specific viral oncogenes have been identified.^271^ Peng et al. also found a strong association between methylation of the Ras association domain family protein 1 isoform A (RASSF1A) gene promoter and increased susceptibility to HCV-related HCC, highlighting RASSF1A methylation as a critical indicator of HCC tumorigenesis.^272^ Chronic HCV infection induces aberrant AICDA expression, which has been proposed to cause tumors by introducing translocations and somatic mutations into proto-oncogenes (Fig. 4f).^273^

*Interference cell proliferation, cell cycle, and apoptosis:* During HCV infection, viral proteins stimulate the expression of oncogenes, disrupt the cell cycle, and inactivate tumor suppressor genes, leading to the proliferation of quiescent hepatocytes.^257^ The HCV core protein and NS4B promote HCC development by directly activating the Wnt/β-catenin signaling pathway.^274^ Additionally, NS5A interacts with intracellular signaling pathways by inhibiting protein kinase R (PKR) activity and activating the PI3K/AKT pathway.^275^ HCV activates β-catenin, inducing the expression of oncogenes like c-Myc and cyclin D1, thereby accelerating the cell cycle and triggering a cascade of pro-carcinogenic events.^276,277^ The HCV core protein also modulates the expression of the cell cycle inhibitor p21, controlling unnecessary cell cycle progression and tumor formation.^278^ Furthermore, NS5A interacts with growth factor receptor-bound protein 2 (GRB2), p53, p21, and cyclins, inhibiting hepatocyte apoptosis.^279^ NS5A also activates PPARα and inhibits voltage-gated K^+^ channel Kv2.1 through ROS, further preventing hepatocyte apoptosis.^280^ Together, these mechanisms enable HCV to promote cell proliferation and transformation, ultimately leading to HCC development (Fig. 4f).

##### Indirect oncogenesis: promoting the progression of virus-related tumors

*Immunosuppression:* Innate immune sensors within hepatocytes, such as retinoic acid-inducible gene I protein (RIG-I), recognize HCV PAMP RNA and induce IFN-mediated local antiviral responses.^259^ However, HCV suppresses downstream IFN signaling and limits the expression of interferon-stimulated genes (ISGs).^281^ This persistent yet ineffective antiviral response within the liver microenvironment allows HCV to persist, leading to tissue damage and the development of HCC.^282^ Initially, HCV infection activates macrophages (Kupffer cells) in the liver and triggers the release of antiviral cytokines, such as IL-1β and IFN-γ.^283^ However, HCV core proteins and other inhibitory factors, such as PD-L1, suppress Kupffer cell function. During chronic HCV infection, the function and phenotype of NK cells are significantly altered, characterized by the downregulation of natural killer group 2 member D (NKG2D) expression.^284^ Additionally, specific HCV proteins can induce dysregulation of cellular surveillance, stem cell-like cell induction, and alterations in apoptotic signaling, becoming potential drivers of HCC.^285^ HCV proteins, including NS3A/4A, NS4B, and NS5A, activate TGF-β1 secretion through ROS and calcium-dependent mechanisms, thereby inhibiting immune responses.^286^ NS5A induces TGF-β1 secretion through interaction with TLR4, reducing NK cell cytotoxicity. NS5A also upregulates COX-2 expression, leading to the production of prostaglandins via the ERK/JNK signaling pathway and blocking IFN via the JAK/STAT pathway.^287^ Similarly, mucosal-associated invariant T cells (MAIT cells) are affected during HCV infection, exhibiting reduced numbers but an activated phenotype with impaired responsiveness to antigen stimulation.^288^ Additionally, the sustained stimulation of T cells by HCV antigens leads to T cell exhaustion, characterized by the upregulation of inhibitory receptors, such as PD-1, CTLA4, and TIM-3, resulting in the loss of T cell effector functions and reduced proliferative capacity.^289^ A large number of research have stated that HCV actively suppresses the immune response by altering the differentiation of innate immune cells, resulting in the impairment of the subsequent robust antiviral adaptive response.^290^ HCV infection can generate direct mechanisms to produce Tregs to suppress T cell responses. Patients with chronic infections also accumulate a large number of circulating Treg cells, which express CD45RO, high levels of intracellular CTLA-4 and PD-1, and have the ability to inhibit the proliferation of CD4^+^ and CD8^+^ T cells as well as the production of T helper 1 (Th1) cytokines.^291^ At the cytokine level, HCV can promote the production of a large number of immunosuppressive cytokines in the liver microenvironment, such as IL-10 and TGF-β. IL-10 can inhibit the activation and proliferation of T cells. By inhibiting the expression of co-stimulatory molecules on the surface of antigen-presenting cells (APCs), it reduces the activation signals received by T cells, thereby weakening the immune response of T cells.^292^ In addition, HCV infection is associated with the activation of inflammasomes, such as the release of NLRP3, ASC, caspase-1 and IL-1β secretion.^293^ Many of these cytokines and chemokines are important for the survival and differentiation of CD4^+^ T cells.^294^ The impairment of antiviral CD8^+^ and CD4^+^ Th1 T cell responses is associated with the persistence of HCV infection. These dysregulations and functional impairments of immune cells not only promote chronic liver damage and fibrosis but also ultimately contribute to the development of HCC (Fig. 4g).^295^ During HCV infection, the abundance of *Clostridiales* decreases, while the *Lactobacillus* and *Streptococcus* genera increase.^242^ Moreover, overgrowth and enrichment of *Streptococcus salivarius* have been observed. This bacterium is known to downregulate the immune response, especially in the context of advanced liver cirrhosis and HCC induced by HCV, indicating that it plays a role in the development and progression of HCC.^296^

*Chronic inflammation stimulation:* Chronic inflammation is a major driver of HCV-induced HCC. The immune system recognizes viral replication products, known as pathogen-associated molecular patterns (PAMPs) and damage-associated molecular patterns (DAMPs), which trigger the release of pro-inflammatory cytokines, such as IL-1α, IL-1β, and TNF-α. This leads to chronic liver inflammation and fibrosis.^160,297^ The HCV NS5A protein is an activator of the NF-κB pathway, which encodes several cytokines, including IL-6, TNF-α, and IL-1β.^298^ Additionally, HCV-infected cells secrete TGF-β1 following ER stress and ROS generation, which activates hepatic stellate cells and promotes fibrosis.^299^ While TGF-β1 initially inhibits tumor formation, it later promotes proliferation, angiogenesis and EMT, accelerating tumor progression. Oxidative damage exacerbates the inherent risk of mutations due to faulty DNA replication. This is primarily caused by ROS secreted by immune cells or released by mitochondria in infected hepatocytes.^300^ Furthermore, HCV induces ROS production as part of its replication strategy.^301^ The carcinogenesis resulting from HCV-induced chronic liver injury is a complex process, which is influenced by multiple factors.^262^ After HCV infects the liver, it will trigger a persistent inflammatory response. Inflammatory cells, such as macrophages and neutrophils, are recruited to the liver tissue and release a large number of inflammatory factors.^302^ Meanwhile, ROS generated in the chronic inflammatory environment will directly damage the DNA of hepatocytes, causing gene mutations or chromosomal abnormalities.^303^ Long-term HCV infection will also lead to liver fibrosis. Hepatic stellate cells are activated and proliferate excessively, secreting a large amount of extracellular matrix and destroying the normal structure and microenvironment of the liver.^304^ In the fibrotic liver, the intercellular signal transduction becomes disordered, and the interaction between hepatocytes and surrounding cells as well as the extracellular matrix is out of balance, further promoting the malignant transformation of cells (Fig. 4h).^305^

*Metabolic reprogramming:* HCV influences oncogenesis through the metabolic reprogramming of host cells. This reprogramming includes the regulation of energy metabolism pathways, lipid synthesis and metabolism, and alterations in cellular nutrient requirements. HCV promotes viral replication and survival through these mechanisms while driving tumor development in host hepatocytes.^200^ It has been reported that HCV induces the activation of inflammatory pathways like STAT3, which impairs peroxisome function by downregulating peroxisome proliferator-activated receptor α (PPAR-α), leading to the accumulation of long-chain fatty acids and potentially providing a replication advantage for the virus.^306^ The HCV protein NS5A interacts with glucokinase via its D2 domain (NS5A-D2), reprogramming central carbon metabolism to align with the energy and glycolytic phenotype required for HCV replication. Simultaneously, NS5A-D2 suppresses the interferon response triggered by HCV activation.^307^ HCV infection significantly impacts glucose metabolism, inducing aerobic glycolysis to stimulate anabolic pathways necessary for viral replication.^308^ Furthermore, HCV directly or indirectly interferes with insulin signaling, leading to systemic insulin resistance.^308^ Combined with lipid metabolism disorders and steatosis, the resulting hyperinsulinemia creates an anti-apoptotic, pro-proliferative environment in the liver that fosters tumor development (Fig. 4i).^309^

### Gastric cancer (GC)

**Fig. 5:**
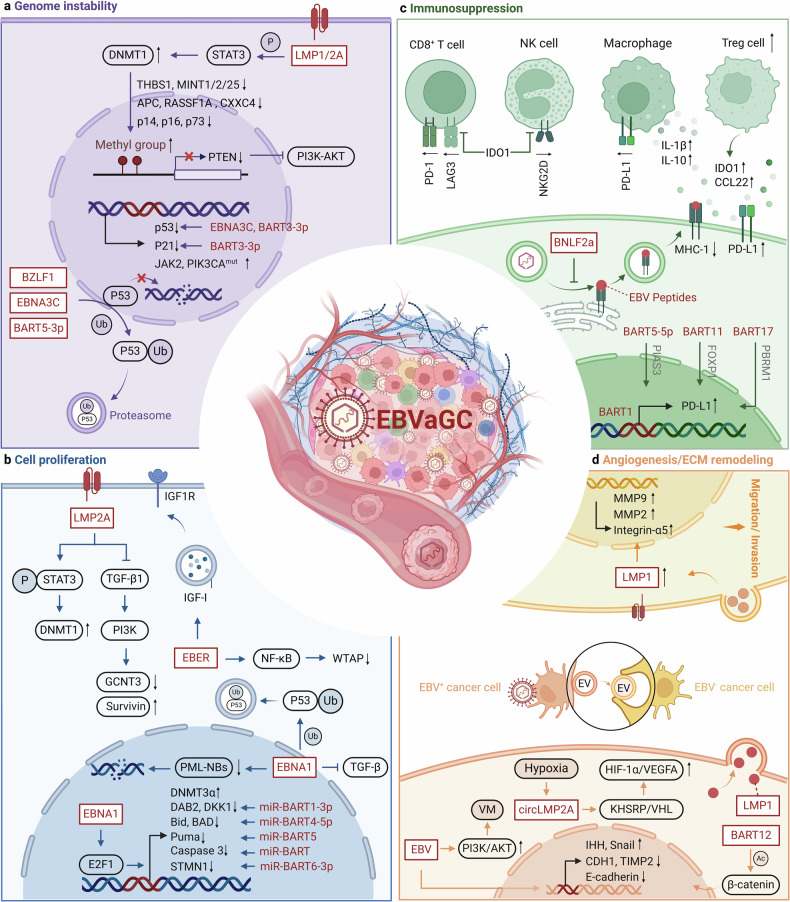
Oncogenic mechanisms of EBV in gastric cancer. **a** EBV induces genomic instability by promoting DNA methylation and inhibiting P53-mediated DNA repair. **b** EBV-encoded proteins disrupt the TGF-β1/Smad pathway, activate NF-κB and STAT3 signaling pathways, upregulate oncogene expression, and promote the degradation of tumor suppressor proteins, resulting in malignant cell proliferation. **c** EBV promotes immune evasion by impairing antigen presentation, increasing PD-L1 expression, and disrupting the function of CTLs and NK cells while increasing Tregs and TAMs in the TME. **d** EBV facilitates angiogenesis via KHSRP/VHL/HIF-1α/VEGFA and PI3K/AKT/mTOR/HIF-1α pathways and enhances extracellular matrix remodeling, aiding metastasis. Red text: EBV components; Black text: host cell components; Direct oncogenesis: **a**, **b**; Indirect oncogenesis: **c**, **d**. This figure was created with BioRender.com

EBV-associated gastric cancer (EBVaGC) accounts for 10% of all GC and is more common in males and younger individuals.^310^ Gastric cancer is a multi-step process that develops from chronic gastritis, atrophy, gastric intestinal metaplasia, and dysplasia, ultimately leading to gastric cancer. More than 95% of GC patients have a history of *Helicobacter pylori (H. pylori)* infection, which is a significant risk factor for non-EBV gastric cancer, although no correlation between H. pylori and EBVaGC has been found.^311^ EBV enters gastric epithelial cells via B lymphocytes, kills epithelial cells, and remains latent to promote cancer.^312^ In addition, elevated protein titers associated with EBV reactivation have been shown to precede the development of precancerous and malignant gastric lesions or to be associated with EBVaGC.^313^ EBV is often dormant in host cells but occasionally switches to the lysis cycle when stimulated; immunosuppressed state and other infections may trigger reactivation of EBV.^314^ The latent mode of EBVaGC corresponds to a unique latency I/II phase, characterized by the expression of EBV nuclear antigen 1 (EBNA1), non-coding RNAs (EBER1, EBER2), and EBV BamHI-A rightward transcripts (EBV-BART miRNA), typical of latency type I. Additionally, latent membrane protein 2A (LMP2A) is a feature of latency type II, expressed in approximately 50% of cases, while LMP-1 expression is rare.^315^ EBV latent gene products and EBV-encoded proteins may cooperate with the host genome to promote malignant transformation by regulating multiple signaling pathways, including cell cycle control, apoptosis, and tumor suppression (Fig. 5).^102,316^

#### Direct oncogenesis: leading to the emergence of virus-related tumors

##### Tumor suppressor gene inhibition

The p53 pathway is critical in the oncogenic process driven by EBV. Compared to EBV-negative GC, EBVaGC is characterized by a significant reduction in p53 expression, though mutations in p53 are rare.^315^ EBNA1 and EBNA3C suppress p53 transcription and promote its degradation. EBNA1 reduces p53 expression by targeting ubiquitin-specific protease 7 (USP7).^317^ The EBV-encoded immediate early protein—BamHI Z fragment leftward open reading frame 1 (BZLF1) also suppresses p53-dependent transcription by acting as a component of the ECS ubiquitin ligase complex (Elongin B/C-Cul2/5-SOCS-box proteins),^318,319^ facilitating p53 degradation.^320^ This direct inhibition of p53 by EBV may explain the low frequency of p53 gene mutations in EBVaGC.^321^

EBV is also the first human virus identified to encode multiple microRNAs.^322^ EBV-encoded EBV-miR-BART3-3p and EBV-miR-BART5-3p can bind to p53, reducing cellular senescence in EBVaGC, accelerating cell cycle progression, and preventing apoptosis.^323^ This suggests that EBV-encoded products also inhibit p53 function. It has been suggested that normal or elevated levels of p53 may confer a survival advantage to virus-infected cells, as these cells are less susceptible to apoptosis induced by JNK and rapamycin.^324^ Li et al. found that LMP1 induces hypermethylation of the H19 promoter, suppressing H19 and hsa-miR-675-5p expression, leading to overexpression of p53 protein in EBVaGC cells, which favors EBV latency.^325^ EBV-miR-BART9-5p maintains EBV latency by reducing the expression of the oncogene MUS81, promoting the progression of EBVaGC.^326^ Additionally, EBV-encoded EBV-miR-BART5-3p promotes p53 protein degradation, marking it as the first identified EBV-microRNA to inhibit p53 expression, highlighting EBV’s multiple strategies to maintain latency and promote EBV-related cancer development.^327^ Overall, p53 mutations do not play a major role in the early stages of carcinogenesis, while p53 reduction may promote tumor development as an independent event (Fig. 5a).

##### Genome instability

Upon entering gastric epithelial cells and establishing latency, EBV undergoes whole-genome methylation.^328^ Normal gastric tissue shows a promoter CpG island methylation level of 1-2%, while EBVaGC exhibits a CpG island methylation frequency of 19%.^329^ EBVaGC is typically characterized by somatic mutations in PIK3CA, loss of p16 (CDKN2A) expression, extensive DNA hypermethylation, and localized amplification of the 9p24.1 region, including JAK2, PD-L1, and PD-L2.^330,331^ In EBVaGC, abnormal DNA methylation of multiple genes or loci, including THBS1, APC, and p16, is widely observed.^332^ Other methylated genes include those involved in cell cycle regulation (p14ARF, p15, and p73), DNA repair (hMLH1, MGMT, and GSTP1), cell adhesion and metastasis (E-cadherin, TIMP1, and TIMP3), apoptosis (DAPK and Bcl-2), and signal transduction (APC, PTEN, and RASSF1A) (Fig. 5a).^333–335^

##### Interfering cell proliferation, cell cycle and apoptosis

EBV interferes with cell proliferation, cell cycle, and apoptosis in various ways during the occurrence and development of EBVaGC. EBV promotes the growth and survival of EBVaGC cells through its encoded proteins and small RNAs, such as EBNA1, LMP2A, and EBER.^336^ EBNA1 upregulates DNA methyltransferase 3a (DNMT3a) expression by activating the E2F1 transcription factor, thereby promoting gastric cancer cell proliferation, inducing cell cycle progression, inhibiting apoptosis, and accelerating migration in vitro.^337^ EBNA1 also causes the loss of promyelocytic leukemia (PML) nuclear bodies (NBs), weakening the DNA damage response and promoting gastric cancer cell survival.^338^ LMP2A activates DNMT1 transcription by phosphorylating STAT3, promoting EBVaGC cell growth.^339,340^ It also inhibits mTORC1 activation by inactivating the TGF-β1/Smad pathway and downregulating GCNT3 expression, thereby promoting cell proliferation and migration while preventing G0/G1 phase arrest.^341^ EBER induces insulin-like growth factor 1 (IGF-I) as an autocrine growth factor in EBVaGC, accelerating gastric cancer cell growth. EBER1 reduces the expression of the m6A “writer” Wilms’ tumor 1-associating protein (WTAP) by activating the NF-κB signaling pathway, further promoting the migration of EBVaGC cells.^342^

EBV-encoded miRNAs are divided into two main clusters, BamHI fragment H rightward open reading frame 1 (BHRF1) and BART miRNAs.^333,343^ In EBVaGC, the 44 mature miRNAs encoded by BART miRNA may interfere with genes related to cell death and cell cycle regulation.^333,344,345^ Specifically, EBV-miR-BART5-5p targets protein inhibitor of activated STAT 3 (PIAS3) and enhances PD-L1 expression, promoting gastric cancer cell proliferation, anti-apoptosis, invasion, and migration.^322^ EBV-miR-BART1-3p targets DAB2, promoting EBVaGC cell migration and reducing apoptosis.^346^ EBV-miR-BART6-3p regulates the expression of cell cycle-related proteins through the LOC553103-STMN1 axis, thereby inhibiting the proliferation of EBV-related tumor cells.^347^ EBV-miR-BART10-3p may promote EBVaGC proliferation and migration by targeting Dickkopf 1 (DKK1).^336^ EBV-miR-BART20-5p regulates cell proliferation and apoptosis by targeting Bcl-2-associated death promoter (BAD), promoting the development of EBVaGC.^348^ In short, EBV-encoded miRNAs play a significant regulatory role in the development of EBVaGC.

Like other cancers, EBV disrupts multiple cell cycle checkpoints, such as p16 and p21. p16 (CDKN2A) is a tumor suppressor gene that inhibits the cyclin D1/CDK4 complex, and it is commonly hypermethylated and silenced in EBVaGC.^349^ LMP1 also inactivates p16 by inducing the sequestration of E2F4 and E2F5 (E2F4/5) and the E26 transformation-specific transcription factor (Ets2) in the cytoplasm, leading to p16 dysfunction in these cells.^350,351^

EBNA1 promotes tumorigenesis by ubiquitinating p53, inhibiting TGF-β signaling, and enhancing the transcription of the anti-apoptotic protein survivin.^352^ Conversely, LMP2A can activate the PI3K/AKT proliferative pathway, increasing the survival of infected cells by upregulating survivin gene expression, inhibiting TGF-β1-induced apoptosis, and promoting cell migration through the Notch signaling pathway.^353,354^ EBV is known to encode over 40 different miRNAs. Among them, EBV-miR-BART1-3p directly targets disabled homolog 2 (DAB2), increasing the migration of EBVaGC cells and reducing apoptosis.^346^ EBV-miR-BART4-5p exerts anti-apoptotic effects in EBVaGC by modulating the expression of the BH3-interacting domain death agonist (Bid).^343^ EBV-miR-BART5 inhibits the expression of the pro-apoptotic Bcl-2 family member Puma, preventing Puma-mediated p53-independent apoptosis in EBVaGC cells.^355^ BART miRNAs also downregulate pro-apoptotic and anti-apoptotic mediators, such as caspase 3.^356^ In summary, EBV promotes the development and progression of EBVaGC by interfering with cell apoptosis through multiple mechanisms, including inhibiting p53 function, enhancing survivin expression, activating the PI3K/AKT pathway, and targeting apoptosis-related genes. These mechanisms interact with each other and jointly promote the occurrence and development of EBVaGC by interfering with cell proliferation, cell cycle, and apoptosis (Fig. 5b).

#### Indirect oncogenesis: promoting the progression of virus-related tumors

##### Immunosuppression and chronic inflammatory stimulation

EBV can persist and evade immune clearance, inducing an immunosuppressive tumor microenvironment (TME).^357,358^ EBVaGC is characterized by active immune infiltration, including CD8^+^ T cells, NK cells, macrophages, and Treg cells,^323,359^ along with elevated levels of IL-1β and IL-10, indicating a pro-inflammatory environment.^360^ The microenvironment also contains unique immune cell subsets, including highly proliferative T and B cells, B cells expressing T cell markers, and proliferative T cell clusters with follicular T helper cell markers.^361^ Antigen-specific ISG-15^+^CD8^+^ T cell populations are highly enriched in EBVaGC patients and exhibit a transitory exhaustion state.^362^ Due to this pro-inflammatory state, tumors show high PD-1, PD-L1, and LAG-3 expression.^359,363^ Additionally, elevated IFN-γ levels deplete tryptophan, inhibiting tryptophan-sensitive CTL and NK cells. Other molecules contributing to immunosuppression include CCL22, which increases Treg recruitment, and indoleamine 2,3-dioxygenase 1 (IDO1).^364^ Although the exact mechanism of PD-L1 overexpression in EBVaGC has not yet been clarified, it has been reported that EBV-miR-BART5-5p directly targets PIAS3 and controls and enhances PD-L1 through the EBV-miR-BART5/PIAS3/pSTAT3/PD-L1 axis, which contributes to anti-apoptosis, tumor cell proliferation, invasion, migration and immune escape.^322^ EBV-miR-BART11 and EBV-miR-BART17-3p inhibit FOXP1 and PBRM1 respectively, and enhance the transcription of PD-L1, facilitating tumor immune escape.^365^ Additionally, elevated IFN-γ levels deplete tryptophan, inhibiting tryptophan-sensitive CTL and NK cells.^364^ Other molecules that cause immune suppression include CCL22, which can increase Treg recruitment, and indoleamine 2,3-dioxygenase 1 (IDO1). The EBNA1 repeat sequence and the early lysis gene BNLF2A also inhibit antigen processing in EBVaGC, leading to immune evasion (Fig. 5c).^364,366^

The gut microbiota encompasses various pathogens, among which H. pylori is a notable example. This pathogen has the potential to induce epigenetic alterations and mutagenesis within the host genome. A significant majority of the bacteria implicated in the development of GC belong to the category of Gram-negative bacteria. Moreover, the co-infection scenario involving Gram-negative bacteria and viruses like EBV has been identified as a risk factor for GC.^367^ *Fusobacteria* are another kind of Gram-negative bacteria that are regarded as a risk factor for GC. GC, pancreatic, and colorectal cancers are often more abundant with *Fusobacterium* species, primarily *F. nucleatum*, than non-cancerous tissues.^368^

##### Extracellular matrix remodeling

LMP1-containing extracellular vesicles (EVs) enhance the adhesion, proliferation, migration, and invasion of recipient cells and remodel the TME by increasing the expression of ECM remodeling interaction proteins, such as matrix metalloproteinase 9 (MMP9), MMP2, and integrin-α5.^369^ LMP2A, BARF0, EBER, and EBNA1 affect cell behavior by downregulating key cell adhesion molecules, such as E-cadherin.^333,370^ Additionally, the absence of EBV-encoded EBV-miR-BART9 leads to elevated E-cadherin expression, reducing the proliferation and invasion potential of EBVaGC.^371^ Molecules important for cell invasion, such as THBS1, E-cadherin (CDH1), and TIMP2, also have their expression suppressed by promoter methylation, which may be related to the carcinogenesis process.^203,372^ In summary, EBV influences the invasion and growth potential of EBVaGC cells through multiple mechanisms, including ECM remodeling, downregulation of crucial adhesion molecules, and the expression of miRNAs (Fig. 5d).

##### Inducing angiogenesis

Angiogenesis is a key factor in tumor development and metastasis because the formation of new blood vessels enhances the delivery of nutrients and oxygen to rapidly proliferating cells.^373^ In EBVaGC, hypoxia-induced EBV-encoded circular RNA LMP2A (EBV-circLMP2A) promotes angiogenesis through the KHSRP/VHL/HIF-1α/VEGFA pathway.^373^ EBV-miRNA-BART12 inhibits tubulin polymerization-promoting protein 1 (TPPP1), suppressing α-tubulin acetylation, promoting microtubule dynamic assembly and acetylation, and activating EMT. These processes collectively enhance the invasion and metastasis of gastric cancer cells.^374^ Additionally, EBVaGC upregulates IHH (a gene that increases metastatic potential through angiogenesis) and Snail protein expression while reducing E-cadherin and tight junctions, further enhancing the invasive and metastatic capabilities of gastric cancer cells.^310,312^ EBV infection also induces the loss of PTEN expression through CpG island methylation of gene promoters, activating the PI3K-AKT signaling pathway.^340^ The loss of PTEN activates the PI3K/AKT pathway, leading to increased angiogenesis, cell migration, and loss of cell cycle adhesion.^375^ Moreover, EBV promotes the formation of vasculogenic mimicry through the PI3K/AKT/mTOR/HIF-1α pathway.^376^ In summary, EBV promotes gastric cancer cell growth, invasion, and metastasis by enhancing angiogenesis, thereby increasing the blood supply and metastatic potential of tumors (Fig. 5d).

### Cervical cancer (CC)

**Fig. 6:**
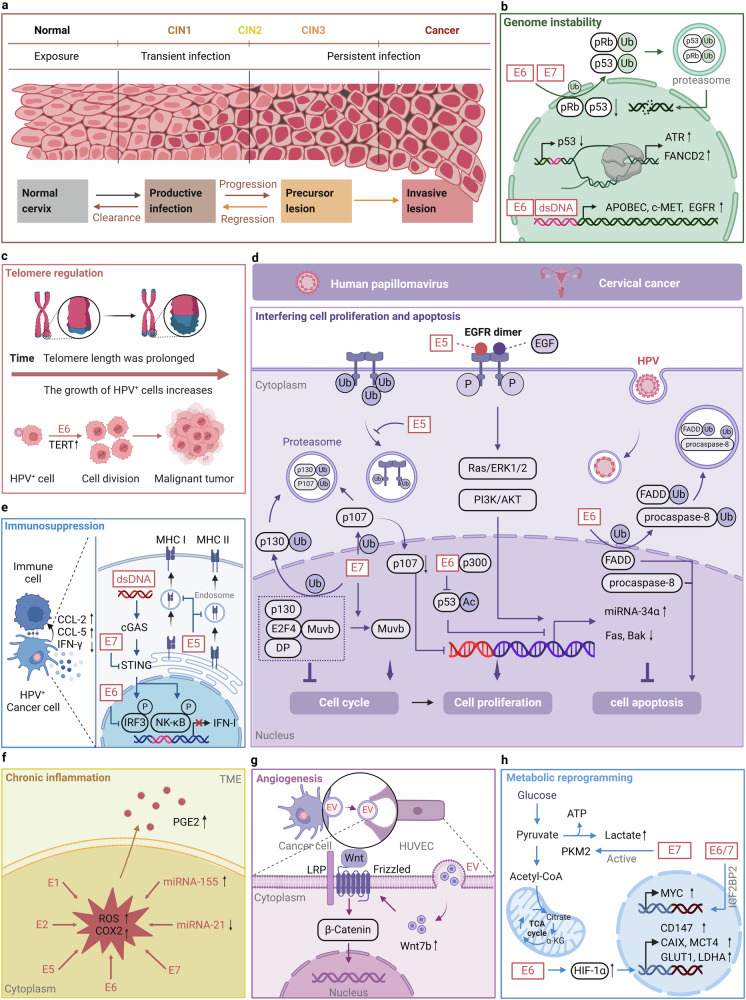
Oncogenic mechanisms of HPV in cervical cancer. **a** Progression of CC caused by HPV. **b** HPV infection disrupts DNA damage response and induces R-loop formation, leading to instability in DNA repair genes. **c** HPV promotes telomerase reverse transcriptase (TERT) accumulation, resulting in telomere elongation. **d** HPV-encoded proteins drive malignancy by activating EGFR signaling, degrading cell cycle inhibitors, and pro-apoptotic proteins. **e** HPV facilitates immune escape by inhibiting the cGAS-STING pathway, reducing antigen presentation, and inducing cytokine release. **f** HPV increases ROS and COX-2 production, causing chronic inflammation and oxidative stress. **g** HPV-positive cells can release extracellular vesicles (EVs) that promote angiogenesis and malignant transformation of epithelial cells. **h** HPV proteins drive cervical cancer aerobic glycolysis via upregulating HIF-1α/MYC transcription and activating PKM. Red text: Components of HPV; Black text: Components and biological processes of the host cell; Direct oncogenesis: **b**–**d**; Indirect oncogenesis: **e**–**h**. This figure was created with BioRender.com

HPV causes various malignancies in the anogenital and oropharyngeal regions.^377^ Most of HPV-associated cancers in women are cervical cancers (CC), whereas in men mostly HPV-associated cancer is head and neck squamous cell carcinoma (HNCC) related to HPV infection, which includes 30-60% of oropharyngeal carcinoma, 12% of pharyngeal cancer and 3% of oral cancer.^378^ It should be noted 90% of HPV infections get eliminated within a two-year period. But if infection endures may causes abnormal cells.^22^ The transformation into CC only takes place when these cervical cells traverse the basal membrane and infiltrate the tissues beneath (Fig. 6a).^22^

The occurrence of HPV-associated cancers is mainly caused by HPV viral proteins, such as E5, E6, E7. E5 primarily regulates cell growth and signaling pathways in HPV-infected cells;^379^ E6 promotes the ubiquitin-dependent degradation of p53, allowing the infected cells to evade apoptosis;^380^ and E7 disrupts cell cycle regulation, leading to cervical dysplasia.^381^ Notably, E6 and E7 are the principal oncogenic proteins expressed by both high-risk (HR) and low-risk (LR) HPV types. But only HR-HPV types encode the E5 protein, as LR-HPV lacks a defined homologous E5 ORF or the translation initiation codon for this protein.^382^ HPV16 and HPV18 are HR-HPV, but with distinctions. HPV16 is more prevalent and often associated with squamous cell carcinomas. It has a high replicative activity and is commonly found in a wide range of cervical cancers.^383^ In contrast, HPV18 is more likely to integrate into the host genome and is associated with adenocarcinomas.^384^

#### Direct oncogenesis: leading to the emergence of virus-related tumors

##### Oncogene activation and tumor suppressor gene inhibition

Both the HPV integration process and the E6 and E7 proteins can increase the risk of oncogene activation.^385^ The Apolipoprotein B mRNA editing enzyme catalytic polypeptide like (APOBEC) gene family, which belongs to the cytosine deaminase superfamily, is an oncogene that increases genomic instability when overexpressed.^386^ Study has shown that APOBEC3 mutations occur following the integration of high-risk HPV into the host cells, indicating that APOBEC is a molecular factor driving CC development by HPV.^387^ Additionally, HR-HPV E6 protein has been found to activate super-enhancers of oncogenes, such as EGFR and c-MET by reducing the stability of the histone demethylase KDM5C, thereby promoting oncogene activation.^388^

HPV-mediated cervical carcinogenesis hinges on the inactivation of two critical tumor suppressor genes: p53 and pRb.^389^ HPV E6 binds to p53 and recruits various ubiquitin ligases to facilitate p53 degradation.^390^ Research has found that the E6 protein directly binds to the short leucine consensus sequence of the ubiquitin ligase E6-associated protein (E6AP) to form an E6/E6AP heterodimer that ubiquitinates and degrades p53.^391^ HPV also promotes the nuclear export of human heterochromatin protein 1γ, which recruits UBE2L3 to target p53 for polyubiquitination and degradation.^392^ The LXCXE motif in the CR2 domain of HPV E7 binds to pRB and recruits cullin 2-containing E3 ubiquitin ligases to degrade pRB.^393^ On the whole, HPV E6 and E7 work together to inhibit the expression of tumor suppressor genes in host cells, leading to the oncogenic transformation of HPV-infected cells (Fig. 6b).

##### Genome instability and telomere maintenance

Whole-genome sequencing of HPV-associated cancer has revealed that HPV integration often leads to genome structural alterations, including duplications, deletions, translocations, and inversions.^394^ It has been found that after HPV integrates into host DNA may decrease the gene stability of p53,^395^ elevating the cancer risk. Moreover, HPV integration can hijack the DNA damage response (DDR) and utilize it to promote the malignant proliferation of host cells.^396^ Previous studies have indicated that E6 primarily induces DDR by promoting p53 degradation.^397^ R-loops are trimeric RNA consisting of an RNA-DNA hybrid and a displaced DNA strand.^398^ Recent studies have shown that HPV16 E6 induces abnormal R-loop accumulation by inhibiting the transcriptional activity of p53, leading to the instability of DNA repair genes, such as Fanconi anemia D2 protein (FANCD2) and ATR.^399^ E7 also induces DDR through multiple pathways, including binding to and inactivating pRb, interacting with the pRb-like protein CBP/p107,^397^ and directly interacting with DDR proteins to increase their activity,^400^ ultimately promoting DDR gene translation induced by E2F1 expression.^401^ In cervical epithelial cells co-infected with HPV and HIV, the frequency of catastrophic dysregulated alternative splicing is significantly higher than in cells infected with HPV or HIV alone, increasing the risk of malignancy (Fig. 6b).^402^

Telomeres are specialized DNA sequences at the ends of chromosomes that maintain chromosomal stability and naturally shorten with cell division, and are generally not or lowly expressed in keratinocytes.^403^ Abnormal accumulation or activation of TERT, leading to telomere elongation.^404^ Research also found that HPV-associated CC has higher TERT expression than HPV-infected NPE cells, suggesting that HPV interference with telomerase function is a potential mechanism of carcinogenesis.^405^ E6 typically extends telomeres through mechanisms including methylation of the TERT transcription start site,^406^ downregulation of the TERT transcription inhibitor NFX-1,^407^ and upregulation of the TERT transcription activator c-Myc.^408^ However, the effect of E7 on telomeres is complex, capable of inducing either telomere shortening or elongation depending on the context. In cell lines that over-express E7, TERT expression is low and telomeres are excessively shortened, suggesting that E7 can impair telomere length.^409^ Conversely, some studies have shown that E7 upregulates FANCD2, activating an ALT-dependent pathway to elongate telomeres (Fig. 6c).^410^

##### Interfering cell proliferation, cell cycle, and apoptosis

The oncogenic activity of E5 is mainly through hyperactivation of the EGFR.^379,411^ EGFR is a known stimulator of cell growth and contributes to tumor progression.^412^ E5 interacts with the 16-kDa subunit of vacuolar ATPase, disrupting endosomal acidification, which promotes EGFR recycling at the cell membrane and enhances its mitogenic activity.^413^ Moreover, EGFR is degraded by the E3 ubiquitin ligase c-Cbl protein, but such activity is inhibited by HPV16 E5, reducing EGFR degradation.^414^ Downregulation of pRB, p107, and p130 by the viral E7 protein contributes to the uncontrolled proliferation and malignant transformation of HPV-infected cervical epithelial cells.^415^ TGF-β significantly suppresses epithelial cell proliferation. Studies have shown that HPV-encoded viral proteins bind to TGF-β receptors and signaling sensors, leading to their degradation and promoting the proliferation of infected cells.^416^ Similarly, the hedgehog pathway activated by HPV16 E6 and E7 may transform esophageal basal cells and promote esophageal squamous cell carcinoma.^417^

The DREAM repressor complex inhibits the transcription of genes associated with cell division.^418^ HPV E7 primarily interferes with the cell cycle by modulating the DREAM complex.^419^ On one hand, HPV E7 directly interacts with the B-Myb-MuvB complex to promote the transcriptional activation of genes associated with the S and M phases of the cell cycle.^420^ On the other hand, HPV E7 activates inhibitory proteins of DREAM, such as cyclin-dependent kinase inhibitors p21 and p27, thereby reducing the activity of the DREAM complex.^421^ Additionally, HPV E7 can upregulate the degradation of DREAM activators p107 and p130.^422^ Moreover, by modulating CDK inhibitors, such as p21CIP1 and p27KIP1, E7 disrupts the G1/S checkpoint.^423^ Unlike HPV E7, HPV E6 primarily overcomes cell cycle checkpoints by inactivating p53, thereby loosening cell cycle regulation.^424^ The E6-AP-E6-p53 complex downregulates hsa-miR-34a expression, leading to upregulation of p18Ink4c and driving cells into the S phase.^425^ In addition, E6 interacts with Aurora kinase A to regulate cyclin E and phosphorylated histone H3 expression, accelerating G1/S transition and mitosis.^426^

HPV proteins E5 and E6 evade apoptosis through various mechanisms. For example, E5 downregulates Fas expression,^427^ activates the Ras-Raf-MAP kinase pathway and the PI3K-Akt pathway,^428^ and degrades the pro-apoptotic protein Bax to escape apoptotic signals.^429^ In contrast, E6 reduces Bak expressionand degrades FADD and procaspase-8 to inhibit apoptosis.^430^ By contrast, HPV E2 and E7 are primarily involved in inducing host cell apoptosis,^431^ suggesting that HPV induction of apoptosis escape in host cells is a complex, dynamic process influenced by multiple factors. HIV Tat protein are capable of upregulating the expression of the HPV E6 protein. This process inhibits the expression of p53, thereby conferring the potential for immortality upon the infected cells (Fig. 6d).^432^

#### Indirect oncogenesis: promoting the progression of virus-related tumors

##### Immunosuppression

In CC, HPV E6 and E7 produce a sustained immunosuppressive state by inhibiting the cGAS-STING signaling pathway, reducing HPV-associated antigen presentation, decreasing immune cell recruitment, and suppressing cytokine release.^433^ HPV18 and 16 E7 proteins inhibit STING through the LCXCE sequence and NLRX1, respectively.^434^ Additionally, HPV16 E6 can inhibit IFN regulatory transcription factor IRF3, reducing the activation of the cGAS-STING signaling pathway.^435^ Large-scale genome-wide association studies (GWAS) have found that the HPV genome frequently integrates near the MHC-I polypeptide-related sequence A, leading to an increase in MHC-I gene mutations, which is a potential mechanism of HPV immune evasion.^436^ Furthermore, the disruption of chemokine regulation caused by HPV E6 and E7,^437^ such as the upregulation of CCL-2 and CCL-5 and the downregulation of IFN-γ,^438^ reduces immune cell recruitment and promotes an immunosuppressive microenvironment at the site of infection.^439^

In CC, HPV E5 protein directly interferes with the translation, transport, and degradation of MHC I, preventing the activation of adaptive immunity.^440^ In addition to affecting MHC-I expression, E5 also directly reduces the expression of MHC-II complexes,^441^ calnexin, and CD1 receptors that activate immune cells, establishing a sustained immunosuppressive state.^87,442^

The normal vaginal microbiota (VMB) is dominated by Lactobacillus, which can maintain an acidic environment and produce antibacterial proteins to resist pathogens.^377^ Many studies have shown that the imbalance of vaginal microbiota is closely related to the development of cervical intraepithelial lesions and CC.^443^ In relevant studies, *L. crispatus*, *E. eligen*, *G. vaginalis*, *U. urealyticum*, *P. stutzeri* and *A. vaginae* have been identified as high-risk factors for HPV-CC, while high abundances of D. invisus, *F. magna*, *G. vaginalis*, *P. buccalis*, *P. timonensis* and *L. johnsonii* significantly reduce the risk of HPV-CC.^444^ Specifically, high abundances of *Prevotella* can affect the occurrence of cervical lesions related to persistent HR-HPV infection through the host NF-kB and C-myc signals,^445^ and *M. microronuciformis* mainly induces or maintains the production of viral oncoproteins E6 and E7 to promote cancer.^446^ In addition, studies have also shown that the imbalance of VMB is closely related to the expression levels of immune checkpoint molecules in HPV-CC. PD-L1 and LAG-3 are negatively correlated with *Lactobacillus* species and positively correlated with dysbiotic bacteria, whereas TLR2 is negatively correlated with dysbiotic bacteria and positively correlated with lactobacilli.^447^

Overall, in cervical cancer, HPV E6 and E7 mainly influence the cGAS-STING signal, and E5 interferes with the expression of MHC. However, the impact of HPV on the immune microenvironment of head and neck cancers differs from that in CC. Firstly, in HNCC, it is E5, rather than E6 and E7, that suppresses immunity by inhibiting the immunoproteasome and STING pathway.^413,448^ Secondly, E6 and E7 no longer play an immunosuppressive role but function as potent immunostimulants instead. They can effectively trigger specific T-cell responses and enhance the anti-tumor activity within the microenvironment of HNCC.^449^ This unique mechanism results in differences in clinical prognosis. It has been reported that compared with non-HPV-infected CC, the prognosis of HPV-infected CC is poorer. Nevertheless, the prognosis of HPV-infected OPSCC is better than that of non-HPV-infected OPSCC (Fig. 6e).^450^

##### Chronic inflammation stimulation

The persistent presence of chronic inflammation increases the risk of malignant cellular transformation.^451^ HPV proteins E6, E7, and their miRNAs can promote the continuous release of inflammatory mediators directly through signaling pathways, such as STAT3, NF-κB, and EGFR, including COX-2 and its metabolites like PGE2.^452^ HPV proteins E5, E6, and E7 all activate the EGFR-Ras-MAPK-AP-1 pathway, increasing COX-2 transcription and maintaining the body in a state of chronic inflammation.^453^ This process is a positive feedback loop where COX-2 is continuously expressed.^453^ Following HPV infection, the host and microenvironment’s immune cells experience miRNA dysregulation, such as the silencing of hsa-miR-21 or the overexpression of hsa-miR-155, which contributes to the establishment of a chronic inflammatory state.^454^ The chronic inflammation induced by HPV infection also leads to the production and release of ROS and reactive nitrogen species in HPV-infected cells.^455^ Compared to non-HPV-infected cervical epithelial cells, HPV-induced intraepithelial neoplasia and CC exhibit significantly increased oxidative stress products. These oxidative stresses are considered direct consequences of HPV proteins. For example, co-expression of E1 and E2 increases ROS levels and γH2AX deposition, and ROS levels also increase in HPV-negative cervical epithelial cells transfected with E6 alone.^456^ In short, HPV-induced chronic inflammation and oxidative stress accelerate the malignant transformation of epithelial cells. HIV infected T cells contain hsa-miR-155-5p, which can be internalized by cervical epithelial cells and activate the ERCC5-NF-κB signaling pathway, leading to the secretion of pro-inflammatory cytokines IL-6 and IL-8. This enhances the invasive capacity of cervical epithelial cells (Fig. 6f).^457^

##### Inducing angiogenesis

EVs are mediators of communication between cancer cells and the TME and are potential factors in HPV-induced carcinogenesis.^458^ HPV16 and 18 E6 upregulate Wnt7b mRNA expression in host cell lines and host-derived EVs. These Wnt7b mRNA-rich EVs subsequently activate β-catenin in HUVE cells, ultimately promoting angiogenesis and malignant transformation of epithelial cells.^459^ Additionally, HPV-induced circRNA abnormalities contribute to the progression of CC by MAPK, and PI3K/AKT/mTOR signaling pathways.^460^ HIV also leads to an upregulation of neurotrophic factors BDNF and NGF, increasing local nerve infiltration, vascular invasion, and lymph node metastasis, ultimately inducing the occurrence of CC (Fig. 6g).^461^

##### Metabolic reprogramming

HPV can also promote the metabolic reprogramming of host cells.^462^ In HPV-infected CC, HPV E6 can increase the expression and stability of HIF-1α and upregulate the expression of HIF-1α target genes, such as GLUT1, LDHA, CAIX, MCT4 and CD147, thus promoting the Warburg effect.^463^ Meanwhile, the HR-HPV E7 protein can activate the glycolytic enzyme pyruvate kinase M2 (PKM2) to induce the Warburg effect.^464^ In addition, HPV E6 and E7 proteins can activate IGF2BP2 to stabilize m6A-MYC, which can promote MYC mRNA transcription and then drive aerobic glycolysis in cervical cancer.^465^ Similarly, in HPV-infected HNSCC, HR-HPV E6 proteins activate the HIF-1α and Akt/mTOR signaling pathways, and HR-HPV E6 activates PKM2 and COX, ultimately promoting glycolysis (Fig. 6h).^466^

### Nasopharyngeal carcinoma (NPC)

**Fig. 7:**
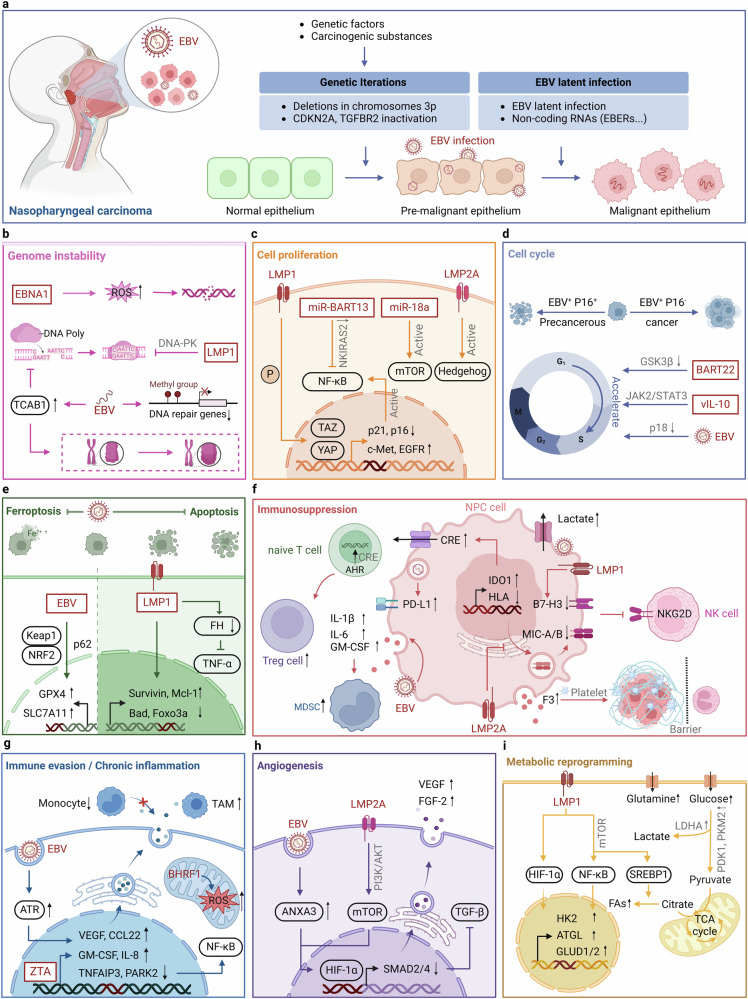
Oncogenic mechanisms of EBV in nasopharyngeal carcinoma. **a** Progression of NPC caused by EBV. **b** EBV induces genetic instability through inhibition of DNA repair, increased DNA methylation, chromosomal breaks at 11q23, and telomere elongation. **c** EBV promotes cell proliferation by activating NF-κB, Hedgehog, mTOR signaling pathways. **d** EBV accelerates G1/S progression in host cells by downregulating GSK3β expression, promoting degradation of p18, and activating the JAK2/STAT3 signaling pathway. **e** EBV infection promotes anti-apoptotic protein expression, inactivates pro-apoptotic proteins, reduces fumarate hydratase (FH) activity, and inhibits ferroptosis through the p62-Keap1-NRF2 pathway. **f** EBV evades immune surveillance by modulating PD-L1, MIC-A/B, and B7-H3 expression, and induces lactate, creatinine, and cytokine release, which recruits Treg cells and expands myeloid-derived suppressor cells (MDSCs), creating an immunosuppressive microenvironment. **g** EBV proteins promote cytokine/chemokine release, inhibit monocyte recruitment, induce TAM, and enhance mitochondrial activity via ROS suppression. **h** EBV-encoded proteins activate ANXA3-HIF-1α-VEGF and PI3K/AKT/mTOR/HIF-1α pathways to promote angiogenesis. **i** EBV stimulates glycolysis, increases glutamine uptake, and inhibits lipid degradation via the mTORC1/NF-κB and HIF-1α pathways. Red text: EBV components; Black text: host cell components; Direct oncogenesis: **b**–**e**; Indirect oncogenesis: **f**–**i**. This figure was created with BioRender.com

The establishment of type II latency is the initial step in EBV-driven epithelial malignancies. In nasopharyngeal epithelial (NPE) cells, EBV typically exists in a lytic state, while in precancerous lesions and nasopharyngeal carcinoma (NPC), EBV is in type II latency. This suggests that type II latent EBV is closely associated with NPC development.^467^ Type II latent EBV mainly expresses non-coding RNAs (EBER), Epstein-Barr nuclear antigen 1 (EBNA1), latent membrane proteins (LMPs), and BamHI A transcripts (EBV-miR-BARTs).^310^ Constitutively expressed latent proteins and EBV-miR-BARTs, which contribute to the evasion of NPE cell death, suppression of host immune responses, and genomic instability.^468^ Meanwhile, heterogeneously expressed LMPs drive tumor progression in invasive epithelial cancers through metabolic reprogramming and inducing NPE cell dedifferentiation.^469^ Notably, although type II latency EBV induces some oncogenic features and abnormal signaling pathways, it still requires some additional acquired genetic alterations for the malignant transformation (Fig. 7a).^470^

#### Direct oncogenesis: leading to the emergence of virus-related tumors

##### Genome instability

EBV can both induce and counteract host genome instability. The genetic vulnerability caused by EBV integration mainly depends on the activation of fibroblast growth factor (FGF) and NF-κB signaling pathways.^471^ LMP1 protein inhibits the activity of DNA-dependent protein kinase, reducing the repair of double-strand breaks (DSBs).^472^ EBV infection often leads to hypermethylation of promoter regions of genes related to DNA repair, significantly downregulating their transcription, and in vitro experiments have confirmed that DNA repair capacity is diminished in EBV infected NPE cells.^473^ Additionally, the sequence-specific DNA-binding domain of EBNA1 can bind to EBV like palindromic sequences on human chromosome 11q23, which may trigger chromosome breakage.^474^ However, some reports suggest that EBV infection can enhance DNA repair capacity, particularly after radiotherapy and chemotherapy. The EBNA1 protein promotes DNA damage in NPE cells by inducing ROS and upregulating oxidative stress response proteins, such as SOD1 and Prx1.^475^ TCAB1 is involved in telomere elongation and ribonucleoprotein biogenesis, thus plays a role in activating telomerase and repairing DNA damage.^476^ While numerous studies have revealed various DNA repair mechanisms in NPC, the relationship between these DNA repair processes and EBV remains unclear and requires further investigation (Fig. 7b).^477^

##### Interfering cell proliferation, cell cycle, and apoptosis

EBV promotes malignant cell proliferation primarily through the activation of the NF-κB complex.^478^ The activation of the NF-κB signaling pathway by the LMP1 protein is a major cause of malignant proliferation in NPE cells.^479^ Mechanistically, LMP1 induces increased activity of Yes-associated protein (YAP) and transcriptional coactivator with PDZ-binding motif TAZ, upregulates the transcription levels of EGFR and c-Met, and downregulates cell cycle inhibitors p16 and p21, thereby activating the NF-κB pathway and promoting malignant proliferation in NPE cells.^480^ Additionally, EBV maintains NPE cell stemness by promoting Hedgehog autocrine signaling.^481^ Furthermore, EBV-miR-BART13 interacts with NF-κB inhibitor-interacting Ras-like 2 (NKIRAS2) to inhibit the activation of NF-κB signaling, inducing NPE cell proliferation.^482^ EBV-miR-18a can upregulate SMG1 and activate the mTOR signaling pathway, further enhancing the proliferative capacity of NPE cells (Fig. 7c).^483^

The regulatory effect of EBV on the cell cycle of NPE cells depends on whether the NPE cells have acquired additional abnormal genetic changes, such as p16 deletion. After the NPE cells acquire abnormal genetic changes, the products encoded by EBV accelerate the G1/S progression by regulating cell cycle checkpoints and related regulatory factors, further promoting the progression of NPC.^484^ EBV-encoded vIL-10 can activate the JAK2/STAT3 signaling pathway, increasing IL-6 expression and promoting G1/S phase progression.^485^ EBV-miR-BART22 upregulates hsa-miR-4721 transcription, leading to decreased GSK3β expression, thereby increasing nuclear accumulation of β-catenin and activating CCND1 and c-Myc.^486^ EBV infection can also downregulate the deubiquitinating enzyme cylindromatosis, leading to increased degradation of the cell cycle negative regulator p18, accelerating the G1/S phase transition.^487^ However, some EBV-derived products inhibit NPE cell proliferation. For instance, EBV-miR-BART6-3p negatively regulates the LOC553103/STMN1 axis, downregulating cyclin proteins (like CCNE1, CCND1, and CDK4) and causing cell cycle arrest in EBV-associated tumor cells (Fig. 7d).^347^

A large number of miR-BARTs are present in epithelial cells infected by EBV. These abundantly expressed miR-BARTs promote the occurrence of NPC by inhibiting apoptosis.^488^ Beyond EBV-miR-BARTs, LMP1 also demonstrates anti-apoptotic activity by promoting the expression of anti-apoptotic proteins (like survivin and Mcl-1) and inactivating pro-apoptotic proteins (like Bad and Foxo3a).^469,489^ LMP1 can decrease fumarate hydratase activity, protecting NPC cells from TNF-α-induced necroptosis in the microenvironment.^490^ EBV also reduces the sensitivity of NPC cells to ferroptosis by activating the p62-Keap1-NRF2 signaling pathway and upregulating SLC7A11 and GPX4, thereby ensuring NPC survival under high oxidative stress and promoting the development of NPC.^491^ Furthermore, EBV can upregulate transferrin receptor through unknown pathways, which inhibits apoptosis via activation of the PI3K/AKT/mTOR signaling axis (Fig. 7e).^492^

#### Indirect oncogenesis: promoting the progression of virus-related tumors

##### Immunosuppression

EBV contributes to immune evasion in NPC cells by upregulating inhibitory receptors and downregulating MHC class I and II antigens. EBV primarily promotes immune evasion in NPC by upregulating PD-L1. EBER1,^493^ LMP1,^494^ and circBART2.2 can activate the RIG-I pathway to upregulate PD-L1 transcription.^495^ EBV-miR-BART11 and EBV-miR-BART17-3p inhibit FOXP1 and PBRM1, respectively, enhancing PD-L1 transcription.^365^ Additionally, EBV increases DNA methylation, which constitutively activates NF-κB signaling and promotes the activation of genes associated with T cell exhaustion, such as CD74.^496^ EBV induces the expression of inhibitory receptors on NK cells, such as TIGIT, LAG3, and ZNF683.^497^ Furthermore, LMP1’s activation of the PI3K/AKT/mTOR signaling pathway increases B7-H3 expression, inhibiting NK cell cytotoxicity.^498^ Research also shows that EBV upregulates F3, leading to the accumulation of activated platelets, forming a physical barrier that prevents NK cells from entering the TME (Fig. 7f).^357^

##### Chronic inflammation stimulation

EBV integration downregulates the expression of inflammation-related genes, such as TNFAIP3, PARK2, and CDK15, activating the TNF-α-induced apoptosis and NF-κB pathway, which is closely associated with NPC development.^499^ The EBV-encoded transcriptional activator ZTA upregulates inflammatory cytokines GM-CSF, IL-8, and GRO-α, which suppresses monocyte chemotaxis and induces tumor-associated macrophage phenotypes.^500^ Additionally, EBV activates the ATR and Rad-3-related pathway, promoting PPAR-δ and inhibiting c-Jun and p-JNK expression. This leads to increased expression of inflammatory factors VEGF and CCL22, polarizing TAMs toward an M2 phenotype and accelerating malignant transformation in NPE cells.^501^ EBV-encoded BHRF1 protein localizes to mitochondria, causing mitochondrial membrane permeabilization transition (MMPT), increasing ROS production, and enhancing mitochondrial activation, thereby promoting tumorigenesis (Fig. 7g).^502^

##### Vascular and extracellular matrix remodeling

EBV supports NPC cell progression through the enrichment of vascular networks.^316^ LMP2A protein activates the PI3K/AKT/mTOR/HIF-1α signaling promoting the growth of tumor vascular networks.^373^ Moreover, EBV stimulates vasculogenic mimicry (VM) through various pathways,^376,503^ such as activation of the PI3K/AKT/mTOR/HIF-1α pathway.^376,504^ Interaction between LMP1 and TNF-α activates the EGFR/Src/ERK/cortactin and Cdc42/N-WASP signaling axis, mobilizing invadopodia to overcome matrix barriers.^505^ Additionally, exosomes from EBV-infected cells are rich in HMGA2, which upregulates Snail expression, promoting endothelial-to-mesenchymal transition (EndMT) in endothelial cells, thereby enhancing tumor metastasis (Fig. 7h).^506^

##### Metabolic reprogramming

EBV is responsible for the promotion of NPC through the increase in glycolysis. Initially, the LMP1 protein can upregulate HIF-1α expression,^507^ which in turn activates the mTORC1/NF-κB signaling pathway. This then leads to the upregulation of GLUT1 transcription, which enhances glucose uptake in epithelial cells.^508^ Additionally, LMP1 enhances HK2 activity and upregulates the expression of glycolytic enzymes, such as pyruvate dehydrogenase kinase 1 (PDK1) and pyruvate kinase M2 (PKM2).^509^ Besides LMP1, EBV-miR-BART1-5P also activates mTOR and HIF-1α, further increasing glycolysis in host cells.^510^ Given that the energy generated from glycolysis is insufficient to meet the demands of rapidly dividing cancer cells, EBV also increases glutamine uptake to sustain growth and metabolic needs,^511^ upregulating mitochondrial glutamate dehydrogenase enzymes GLUD1 and GLUD2 to catalyze the conversion of glutamine into α-KG.^512^ EBV miRNAs and proteins stimulate increased lipase activity in adipocytes and enhance the AMPK metabolic pathway, promoting lipolysis to provide energy (Fig. 7i).^513^

### Kaposi sarcoma (KS)

**Fig. 8:**
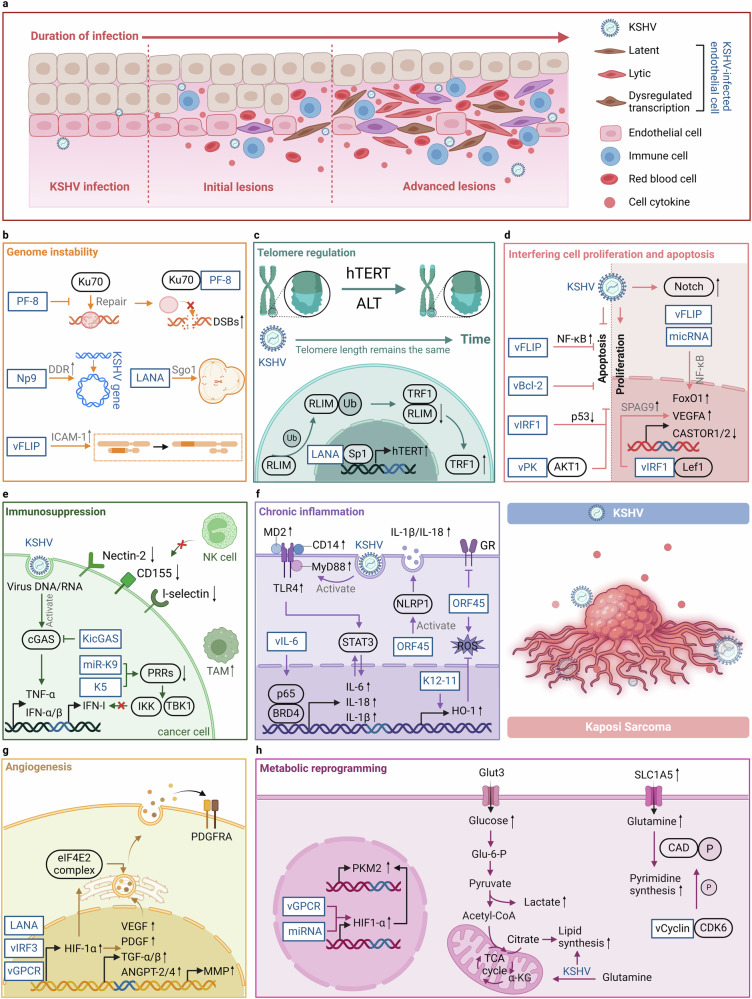
Oncogenic mechanisms of KSHV in Kaposi sarcoma. **a** Progression of KS caused by KSHV. **b** KSHV infection induces intracellular DNA damage, mitotic disruption, and chromosomal structural abnormalities. **c** KSHV maintains telomere length in host cells by reducing TRF1 degradation. **d** KSHV enhances cell proliferation via NF-κB and Notch signaling, while vBcl-2 and vPK inhibit apoptosis. **e** KSHV facilitates immune evasion by inhibiting cGAS activity, suppressing IFN-I transcription, downregulating Nectin-2, CD155, and I-selectin ligands, and fostering a Treg and TAM-rich immunosuppressive microenvironment. **f** KSHV induces chronic inflammation and oxidative stress via promoting inflammatory factor release, inhibiting GR signals, and upregulating HO-1 transcription. **g** KSHV promotes angiogenesis and metastasis by increasing angiogenic factors and matrix metalloproteinases (MMPs). **h** KSHV infection augments aerobic glycolysis, pyrimidine synthesis, amino acid metabolism, and lipid generation in host cells. Blue text: Components of KSHV; Black text: Components of host cell; Direct oncogenesis: **b**–**d**; Indirect oncogenesis: **e**–**h**. This figure was created with BioRender.com

Kaposi sarcoma (KS) is characterized by abnormal angiogenesis, inflammation, and proliferation, with Kaposi sarcoma-associated herpesvirus (KSHV) being the primary cause of KS development.^514^ In host cells, KSHV typically persists as a latent infection, encoding latent-associated nuclear antigen (LANA), vCyclin, vFLIP, and KS miRNAs during latency. These transcripts stimulate cell growth and proliferation, promote angiogenesis, and activate inflammatory signals.^515^ Additionally, KSHV can transition to lytic replication upon activation by various biological signals, producing lytic replication products, such as K1, viral interferon regulatory factors (VIRFs), vIL-6, vCCLs, vGPCR, and K15, which regulate host cell signaling pathways and accelerate KS progression (Fig. 8a).

#### Direct oncogenesis: leading to the emergence of virus-related tumors

##### Genome instability and telomere maintenance

KSHV-encoded viral proteins induce the DDR and chromosomal instability, leading to genomic instability.^516^ The Np9 protein directly causes DDR or indirectly induces DDR by upregulating LANA and c-Myc.^517^ LANA protein interferes with the localization of Sgo1 to the centromere, leading to the degradation of Securin and Cyclin B1, which significantly increases chromosomal instability.^518^ vFLIP activates the NF-κB pathway, enhancing L1 retrotransposition, with ICAM-1 involved in regulating this process, potentially accelerating the development of KS (Fig. 8b).^516^

To maintain unlimited proliferative capacity, cancer cells must activate telomere maintenance mechanisms to prevent telomere shortening.^403,519^ KSHV-transformed cells utilize two mechanisms to maintain telomere length. Recent studies have also found that KSHV induces alternative lengthening of telomeres activity (ATL) in host cells, enhancing cell proliferation, but the specific mechanism is not yet clear.^109,520^ Collectively, these processes highlight KSHV's dual role in promoting genomic instability and sustaining telomere integrity to drive Kaposi sarcoma pathogenesis (Fig. 8c).

##### Interfering cell proliferation and apoptosis

KSHV-miR-K4-5p and KSHV-miR-K1-5p activate the mTORC1 pathway by downregulating cytosolic arginine sensor for mTORC1 subunits 1 and 2, thereby promoting cell proliferation and malignant transformation.^521^ Moreover, KSHV miRNAs and vFLIP upregulate forkhead box protein O1 (FoxO1) by activating the NF-κB pathway, further promoting cell proliferation.^522^ KSHV infection also increases the expression of Notch signaling pathway proteins, including Notch1, NICD, RBP-Jĸ, and Hes1, further promoting cell proliferation.^523^ Additionally, vIRF1 upregulates SPAG9, enhancing the interaction between MKK4 and JNK1/2, leading to increased VEGFA expression and cell proliferation.^524^ KSHV regulates the apoptosis signaling pathways through various viral-encoded proteins to enhance the survival of infected cells. Specifically, ORF 16 encodes vBcl-2, a viral homolog of the human proto-oncogene Bcl-2, with similar anti-apoptotic functions.^525^ K15 interacts with the anti-apoptotic protein HAX1 and induces the expression of multiple anti-apoptotic genes, providing a survival advantage.^526^ Additionally, KSHV ORF36 encodes viral protein kinase (vPK), which binds to AKT1 through its PH domain, upregulating AKT1 activity and inhibiting apoptosis, thus conferring a survival advantage to endothelial cells (Fig. 8d).^523^

#### Indirect oncogenesis: promoting the progression of virus-related tumors

##### Immunosuppression

KSHV suppresses the innate immune responses through multiple mechanisms. The KicGAS protein, produced by ORF52, directly inhibits the enzymatic activity of cGAS, interfering with DNA-induced phase separation and thereby suppressing the host’s antimicrobial and antitumor responses.^527^ Additionally, KSHV encodes ubiquitin ligases K3 and K5, which reduce the expression of antigen-presenting molecules and NK cell recognition ligands in host cells,^528^ including the downregulation of Nectin-2, CD155, and I-selectin.^529^ The evasion of adaptive immunity by KSHV principally hinges on the aberrant secretion of cytokines,^530^ such as those that induce a hyperinflammatory state and increase the infiltration of tumor-associated macrophages.^531^ In addition, the majority of KS patients are co-infected with HIV and KSHV. The immunodeficiency resulting from HIV constitutes a significant backdrop for the occurrence of KS (Fig. 8e).^532^

##### Chronic inflammation stimulation

Hyperinflammation is a hallmark of KS.^533^ KSHV infection drives the abnormal activation of various pro-inflammatory pathways. LANA can increase the activation of TLR4 and its adapters, activating the STAT3 pathway, which is considered a key viral protein responsible for the sustained release of pro-inflammatory factors IL-6, IL-1β, and IL-18.^534^ Additionally, elevated vIL-6 enhances the recruitment of BRD4 and the persistent binding of NF-κB p65, leading to the increased release of inflammatory factors.^535^ KSHV also encodes ORF45, which dissociates the Linker1-UPA complex, releasing the C-terminal domain of hNLRP1, thereby activating the NLRP1 inflammasome and promoting the maturation and release of IL-1β and IL-18, exacerbating inflammation.^536,537^ Studies have shown that KSHV miRNAs and vFLIP can activate the NF-κB pathway and upregulate FoxO1, accelerating the clearance of ROS within cells.^522^ Additionally, KSHV-miR-K12-11 targets BACH1 mRNA, reducing its translation, thereby increasing the transcription of the antioxidant enzyme heme oxygenase-1 and activating the NRF2-dependent antioxidant stress pathway (Fig. 8f).^538^

##### Inducing angiogenesis

KS is a highly angiogenic endothelial cell tumor, and KSHV-induced angiogenesis plays a crucial role in KS development.^539^ KSHV primarily promotes angiogenesis by upregulating HIF, VEGF, and their receptors, thereby activating angiogenesis-related pathways.^540^ KSHV encoded LANA,^541^ vIRF3,^524,542^ vIL-6,^535,543^, and vGPCR increase angiogenic factors,^544^ including VEGF, PDGF, TGF-α, TGF-β, ANGPT-2, and ANGPTL-4, in an mTOR-dependent manner. KSHV-encoded ORF57 can upregulate HIF-2α, stabilize the eIF4E2 translation initiation complex, and activate PDGFRA and secrete VEGF in a non-mTOR-dependent manner.^544^ Abnormal angiogenesis is often accompanied by ECM remodeling, with both processes complementing each other. The KSHV genome encodes 25 mature miRNAs to varying degrees, which have been shown to increase the expression of MMP1, MMP13, VEGFA, and VEGFR2 transcripts.^541,545^ In summary, KSHV primarily promotes KS growth and metastasis by enhancing angiogenic factors and MMP-mediated ECM remodeling.

KSHV has a neurotropic tendency. It can infect neuronal cells and promote the proliferation of neuronal cells through the Notch and Hippo signaling pathways.^546,547^ These infected neurons can spontaneously switch between the latent and lytic phases of infection and play an important role in regulating host genes related to tumorigenesis.^547^ NGS and bioinformatics analysis also found that KSHV infection leads to the expression of multiple neuronal and neuroendocrine genes in epithelial cells, thus providing survival advantages and immune evasion pathways for host cells.^548^ However, the relationship between these abnormal neuronal cells and neuroendocrine factors in the development of KS still requires further investigation.

HIV infection can also facilitate the angiogenesis induced by KSHV. HIV Tat can directly degrade IκBα or inhibit IκBα activity by upregulating hsa-miR-891a-5p, thereby activating the NF-κB/VEGF pathway, which synergizes with KSHV K1-induced angiogenesis.^549^ Similarly, the HIV-Nef protein can directly downregulate PTEN or inhibit PTEN activity, thereby activating the PI3K/AKT/mTOR pathway, which synergizes with KSHV K1 protein to promote angiogenesis (Fig. 8g).^549^

##### Metabolic reprogramming

The metabolic reprogramming of host cells driven by KSHV has been demonstrated to play a role as a catalyst in the malignant transformation process.^550^ KSHV encodes vGPCR and KSHV miRNAs that prevents the degradation of HIF-1α and activates HIF-1α.^551^ Recent studies have shown that KSHV vCyclin binds and hijacks cyclin-dependent kinase CDK6, phosphorylating Ser-1900 on CAD, thereby activating pyrimidine synthesis and glycolytic reprogramming.^552^ KSHV infection also increases non-essential amino acid metabolites, with in vitro 3D culture further confirming that this change is driven by the activation of PYCR enzyme.^553^ This increase in amino acid catabolism is a crucial pathway for energy replenishment under glucose metabolism restriction. In addition to enhancing aerobic glycolysis and amino acid catabolism, KSHV also enhances lipogenesis and peroxisome-mediated lipid β-oxidation, though the specific mechanisms require further research.^554^ Studies have found that LPS of *Escherichiacoli* can promote KSHV-induced cell transformation and tumorigenesis in a KS-like mouse model.^546^ Oral involvement is one of the common clinical manifestations of this KS. A cross-sectional study found that compared with individuals without oral KS, the α-diversity and richness of the oral microbiota in HIV/KSHV co-infected individuals with oral KS were significantly reduced, specifically manifested as an increase in the *Firmicutes* and *Actinobacteria phyla* and a decrease in the *Proteobacteria phylum*.^555^ Among them, *Firmicutes* and its metabolites may promote KS tumorigenesis by inducing an inflammatory response and reactivating KSHV.^555^ Another study has shown that Staphylococcus aureus in the oral cavity can promote KSHV lytic reactivation by upregulating ROS and inhibiting the cyclinD1-Dicer-viral microRNA axis, leading to KS tumorigenesis.^556^ These studies explain why HIV/KSHV-infected individuals who suffer from opportunistic bacterial infections and metabolic complications are prone to developing KS (Fig. 8h).^557^

### Lymphoma

**Fig. 9:**
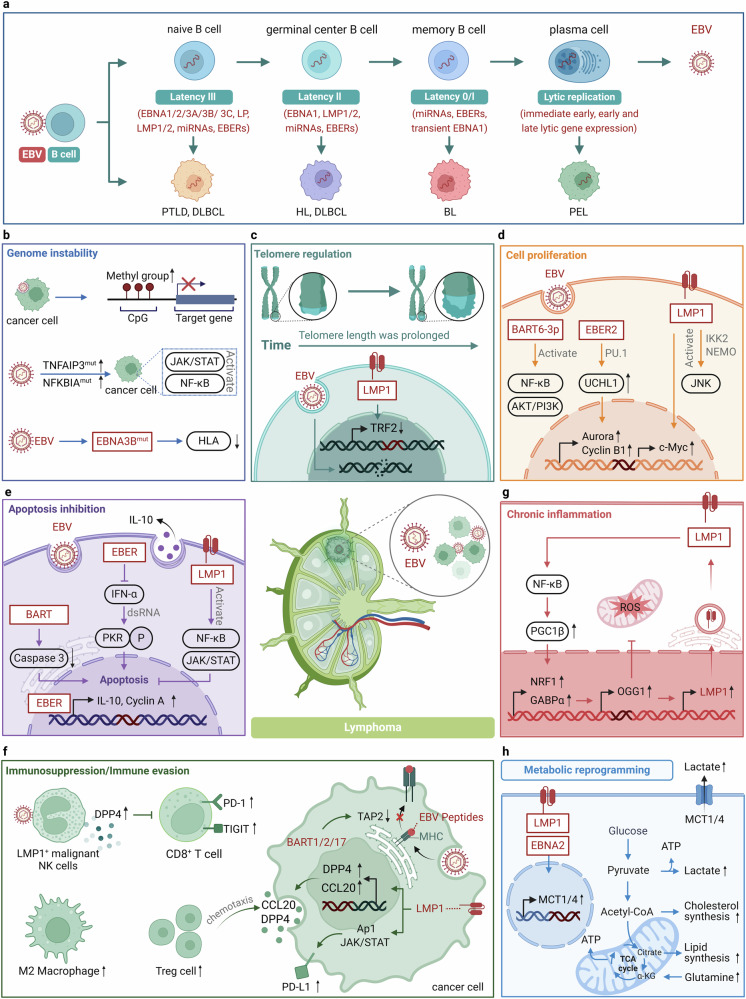
Oncogenic mechanisms of EBV in lymphoma. **a** Progression of lymphoma caused by EBV. **b** EBV infection induces high CpG island methylation, frequent gene mutations, and increased DNA damage. **c** LMP1 induces telomere instability through the downregulation of TRF2. **d** EBV activates NF-κB, AKT/PI3K, and JNK signaling pathways to enhance cell proliferation. **e** EBV inhibits cell apoptosis by increasing IL-10 secretion, activating NF-κB and JAK-STAT pathways, and suppressing IFN-related apoptotic signaling pathways. **f** EBV promotes immune evasion by reducing antigen presentation and upregulating PD-L1, contributing to an immunosuppressive microenvironment through Treg cell recruitment, M2 macrophage infiltration, and enhanced inhibitory receptor expression on CD8^+^ T cells, alongside increased oxidative stress. **g** LMP1 inhibits ROS-induced DNA damage by activating the PGC1β/HKDC1/OGG1 signaling pathway. **h** EBV induces metabolic reprogramming by upregulating intracellular MCT1/4 expression and promoting lipid synthesis. Red text: Components of EBV; Black text: Components and biological processes of the host cell; Direct oncogenesis: **b**–**e**; Indirect oncogenesis: **g**, **h**. This figure was created with BioRender.com

EBV is implicated in the pathogenesis of several lymphomas, including extranodal natural killer/T-cell lymphoma (NKTCL), Burkitt lymphoma (BL), and Hodgkin lymphoma (HL).^558^ (1) NKTCL frequently occurs in extranodal sites, particularly in the nasopharyngeal region, skin, and gastrointestinal tract.^559^ EBV can be detected in nearly all NKTCL cells, with most cases exhibiting Latency II, characterized by the expression of EBNA1, BART, LMP1, LMP2A, and LMP2B.^560,561^ (2) BL has three subtypes: endemic (eBL), sporadic (sBL), and immunodeficiency-related form (idBL).^562^ The association with EBV varies among these subtypes; almost all eBL tumors are EBV-positive, while about 15-30% of sBL and 25-40% of idBL cases harbor EBV.^32,563,564^ BL is associated with EBV Latency I and II.^41^ (3) HL is divided into classical HL (cHL) and nodular lymphocyte-predominant HL (NLPHL).^565^ In cHL, tumor cells are known as Hodgkin/Reed Sternberg (HRS) cells, whereas in NLPHL, they are referred to as lymphocyte-predominant (LP) cells.^566–568^ In Western countries, approximately 30% of cHL cases show latent EBV infection in HRS cells. The three EBV-encoded proteins expressed in infected HRS cells—EBNA1, LMP1, and LMP2A—as well as viral miRNAs, are implicated in the pathogenesis of cHL.^569,570^ Based on the viral gene expression patterns, EBV establishes one of the three latency patterns. In latency type I, EBNA-1 and two small non-coding EBERs are expressed, and it is generally considered to be associated with BL.^571^ In latency type II, the virus expresses EBNA-1, EBER, and latent membrane proteins (LMP), namely LMP-1, LMP-2A, and LMP-2B.^571^ This latency pattern is related to Hodgkin lymphoma. Finally, latency type III leads to the expression of the entire EBV repertoire, including EBNA, EBER, and LMP, and is associated with post-transplant lymphoproliferative disorders (PTLD) and other immunocompromised states.^571,572^ EBV-positive DLBCL is associated with latency type II and type III patterns.^573^ Lytic EBV replication occurs after plasma cells differentiate from this persistent pool and is associated with PEL.^574^ HBV and HCV infections can not only cause liver complications, such as cirrhosis and HCC, but also extrahepatic complications including B-NHL.^575^ And lymphoma is the most common malignant tumor in PLWH.^576,577^ The risk of developing NHL in PLWH is 23 times higher than in the general population, and approximately 7% of NHL cases can be attributed to HIV infection (Fig. 9a).^577^

#### Direct oncogenesis: leading to the emergence of virus-related tumors

##### Oncogene activation and tumor suppressor gene inhibition

EBV plays multiple roles in the development of B-cell lymphomas. EBNA2 promotes B-cell immortalization and acts as a transcriptional activator that directly upregulates the oncogene c-Myc.^578^ Almost all Burkitt lymphomas carry chromosomal translocations related to the c-Myc oncogene.^579^ Recent studies have found that latent EBV infection, in synergy with c-Myc overexpression, can induce BL-like human B-cell lymphoma in mouse models.^580^ Additionally, Ikeda et al. observed that BL expressing LMP1 exhibits a lower frequency of p53 mutations, suggesting that LMP1 may reduce the incidence of p53 mutations in human BL, possibly indicating a novel pathogenic role.^581^ Furthermore, EBV promotes B-cell lymphoma by downregulating a novel p53-responsive lncRNA, IGFBP7-AS1.^582^

##### Genome instability

The interaction between EBV and the host significantly influences the development of B-cell lymphomas, particularly through the role of epigenetic markers in gene regulation.^583,584^ In EBV-related BL, the host genome typically exhibits global hypomethylation, although specific sites, particularly within CpG islands, may be hypermethylated.^585^ LMP1 can induce the aberrant activation of multiple signaling pathways in HRS cells, such as NF-κB and JAK/STAT, which are also activated in specific cell mutations.^586,587^ Additionally, in EBV-related cHL, negative regulators of the NF-κB pathway, such as TNFAIP3 and NFKBIA, are frequently mutated.^588,589^ In NKTCL, EBNA3B exhibits a high density of nonsynonymous mutations, potentially affecting immune escape mechanisms related to HLA-restricted epitopes, thereby promoting NKTCL development and progression.^589,590^ LMP1 induces telomere instability by downregulating telomere repeat binding factor 2 (TRF2), promoting the formation of HRS cells (Fig. 9b, c).^586^

##### Interfering cell proliferation and cell cycle

EBV is critical in developing B-cell lymphomas through its oncoproteins and ncRNAs. The proliferative activity of LMP1, LMP2A, and EBNA2, along with inhibiting terminal differentiation by EBNA3A, drives the progression of EBV-mediated B-cell lymphomas.^591^ EBNA2 disrupts c-Myc expression, increasing cell proliferation.^580^ Cells co-expressing LMP1 and EBNA2 can develop lymphomas in NOG mice, demonstrating the importance of EBV oncoproteins in B-cell transformation.^592^ Additionally, LMP1 can activate the oncogenic JNK signaling pathway through IkB kinase 2 (IKK2) and its cofactor NEMO, with p62 playing a pivotal role in EBV LMP1-mediated signaling; p62 deficiency enhances apoptosis and inhibits the proliferation of lymphoblastoid cell lines (LCLs).^593^ LMP2A can synergize with c-Myc and mutant Cyclin D3 to promote B-cell proliferation and survival, suppressing c-Myc-induced apoptosis and advancing the cell cycle by inhibiting RB1 function, thus facilitating B-cell malignant transformation.^594^ EBNA1 is crucial for the persistence of the viral genome during latency, preventing the clearance of infected cells by the immune system, promoting cell proliferation and growth, and protecting infected cells from apoptosis.^595^ LMP1 can transform B cells in vitro, significantly enhancing malignant traits by activating the NF-κB, PI3K/AKT/mTOR, and RAS/MAPK signaling pathways.^470^ The small EBV-encoded RNA EBER is implicated in the malignant phenotype of BL and is associated with resistance to apoptosis in BL cells, as well as increased autocrine expression of the growth factor IL-10.^596^ EBV synergizes with c-Myc to induce BL-like lymphomas, where c-Myc promotes the development of BL by inhibiting the expression of LMP1.^580^ Additionally, EBV-encoded proteins can prevent the differentiation of activated B cells, contributing to mutations that drive tumor transformation. LMP1, for example, rescues germinal center B cells lacking B-cell receptors (BCR) from apoptosis and plays a crucial role in the formation of HRS cells, potentially inducing B-cell survival and proliferation signaling through NF-κB, JAK-STAT, c-Myc, and Cyclin A pathways.^597^

Furthermore, EBV ncRNAs also significantly impact the development of B-cell lymphomas. Several EBV miRNAs are highly expressed in BL, particularly EBV-miR-BART7, EBV-miR-BART10, EBV-miR-BART11-3p, EBV-miR-BART6-3p, and EBV-miR-BART17-5p.^598,599^ EBER2 promotes the expansion of latently infected B cells by inhibiting their terminal differentiation.^600^ Additionally, EBER2 accelerates B-cell proliferation by enhancing the expression of the deubiquitinase UCHL1 and increasing the levels of Aurora kinase and Cyclin B1, an effect also observed in BL.^601^ Various non-coding RNA transcripts in infected cells help activate proliferation-promoting signaling pathways (e.g., BPLF1), inhibit apoptosis (e.g., BART), and regulate mechanisms that shield infected cells from immune system attacks (e.g., BORF2, BNLF2A, BDLF3, vIL-10, BZLF2).^470,602^ In EBV-positive BL, the inhibition of apoptosis is at least partially mediated by viral EBV-BART miRNAs, which reduce the expression of apoptosis-inducing proteins like caspase 3 (Fig. 9d, e).^603,604^

#### Indirect oncogenesis: promoting the progression of virus-related tumors

##### Immunosuppression

In EBV-positive NHL, there is a higher infiltration of CD8^+^ T cells, along with increased expression of Tregs, M2 macrophages, and immunosuppressive molecules, creating a more tolerant immune environment compared to EBV-negative cases.^605^ In EBV-positive cHL, the TME is characterized by a high density of CD8^+^ T cells expressing inhibitory receptors like PD-1 and TIGIT, along with specific macrophage subsets and an in situ inflammatory molecular profile associated with TCR and BCR signaling pathways.^606^ Li et al. used single-cell analysis to demonstrate the significance of LMP1^+^ malignant NK cells in NKTCL, showing that these cells secrete dipeptidyl peptidase-4 (DPP4) and regulate chemokines, affecting immune cell chemotaxis and exhibiting immunosuppressive effects on T cells, thereby promoting tumor development and progression.^607^ EBV latent genes also contribute to the unique TME of cHL. LMP1 recruits TME cells by inducing the expression of various chemokines and cytokines in infected HRS cells.^608^ EBNA1 has been found to increase the expression of the T-cell chemokine CCL20, facilitating the recruitment of Treg cells.^609^ EBV-encoded BART miRNAs promote lymphoma formation by regulating viral and cellular gene expression and promote lymphoma formation by regulating viral and cellular gene expression and impact innate and adaptive immunity.^610^ Specifically, EBV-miR-BART1 and EBV-miR-BART2 reduce antigen presentation, while EBV-miR-BART17 targets Transporter associated with Antigen Processing 2 (TAP2), which is involved in antigen processing and presentation.^610–612^

EBV infection induces PD-L1 expression in cHL, a process mediated by LMP1 and primarily involving the JAK/STAT and AP-1 signaling pathways.^613,614^ Blocking the PD-1/PD-L1 interaction can successfully inhibit the growth of EBV-induced lymphomas in mouse models, suggesting a possible interaction between EBV and the PD-1/PD-L1 pathway in tumor immunology.^615^ Furthermore, EBV infection in NKTCL may induce immune tolerance by upregulating PD-L1 expression through LMP1 (Fig. 9f).^616,617^ Although HIV does not directly cause NHL, due to the impaired immune function of PLWH, they are more susceptible to coinfection with other oncogenic viruses, such as EBV.^618–620^ In addition, HIV can directly participate in viral activity through immune suppression, immune dysregulation, and chronic antigen stimulation, playing a role in carcinogenesis and lymphoma^621^ H. pylori, is also thought to trigger lymphoma, which continuously stimulates antigen presentation, thereby inducing the expansion of B cells. In humans, this phenomenon is manifested as the overexpression of specific V genes.^622^ Although lymphocytes and microorganisms are typically separated by the epithelial barrier, bacteria, antigens, or metabolites can traverse the mucosal barrier via dendritic cells or M cells, which constantly sample the lumen.^623^

##### Chronic inflammation stimulation and oxidative stress

LMP1 is a potential driver of EBV-mediated lymphoma, functioning through the activation of the PGC1β/HKDC1/OGG1 signaling pathway. Additionally, LMP1 activates the SREBP1-mediated hexokinase domain component 1 (HKDC1) signaling pathway, leading to mitochondrial dysfunction, and forming a positive feedback loop of ROS bursts. This mechanism inhibits EBV replication and suppresses the growth of NKTCL via the LMP1/PGC1β signaling pathway (Fig. 9g).^624^

##### Metabolic reprogramming

EBV plays a crucial role in regulating B-cell metabolism and cell cycle progression through the interaction between EBNA2 and EBF1 during EBV infection.^625^ LMP1, a direct target gene of EBNA2, acts as a significant metabolic regulator in many EBV-related tumor cells by enhancing aerobic glycolysis.^626^ Additionally, EBV-infected B cells induce the expression of monocarboxylate transporter 1 (MCT1) and monocarboxylate transporter 4 (MCT4) through EBNA2 and LMP1, respectively, which are crucial for B-cell proliferation. Dual inhibition of MCT1/4 results in growth arrest of LCL cells and lactate accumulation, impacting oxygen consumption and glutathione levels, further revealing the metabolic characteristics of EBV-infected cells.^627^ LMP1 also drives metabolic reprogramming of B cells by inducing fatty acid synthase (FASN) and lipid droplet formation. Inhibiting lipogenesis effectively kills LMP1^+^ B cells and prevents EBV-induced B-cell immortalization, suggesting that targeting LMP1-induced lipogenesis could be an effective strategy for treating EBV-related tumors (Fig. 9h).^628^

### Adult T-cell leukemia/lymphoma (ATLL)

**Fig. 10:**
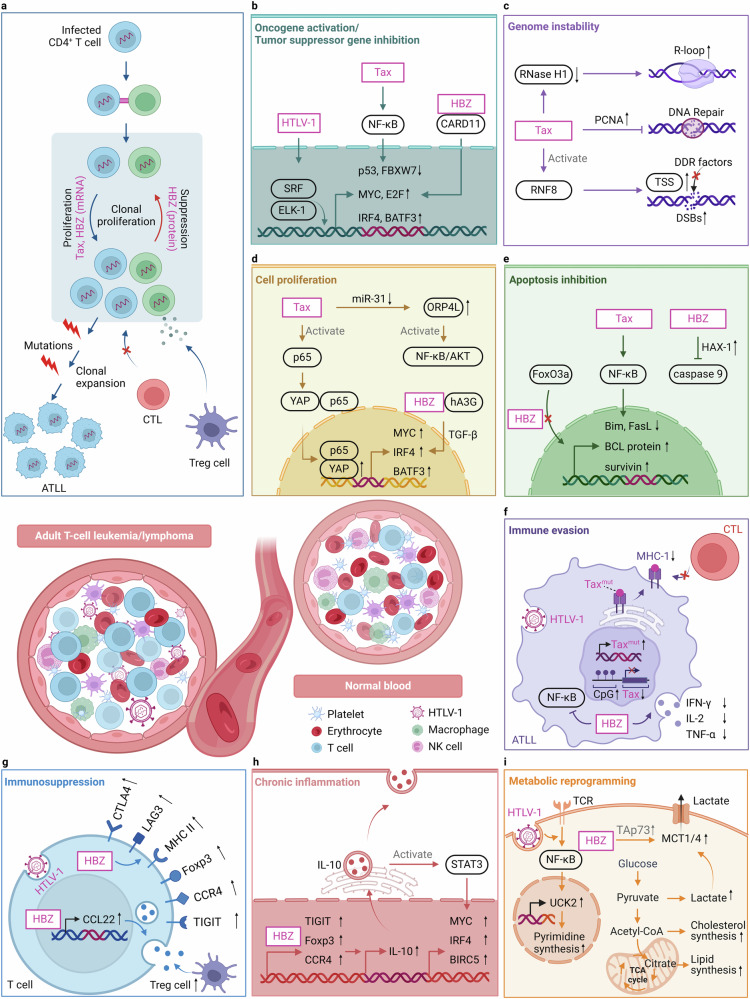
Oncogenic mechanisms of HTLV-1 in adult T-cell leukemia/lymphoma. **a** Progression of ATLL caused by HTLV-1. **b** HTLV-1 encoded proteins upregulate oncogenes and silence tumor suppressor genes via NF-κB-dependent and NF-κB-independent pathways, recruiting transcription factors to gene promoters. **c** The viral Tax protein increases genomic instability by promoting DNA damage and inhibiting DNA double-strand break repair. **d** HTLV-1 infection boosts cell proliferation through persistent activation of AKT/NF-κB and TGF-β/Smad pathways, and promotes YAP nuclear translocation. **e** HTLV-1 encoded proteins inhibit apoptosis by suppressing caspase signaling, activating the NF-κB pathway, and preventing FoxO3a nuclear translocation. **f** HTLV-1-infected host cells achieve immune escape by reducing Tax protein expression, generating Tax mutants, and suppressing Th1 cytokine production. **g** HTLV-1 mediates immune suppression by inducing inhibitory receptor expression on T cells and recruiting Treg cells. **h** HTLV-1 induces chronic inflammation by causing host cells to overproduce IL-10. **i** HTLV-1 infection enhances intracellular glycolysis, pyrimidine biosynthesis, and lipid synthesis. Fuchsia text: Components of HTLV-1; Black text: Components of host cell; Direct oncogenesis: **b**–**e**; Indirect oncogenesis: **f**–**i**. This figure was created with BioRender.com

HTLV-1 induces carcinogenesis through insertional mutagenesis and epigenetic modifications (Fig. 10).^629^ Therefore, high HTLV-1 proviral load (PVL) is a risk factor for HTLV-1-associated inflammation and ATLL.^630^ Besides, high PVL often closely relates to poor treatment efficacy and prognosis of ATLL.^631^

The oncogenic effects of HTLV-1 primarily depend on two viral proteins: Tax and HBZ. After HTLV-1 proviral integration, HBZ is continuously expressed at a low level,^632,633^ while Tax is expressed intermittently in a burst manner. In the early stage of infection, Tax, as a transcriptional activator, can promote viral replication and the proliferation of infected cells, which is conducive to viral reactivation and de novo infection.^634^ Due to the high immunogenicity of Tax, CTLs exert selective pressure on Tax-positive cells. Moreover, the continuously expressed HBZ will inhibit Tax expression to evade immune surveillance, and eventually prompt the virus to enter the latent period.^635,636^ The long latent period provides ample time for the virus to interact with host cells (Fig. 10a).

#### Direct oncogenesis: leading to the emergence of virus-related tumors

##### Oncogene activation and tumor suppressor gene inhibition

HTLV-1 provirus integrates near proto-oncogenes, influencing their expression through cis-perturbation.^637^ Mechanistically, HTLV-1 recruits host transcription factors SRF and ELK-1 to nucleosome-free regions within the provirus DNA, promoting the expression of oncogenes near HTLV-1 integration sites.^638^

HTLV-1 integration can lead to mutations or functional inactivation of tumor suppressor genes, such as p53 and FBXW7, and the silencing of these tumor suppressor genes is one of HTLV-1’s key carcinogenic mechanisms.^639,640^ Moreover, Tax protein activation of the NF-κB signaling pathway also inhibits the expression of p53 and FBXW7.^142,635,641,642^ HBZ protein cooperates with CARD11, a protein required for T and B cell activation,^643^ and upregulates oncogenes like IRF4, BATF3, MYC, and E2F via non-canonical NF-κB pathways, inducing ATLL.^644,645^ In summary, HTLV-1 disrupts the balance between oncogene activation and tumor suppressor gene inhibition, leading to uncontrolled cell growth (Fig. 10b).

##### Genome instability

HTLV-1 infection causes oncogenic mutations, chromosomal rearrangements, and DNA damage, compromising the genomic stability of host cells. Studies have shown that HTLV-1 infection increases cell mutation rates and copy number variations.^646^ Additionally, Tax protein also downregulates RNase H1, causing excessive R-loop accumulation and inducing DSBs.^647^ Simultaneously, Tax triggers the activation of RNF8, thereby giving rise to Tax-speckle structures (TSS). These TSSs sequester DDR factors, disrupting DSBs repair and exacerbating genomic instability in host cells.^648^ The latest research further reveals that Tax coordinates transcriptional and alternative splicing events during NF-κB activation, upregulates intra-Topologically associating domain inter-contact remodeling of 3D chromatin structure in host cells, which lays a poor genetic foundation for the occurrence and development of HTLV-1-related diseases (Fig. 10c).^649,650^

##### Interfering cell proliferation and apoptosis

Tax and HBZ proteins play crucial roles in the proliferation and survival of T cells. Tax upregulates p65, which stabilizes YAP and promotes its nuclear translocation, leading to the expression of proliferation-related genes.^634^ Recent studies have also found that Tax induced hsa-miR-31 loss,^651^ resulting in sustained activation of the AKT/NF-κB oncogenic signaling pathway.^652^ In contrast, the regulatory effect of HBZ protein on T-cell proliferation exhibits a dual nature. HBZ can inhibit Tax-activated NK-κB signaling and suppress T-cell proliferation in vitro.^653,654^ However, in vivo experiments suggest that HBZ promotes malignant T-cell proliferation. For instance, HBZ binds to human APOBEC3G, activates the TGF-β/Smad signaling pathway, upregulates BATF3/IRF4 and MYC transcription, and promotes T-cell proliferation (Fig. 10d).^655^

Tax protein activates the NF-κB pathway, enhancing the expression of anti-apoptotic proteins such as, survivin, and BCL family molecules.^656,657^ Similarly, HBZ protein inhibits apoptosis by reducing HAX-1 protein ubiquitination and inhibiting caspase-9 activation.^658^ Additionally, HBZ interferes with the localization and function of transcriptional activator FoxO3a, reducing the expression of apoptotic genes Bim and its ligand gene FasL, further inhibiting apoptosis (Fig. 10e).^659^

#### Indirect oncogenesis: promoting the progression of virus-related tumors

##### Immunosuppression

HTLV-1 employs multiple strategies to downregulate the immune surveillance mechanism, thereby evading the attacks of the host immune system. Tax, as a viral protein with strong immunogenicity, can activate the classical NF-κB signaling pathway in CTLs, and then mediate cellular immunity.^641^ However, Tax is usually expressed in the early stage of infection or intermittently expressed in the late stage of infection, and this expression pattern reduces the specific recognition of it by CTLs.^636^ In addition, studies have shown that after HTLV-1 infection, host cells are more prone to 5'-LTR deletion,^660^ hypermethylation of viral promoter DNA,^661^ and Tax mutants that are not easily recognized by CTLs will be generated.^655^ These mechanisms work together to promote the immune evasion of HTLV-1. Different from Tax, HBZ not only selectively inhibits the classical NF-κB signaling pathway to achieve immune evasion,^662^ but also reduce the expression of Th1 cytokines, such as IFN-γ, IL-2, and TNF-α, thereby mediating the establishment of an inhibitory immune microenvironment (Fig. 10f).^663^

The transformation of T cells into ATLL cells induced by HTLV-1 is accompanied by an increase in immunosuppressive characteristics, such as the upregulation of CTLA4, LAG3, and MHCII.^151^ The HBZ protein can directly induce the expression of immunosuppressive receptors, such as Foxp3, CCR4, and TIGIT.^664^ Additionally, the Tax protein upregulates the secretion of the chemokine CCL22, which attracts and maintains a high-frequency circulation of CD4^+^ Foxp3^+^ CCR4^+^ Treg cells (Fig. 10g).^665^

##### Chronic inflammation stimulation

IL-10 is a key factor in conferring malignant proliferative abilities to HTLV-1-infected T cells. Studies have shown that the HBZ protein upregulates IL-10 levels by inducing the overexpression of TIGIT, Foxp3, and CCR4.^666^ Additionally, the persistent activation of interferons due to chronic HTLV-1 infection is another cause of IL-10 over-secretion.^667,668^ High levels of IL-10 promote STAT3 phosphorylation, increasing the expression of BIRC5, MYC, and IRF4, which contributes to the spontaneous growth and survival of T cells.^669^ The immunosuppressive microenvironment of HTLV-1 is also influenced by IL-10. Cells infected with HTLV-1 secrete IL-10, which can suppress host immune responses through paracrine signaling while supporting the proliferation of ATLL cells through autocrine signaling, achieving a dual effect.^149,669^ These studies suggest that the excessive secretion of IL-10 helps HTLV-1 infected cells evade host immune surveillance, thereby exacerbating the progression of ATLL (Fig. 10h).

##### Metabolic reprogramming

HTLV-1 hijacks glucose, lipid, and pyrimidine nucleotide metabolism, leading to the malignant transformation of T cells. The HBZ protein reduces EZH2 occupancy at the TAp73 promoter by binding to EZH2, while HBZ RNA activates TAp73 transcription. As TAp73 is upregulated, the transcription of lactate transporters MCT1 and MCT4 increases, enhancing glycolytic flux.^150^ In terms of pyrimidine nucleotide metabolism, UCK2 is a key regulator closely associated with cell proliferative capacity.^670^ The activation of the TCR-NF-κB signaling pathway by HTLV-1 leads to the accumulation of UCK2, thereby increasing pyrimidine biosynthesis and supporting the proliferation of transformed T cells (Fig. 10i).^671^

## Treatment strategies in specific cancer types

Given that viral infection is a primary pathogenic factor in the development of virus-related malignancies, preventive measures such as vaccination are crucial. For example, the HBV vaccine can reduce the risk of HCC,^672^ while the HPV vaccine is effective in preventing CC.^673^ For individuals already infected with these viruses, rigorous antiviral therapy is crucial for reducing cancer risk and is associated with improved long-term survival in those with virus-related malignancies.^674^ Some anti-tumor agents may lead to viral reactivation, which can have severe repercussions, making vigilant monitoring for viral reactivation necessary.^675^ Despite the detrimental role of viruses in tumor development, they also present opportunities for targeting and destroying virus-infected cancer cells. Approaches such as therapeutic vaccines, T cell receptor-engineered T (TCR-T) cells, and chimeric antigen receptor T (CAR-T) cells, which target viral antigens, have shown promise in treating virus-related tumors.^676,677^ Additionally, intervening in virus-related cancer signaling pathways can effectively inhibit tumor growth and spread. Changes in tumor immunity due to viral infections offer various treatment strategies, including immune checkpoint inhibitors (ICIs), bifunctional fusion proteins, and the combination of ICIs and targeted therapy.^678^ For the treatment of virus-related malignancies, viral therapy is crucial. It is a double-edged sword, showing many advantages as well as facing numerous challenges. The main advantages are as follows: First, it has a preventive effect. Viral preventive vaccines can induce the production of neutralizing antibodies, reducing the risk of cancer occurrence from the source.^679^ Second, precise gene-targeted therapy holds the promise of precisely attacking the viral oncogenes in tumor cells, minimizing damage to normal cells.^680^ Third, some new immune therapy targets against viruses have been discovered, which can provide new directions for treatment.^362^ Fourth, combination therapy has obvious advantages. It can be combined with chemotherapy, targeted therapy, immunotherapy, etc. For example, when combined with CAR-T cell therapy, it can activate and enhance the killing activity of immune cells against tumors.^681^ However, the disadvantages cannot be ignored. There are differences in the immune responses of individuals to vaccines. Some people may not produce sufficient antibodies after receiving preventive vaccines, and therapeutic vaccines are still not mature.^682^ Gene-editing technology has potential risks. The combination therapy is complex, and the optimal dosage and sequence of drugs need to be further studied and determined.

### HCC

#### HBV-related HCC

Chronic HBV infection is considered the main risk factor for the occurrence and development of HCC, with the lifetime risk of HCC in HBV carriers ranging from 10% to 25%.^679,683^ The treatment of HCC is characterized by multidisciplinary participation and the coexistence of various treatment methods, including surgical resection, liver transplantation, local ablation, targeted therapy (tyrosine kinase receptor inhibitors, multi-kinase inhibitors, VEGF monoclonal antibodies), immunotherapy (PD-1, PD-L1, CTLA4 monoclonal antibodies), vascular interventional therapy, radiotherapy, systemic therapies, and traditional Chinese medicine treatment.^672,684^ Clinically, most HCC patients are diagnosed at an advanced stage, having missed the opportunity for curative surgical treatment.^685^

Given the causes of HCC, early vaccination against HBV is crucial for preventing the occurrence of HCC.^686^ For patients with viral hepatitis, appropriate antiviral therapy can effectively inhibit viral replication, enhance virus-specific T-cell function, improve liver inflammation levels, and thus reduce the risk of HCC.^687^ Studies have shown that continuous antiviral treatment can reduce the risk of HCC by more than 50% in patients with chronic hepatitis B (CHB).^688^ In the current treatment strategies for HBV-related HCC, aside from routine vaccination for primary prevention and the utilization of antiviral drugs to inhibit viral replication and optimize the liver microenvironment, a rational combination of emerging therapies, such as targeted therapy, immunotherapy, and cell therapy, can intervene in the processes of tumor initiation, development, and metastasis via multiple biological pathways. This significantly enhances the comprehensive treatment efficacy of HBV-related HCC.

##### Virus vaccines and antiviral treatment

HBV vaccines are mainly divided into preventive vaccines and therapeutic vaccines. Common HBV vaccines are preventive and only prevent HBV infection in individuals who have not yet been infected.^689^ Therefore, a novel type of vaccine, the therapeutic HBV vaccine, has shown unique application value for HBV-infected individuals.^690^ A multicenter phase 2 clinical study showed that the DNA vaccine GS-4774 could enhance the activity of HBV-specific CD8^+^ T cells but failed to significantly reduce HBsAg levels. This suggests that GS-4774 alone is limited in efficacy and might be more effective when combined with other antiviral drugs to enhance antiviral immune responses.^691^ A lipid nanoparticle lipopeptide vaccine, εPA-44, demonstrated good safety in phase 2 clinical trials and continuously reduced HBsAg levels.^692^ The phase 2 clinical study results of the NASVAC vaccine suggest that it can induce anti-HBs and reduce HBsAg, with some patients achieving functional cure.^693^ Additionally, a therapeutic vaccine based on HBx,^694^ a dual-targeted HBV preS1 nanoparticle vaccine based on ferritin nanoparticles,^695^ and an mRNA vaccine encapsulated in lipid nanoparticles (LNP) have shown lasting potential in clearing HBV in mice in preclinical studies.^696,697^

Currently, antiviral drugs used to treat CHB include pegylated interferon (PEG-IFN) and oral Nucleos(t)ide analogs (NAs).^698,699^ Third-generation NAs, such as entecavir and tenofovir-based therapies (e.g., tenofovir disoproxil fumarate and tenofovir alafenamide) are recommended as the main treatment for CHB due to their high efficacy and low resistance.^700^ Studies have shown that, whether used alone or in combination, entecavir improves OS and PFS in patients compared to lamivudine.^701,702^ There is debate regarding whether tenofovir reduces the risk of HCC development in a secondary prevention setting compared to entecavir. Some studies indicate that patients receiving tenofovir after liver resection have a lower risk of HCC recurrence compared to those treated with entecavir.^703^ Recently, Chung et al. revealed that patients with HBV-related HCC who were treated with tenofovir therapy experienced a more favorable prognosis compared to those who received entecavir treatment.^704^ Despite long-term NAs therapy for HBV, less than 5% of patients have HBsAg loss after 12 months of treatment, highlighting the need for therapies capable of achieving functional cure.^705^ It has been reported that PD-1/PD-L1 can be used not only for cancer treatment but also for treating CHB. In HCC patients receiving ICI treatment, functional cure of HBV was observed, with a higher cumulative incidence of HBsAg loss compared to the control group.^706^ Additionally, GSK836/GSK3228836, an investigational antisense oligonucleotide drug, showed in its phase IIb clinical trial (B-Clear) that HBsAg clearance and HBV DNA negativity could be achieved following treatment, marking a significant advancement in the management of CHB.^707^ Lowering HBsAg levels can improve OS rates and reduce recurrence rates in HCC patients.^708,709^ Therefore, during HCC treatment, it is recommended to regularly monitor HBsAg levels and HBV DNA and carry out antiviral prevention to prevent fatal complications related to viral reactivation and enhance antitumor effects. In addition, HBsAg-specific antibodies can suppress plasma HBsAg and viral DNA loads in patients, but they are cumbersome and expensive to produce, and the virus will rebound once antibody treatment is stopped. A single-chain variable fragment (scFv) with high affinity for the HBV envelope protein antigenic loop (AGL) can be endocytosed by hepatocytes to inhibit viral particle secretion.^710,711^ Chimeric antigen receptor (CAR) forms and their Fc fusion forms (G12-scFv-Fc, G12-CAR-Fc) developed on this basis can more strongly inhibit serum HBsAg levels in HBV mouse models.^712^ Therefore, Fc-linked G12-scFv and G12-CAR may become new methods for reducing circulating HBsAg levels.

##### Targeting tumor cell signaling

HBV-associated proteins induce various intracellular signaling changes in tumor cells, and multi-targeted combination therapy is an important direction for the treatment of viral HCC. Sorafenib, a multi-kinase inhibitor, was the first targeted drug approved for HCC. Two large, randomized, controlled, international multicenter phase III clinical trials have demonstrated that sorafenib can delay tumor progression and extend the survival of patients with advanced HCC.^713,714^ Compared to patients with HBV-related HCC, patients with HCV-related HCC benefit more from sorafenib treatment.^715^ Another multi-kinase inhibitor, regorafenib, may be more beneficial for HBV-related HCC patients.^716^ It has been confirmed that TGF-β variants in liver diseases are closely related to viral hepatitis and HCC. Galunisertib, a specific inhibitor of TGF-β, achieves its antitumor effects by specifically inhibiting HCC growth and migration.^717^ Some studies suggest that Galunisertib does not show synergistic effects when combined with ramucirumab, an anti-VEGF receptor 2 antibody, for treating HCC,^717^ but combining it with sorafenib shows prolonged OS.^718^ Additionally, GPC3 is an important diagnostic marker for HCC, and its expression level in virus-related HCC is higher than in non-viral HCC,^719^ indicating the potential application of GPC3-targeted therapy in virus-related HCC. A phase I clinical trial demonstrated the safety and immunogenicity of a DNA vaccine encoding GPC3 in HCC.^720^

##### Immunotherapy

ICIs play a crucial role in the treatment of HCC. Common ICIs include PD-1 monoclonal antibodies (Nivolumab, Pembrolizumab, Sintilimab, Camrelizumab, Tislelizumab, Toripalimab, Penpulimab); PD-L1 monoclonal antibodies (Tezolizumab, Durvalumab, Envafolimab); and CTLA-4 monoclonal antibodies (Ipilimumab, Tremelimumab). It is worth noting that the strategy of combining immunotherapy with HCC treatment may provide better survival benefits in the HBV-related population,^721,722^ possibly due to the dual antiviral and antitumor effects of ICIs.^723^ However, studies have reported that caution is needed regarding viral reactivation during chemotherapy and ICIs treatment of virus-related HCC. Viral reactivation may lead to liver function deterioration. In a study, HBV reactivation occurred in HBsAg-positive HCC patients receiving anti-PD-1 or anti-PD-L1 immunotherapy,^724^ while no HBV reactivation or HBV-related liver damage occurred in patients who continued antiviral treatment.^725^ Moreover, combining IFN-α with anti-PD-1 treatment can improve the response rate in patients with unresectable HCC,^726^ highlighting the importance of combining immunotherapy with antiviral treatment.

##### Cell therapy

HBV-specific CAR-T and TCR-T cells targeting the HBV surface protein are two promising therapeutic approaches for virus-related HCC. These approaches involve genetically engineered T cells expressing classical T cell receptors or chimeric antigen receptors that specifically target HBV antigen.^727^ Preclinical studies have shown that CAR-T cells targeting HBV surface proteins exhibit potential anti-HCC activity, particularly CD39^+^ HBV-CAR-T cells.^728^ However, there are two major challenges in the clinical application of HBV-CAR-T cells. First, the high concentration of HBsAg in the serum of HBV-positive patients can directly bind to HBV-CAR-T cells, interfering with their ability to target cells. Additionally, HBV-CAR-T cells cannot recognize HCC cells with integrated fragments of HBV DNA.^676^ Therefore, there is still a long way to go before virus-specific CAR-T therapy can be effectively used in HCC treatment.

Wang and colleagues conducted a phase I clinical trial on HBV-TCR-T therapy targeting HBsAg in patients with advanced HBV-related HCC who had relapsed or failed previous first-line systemic treatments. The results demonstrated that the adoptive transfer of HBV-TCR-T cells is generally safe and well-tolerated, with specific targeting effects.^729^ SCG101 is an autologous, HBV-specific T-cell product expressing a TCR that recognizes an envelope-derived peptide (S20-28) on HLA-A2. Recent studies have shown that SCG101 exhibits significant antiviral and antitumor dual activity in treating patients with advanced HBV-related HCC, significantly extending patient survival.^730^ Due to the limited effectiveness of TCR-T in solid tumors, it is essential to develop next-generation HBV-TCR-T products, such as TCR-T cells that express IL-21R to improve their activity and survival.^731^ Additionally, ongoing investigations are exploring the combination of HBV-TCR-T cells with kinase inhibitors and/or PD-1 inhibitors for treating patients with advanced HCC (Table 3). This could provide more effective treatment options for HCC patients. Continued research efforts aim to identify more effective immune targets and optimize TCR-T transfection methods. TCR-T therapy promises to be a key player in the treatment of cancer, infectious diseases, and autoimmune disorders.

**Table 3:** The completed and recruiting clinical trials of virus-related HCC

Conditions	Interventions	Phases	Patients (N)	NCT number	Status	Study period	URL
HBV^+^ redirected HCC	mRNA HBV/TCR T-cells	PHASE1	10	NCT04745403	RECRUITING	2022/5/20 −2028/7/1	https://clinicaltrials.gov/study/NCT04745403
HBV^+^ advanced HCC	Atezolizumab bevacizumab	NA	51	NCT04180072	ACTIVE_NOT_RECRUITING	2020/3/12 −2025/12/31	https://clinicaltrials.gov/study/NCT04180072
HBV^+^ advanced HCC	HBV-TCR T Cells (LioCyx-M)	PHASE1|PHASE2	55	NCT05195294	NOT_YET_RECRUITING	2022/6/1 2027/5/1	https://clinicaltrials.gov/study/NCT05195294
HBV^+^ advanced HCC	HBV mRNA vaccine	PHASE1	9	NCT05738447	RECRUITING	2023/2/15 −2025/1/1	https://clinicaltrials.gov/study/NCT05738447
HBV^+^ advanced HCC	Durvalumab	PHASE2	43	NCT04294498	RECRUITING	2020/11/2 −2025/12/31	https://clinicaltrials.gov/study/NCT04294498
HBV^+^ HCC	Hepatect CP 50 I.E./ml infusion solution	NA	5	NCT05293158	NOT_YET_RECRUITING	2024/11/1 −2026/6/1	https://clinicaltrials.gov/study/NCT05293158
HBV^+^ HCC	pembrolizumab	PHASE2	30	NCT03419481	ACTIVE_NOT_RECRUITING	2018/4/30 −2025/12/30	https://clinicaltrials.gov/study/NCT03419481
HBV^+^ Advanced or Metastatic HCC	Lenvatinib tislelizumab	PHASE2	30	NCT05897138	NOT_YET_RECRUITING	2023/8/1 −2027/6/1	https://clinicaltrials.gov/study/NCT05897138
HBV^+^ HCC	Biological: SCG101	PHASE1|PHASE2	46	NCT05417932	RECRUITING	2022/10/26 −2025/10/1	https://clinicaltrials.gov/study/NCT05417932
HBV^+^ High Risk of HCC	Peginterferon alfa-2b Injection, Nucleos (t) ide Analog	NA	267	NCT05671315	RECRUITING	2019/7/3 −2026/4/1	https://clinicaltrials.gov/study/NCT05671315
HBV^+^ Advanced HCC	MTL-CEBPA Sorafenib	PHASE2	150	NCT04710641	ACTIVE_NOT_RECRUITING	2022/1/1 −2025/5/1	https://clinicaltrials.gov/study/NCT04710641

#### HCV-related HCC

Chronic HCV infection is a well-established risk factor for HCC, increasing the risk by 10–20 times.^679^ Unlike HBV infection, HCV can be cured.^732^ Following HCV clearance, overall mortality and the risk of developing HCC decrease significantly.^733^ Currently, no effective HCV vaccine is available.^734^ Besides vaccination and antiviral therapy, targeted therapy, immunotherapy, and CAR-T cell therapy are actively being explored for HCV-related HCC.

##### Virus vaccines and antiviral treatment

Currently, HCV vaccines are still under clinical investigation, and vector vaccines targeting T cells and heterodimeric E1-E2 vaccines targeting B cells have not shown promising results.^735–738^ Han et al. developed a DNA vaccine, GL-6150, and preliminary results showed that GRS-6150 could reduce the frequency of Treg cells and enhance HCV-specific T cell responses,^124^ potentially preventing reinfection in patients cured by DAAs.

The main strategy for reducing HCC incidence is achieving SVR (sustained virologic response) through antiviral therapy.^739^ In recent years, HCV treatment has entered the DAAs era, with interferon-free regimens being the preferred choice for HCV antiviral therapy. A systematic review shows that DAA treatment significantly reduces the risk of developing HCC compared to untreated individuals.^740^ Among chronic HCV patients who have never experienced HCC, the incidence of HCC after achieving SVR through treatment is 1.3%. However, among patients with a history of HCC, the recurrence rate remains as high as 29.6% after achieving SVR with DAAs.^741^ Recently, a 15-year follow-up study of Korean HCV patients further demonstrated that the use of DAAs is significantly associated with a reduced risk of liver fibrosis and HCC.^742^ Therefore, HCV-infected patients, regardless of their history of HCC, should receive DAA treatment early on to achieve SVR as soon as possible. Bourlière et al. reported in an RCT that sofosbuvir/ velpatasvir/voxilaprevir (SOF/VEL/VOX) achieved an SVR rate of 98% in patients who previously failed DAAs therapy.^743^ Thus, SOF/VEL/VOX monotherapy is the preferred retreatment strategy for those who experienced a previous DAA treatment failure.^16^

##### Targeting tumor cell signaling

For HCV-related HCC patients, sorafenib treatment slows tumor growth, alleviates liver function deterioration, and thus extends survival.^744^ An exploratory subgroup analysis of the SHARP trial indicated that HBV-negative and HCV-positive patients had increased OS with sorafenib treatment compared to placebo.^745^ This was confirmed by a pooled analysis of two randomized trials, suggesting that sorafenib may be more effective in HCV-related HCC patients.^746^ Additionally, an unsupervised transcriptome analysis conducted by Boyault et al. identified HCC subgroups with distinct genetic and molecular characteristics.^747^ Specifically, RAF-1,8 upregulation induced by HCV can be inhibited by sorafenib, which may contribute to the delay in HCV-related HCC progression mediated by sorafenib. Moreover, preclinical data indicate that sorafenib directly inhibits HCV viral replication,^748^ further supporting its efficacy in HCV-related HCC.

##### Immunotherapy

Research has shown that persistent HCV viremia leads to sustained upregulation of PD-1 and CTLA-4.^749^ To our knowledge, many previous studies on PD-1 inhibitors excluded patients with HCV infection. In 2013, a phase I clinical trial evaluated the efficacy of the CTLA-4 checkpoint inhibitor tremelimumab in a small cohort of advanced HCC patients with HCV infection. The trial reported manageable safety and preliminary evidence of antitumor and antiviral activity.^750^ However, evidence supporting the use of ICIs in treating advanced HCC patients with concurrent viral infections is still limited. El-Khoueiry et al. evaluated the safety and clinical benefits of nivolumab in the CheckMate 040 trial across multiple HCC etiologies, including HCV-infected patients. The results showed that disease control was achieved in 66% of HCV-related HCC patients, with a 6-month OS of 85%. However, nivolumab’s antiviral activity was limited, with some HCV-infected patients showing temporary reductions in HCV RNA,^751^ indicating that antiviral treatment might need to be combined. The controlled safety of nivolumab in this study lays the foundation for further research into PD-1 monoclonal antibodies as a treatment option for advanced HCV-related HCC patients.

##### Cell therapy

The first two CARs targeting HCV were designed based on broadly cross-reactive and cross-neutralizing human monoclonal antibodies targeting conserved epitopes on the HCV E2 glycoprotein (HCV/E2). Anti-HCV CAR-T cells have demonstrated good antiviral activity and the ability to lyse HCV/E2-transfected and HCV-infected target cells.^752^ This study suggests that the CAR-T cell may also apply to treating HCV. Additionally, HCV/E2 is the primary target of the host immune response and is prone to mutations.^753^ Therefore, identifying other conserved and essential antigens is crucial.

Given that viral infection is a primary pathogenic factor in the development of virus-related malignancies, preventive measures such as vaccination are crucial. For example, the HBV vaccine can reduce the risk of HCC,^672^ while the HPV vaccine is effective in preventing CC.^673^ For individuals already infected with these viruses, rigorous antiviral therapy is crucial for reducing cancer risk and is associated with improved long-term survival in those with virus-related malignancies.^674^ Some anti-tumor agents may lead to viral reactivation, which can have severe repercussions, making vigilant monitoring for viral reactivation necessary.^675^ Despite the detrimental role of viruses in tumor development, they also present opportunities for targeting and destroying virus-infected cancer cells. Approaches like therapeutic vaccines, T cell receptor-engineered T (TCR-T) cells, and chimeric antigen receptor T (CAR-T) cells, which target viral antigens, have shown promise in treating virus-related tumors.^676,677^

### Gastric cancer

EBVaGC is a specific subtype of gastric cancer, making up about 10% of all cases. Current gastric cancer treatment guidelines do not distinguish between EBV-positive and EBV-negative cases.^754^ There is no specific treatment for EBVaGC. Most patients undergo surgical resection, similar to other gastric cancers, but postoperative chemotherapy lacks targeted drugs.^755^ Early-stage EBVaGC typically shows a lower rate of lymph node metastasis and forms well-defined nodular lesions in the submucosa. It has less fibrosis than non-EBVaGC, which may allow for smaller surgical margins and the possibility of endoscopic electrocoagulation or dissection.^756^ For advanced EBVaGC, new treatment options have emerged alongside traditional surgical methods, including vaccines, targeted therapies, immunotherapies, and cellular therapies.^757^

Since EBVaGC is primarily caused by EBV infection, preventing and treating EBV is essential. One of the most promising approaches is developing an EBV vaccine.^758^ Research on EBV vaccines focuses on both prophylactic and therapeutic aspects, and developing an effective EBV vaccine is crucial for preventing and treating EBVaGC. Clinically, EBV infections are typically treated with supportive care and specific disease treatments. Routine antiviral therapy is not recommended.^759^ EBVaGC is characterized by significant lymphocytic infiltration and the presence of key immune molecules and potential therapeutic targets in the TME. Clinically available PD-1/PD-L1 ICIs and immunotherapies are expected to be applied in EBVaGC.^760,761^ Enhancing the anti-tumor capability of immune cells by targeting tumor cell signaling to improve therapeutic efficacy has been a research focus in recent years.

#### Virus vaccines

To date, no prophylactic vaccine for EBV has been marketed worldwide. EBV needs several membrane proteins to enter the cytoplasm of epithelial cells, B cells, and NK/T cells. EBV infection of B cells requires five membrane proteins (gp350, gH, gL, gB, and gp42), while EBV infection of epithelial cells requires four membrane proteins (BMFR2, gH, gL, and gB). These proteins on EBV could be effective targets for preventive vaccines against EBV.^762,763^ Two clinical trials with recombinant gp350 vaccines have shown good efficacy in preventing infectious mononucleosis caused by EBV infection, with an average efficacy of 78.0%. However, they did not affect asymptomatic EBV infections.^764,765^ This may be because antibodies from gp350 do not protect epithelial cells from EBV infection. Further research has found that EBV glycoproteins gH/gL and gB together mediate EBV fusion to B cells or epithelial cells. Vaccination with these proteins can induce antibodies that broadly prevent EBV infection.^766^ Two reports indicate that serum-neutralizing antibody levels from the gp350 monomer are lower than those from three dimers and trimers of gH/gL, trimeric gB, or four gp350 polymers.^767,768^ This may be due to the critical role of gH/gL and gB in EBV fusion and entry into B cells or epithelial cells. Thus, EBV gH/gL and gB may be more effective targets for a preventive EBV vaccine than gp350 alone. Additionally, combining gp350 with gH/gL and gB could create a next-generation candidate vaccine with clinical potential.^758^

Tumor treatment vaccines represent a promising new approach to cancer therapy. These vaccines improve the presentation of tumor antigens and stimulate the body’s immune surveillance by supplying external tumor antigens.^769^ An ideal tumor vaccine should deliver high-quality specific tumor antigens to dendritic cells (DCs), which then activate cytotoxic T lymphocytes (CTLs) to exert anti-tumor effects. Currently, EBVaGC-related vaccines are still under research, with no commercial vaccines available for EBV. The first approved EBV-related therapeutic mRNA vaccine, “WGc-043 Injection,” developed by WisTGen Biopharma, has recently received dual approvals in China and the United States. It is also the first therapeutic mRNA vaccine approved for clinical trials worldwide. The EBV mRNA vaccine shows significant advantages in terms of immunogenicity, safety, and efficacy, bringing new hope and options for the treatment of EBV-related tumors. Additionally, related clinical trials are underway to investigate the efficacy of the mRNA vaccine in GC (NCT05714748) (Table 4), and the results are eagerly anticipated.

**Table 4:** The completed and recruiting clinical trials of EBV-associated GC

Conditions	Interventions	Phases	Patients (N)	NCT number	Status	Study period	URL
EBV^+^ GC/ENKTCL	EBViNT Cell	Phase 1 Phase 2	72	NCT03789617	RECRUITING	2018/12/14 −2024/12/01	https://clinicaltrials.gov/study/NCT03789617
EBV^+^ Advanced GC	Poripalimab^+^Oxaliplatin	Phase 2	30	NCT05970627	NOT YRT RECRUITING	2023/7/28 −2029/07/28	https://clinicaltrials.gov/study/NCT05970627
EBV^+^ GC	Pembrolizumab, Capecitabine, and Radiation Therapy	Phase 2	40	NCT03257163	RECRUITING	2017/9/29 −2025/12/31	https://clinicaltrials.gov/study/NCT03257163
EBV^+^ Refractory Malignant Tumors	mRNA vaccine	Phase 1	9	NCT05714748	RECRUITING	2022/11/18 −2025/01/01	https://clinicaltrials.gov/search?cond=NCT05714748
Advanced EBV^+^ Solid Tumors	Nanatinostat Plus Valganciclovir	Phase 1 Phase 2	130	NCT05166577	RECRUITING	2021/10/08 −2025/10/01	https://clinicaltrials.gov/search?cond=NCT05166577

#### Targeting tumor cell signaling

Key targets for gastric cancer treatment include human epidermal growth factor receptor-2 (HER2), vascular endothelial growth factor receptor (VEGFR), Claudin 18.2, and microsatellite instability or mismatch repair deficiency (MSI/dMMR).^770^ For HER2-positive gastric cancer patients, adding trastuzumab to first-line or second-line chemotherapy improves survival rates, regardless of EBV infection status. Additionally, two novel antibody-drug conjugates (ADCs) targeting HER2, trastuzumab Deruxtecan and trastuzumab emtansine, are available for third-line and subsequent treatments of HER2-positive advanced gastric cancer. Furthermore, VEGF-targeting drugs like ramucirumab and apatinib are recommended for the second-line and third-line treatment of advanced or metastatic gastric cancer. Other targeted drugs for gastric cancer include the Claudin 18.2-targeting antibody Zolbetuximab, which has shown positive results in the GLOW and SPOTLIGHT studies, significantly prolonging PFS and OS. Currently, ongoing studies on EBV-related targets are providing new avenues for gastric cancer treatment.

#### Immunotherapy

Patients with HER-2-positive gastric cancer respond better to HER-2 targeted therapy and have more favorable prognoses than those with HER-2-negative gastric cancer.^771^ However, the majority of gastric cancers are HER-2 negative. Therefore, combining immunotherapy approaches is essential for improving outcomes in patients with advanced gastric cancer. The primary types of immunotherapy currently available are ICIs and cellular immunotherapy. Among these therapies, ICIs are the most widely used, particularly PD-1/PD-L1 monoclonal antibodies, which have garnered significant attention.^772,773^ Cellular immunotherapy is an emerging approach, including CAR-T therapy and NK cell therapy.

Around 80% of patients have PD-L1 expression in their tumor cells, while only 1.3% and 11.4% express PD-1 in tumor cells and infiltrating immune cells, respectively.^774^ These markers serve as significant therapeutic targets in cancer immunotherapy. EBVaGC exhibits substantial lymphocytic infiltration in the tumor immune microenvironment and responds well to immunotherapy,^331^ with high expression of PD-L1 and Ki67.^775^ In HER2-positive EBVaGC patients who are resistant to trastuzumab, clinical benefits can be achieved with PD-1 inhibitors used as monotherapy.^776,777^ Another study reported a 100% overall response rate (ORR) in EBVaGC patients treated with PD-1 inhibitors.^778^ Emerging data indicates that the ORR to PD-1 inhibitors in EBVaGC is approximately 25% higher compared to EBV-negative gastric cancer.^779–781^ Presently, PD-1 antibodies are widely used in the clinical treatment of gastric cancer. For first-line treatment of HER2-negative gastric cancer, pembrolizumab and nivolumab have shown survival benefits in patients with PD-L1 CPS ≥ 1.^772,773,782,783^ For HER2-positive gastric cancer, pembrolizumab has become the first anti-PD-1 drug approved for use in combination with trastuzumab and chemotherapy for first-line treatment of advanced patients.^784–787^ In 2020, Kim et al. validated the efficacy of immunotherapy in EBV-positive gastric cancer in a cohort of 300 gastric cancer patients, where 59.3% of the gastric cancer patients expressed PD-L1, associated with MSI and EBV-positive status.^788^ Moreover, pembrolizumab and nivolumab are recommended for second-line and beyond treatment in gastric cancer patients.^785,789–791^ While PD-1/PD-L1 antibodies have made remarkable advancements in gastric cancer treatment, their clinical efficacy as monotherapy is only observed in approximately 10% of patients. Thus, there is an urgent need for biomarkers that can predict clinical responses. Recently, Qiu et al. discovered high expression of the inhibitory immune checkpoint LAG3 in infiltrating CD8^+^ T cells in immune-resistant tumors. Subsequently, two patients with refractory EBV-positive gastric cancer participated in a clinical trial of LAG3 inhibitors and found that the LAG3 antibody effectively inhibited tumor growth and reduced peripheral blood EBV-DNA copy numbers. Targeting LAG3 could be a new therapeutic direction for EBVaGC patients.^362^

#### Cell therapy

CAR-T cell therapy is also being considered for the treatment of EBVaGC, with a variety of antigens including HER2, CEA, EpCAM, Claudin 18.2, and NKG2D potentially serving as targets for CAR T cell therapy.^310,792^ It has been reported that CAR T cell therapy can provide an additional 5 quality-adjusted life years, compared to non-drug therapies (surgery, radiotherapy, stem cell transplantation) which can provide 4.6 years, with similar levels of cost-effectiveness.^793^ Approximately 48% of gastric cancer patients have CLDN18.2 in their tumor tissues, which is linked to a poorer prognosis.^794^ In response to this, the only FDA-approved CAR-T cell therapy targeting CLDN18.2 is CT041. Recent results from a phase I trial revealed an ORR of 38.8% and a disease control rate (DCR) of 91.8%. The median PFS was 4.4 months, while the median OS was 8.8 months.^727,795^

As an alternative to CAR-T cell therapy, CAR-NK cell therapy has shown promising results against human GC cell lines in mouse models.^681^ Small phase I clinical trials indicate that NK cell therapy is safe and may have antitumor effects for advanced gastric cancer,^796,797^ which led to the FDA granting orphan drug designation to the NK cell therapy DF1001 in 2021.

### Cervical cancer

The development of CC is closely associated with HPV infection.^798,799^ In clinical practice, early-stage CC patients often undergo surgery or radiation therapy, with a 5-year OS rate of 80%–90%. Late-stage CC cases are primarily managed with systemic chemotherapy, but the survival outcomes remain poor.^800,801^ Most CC diagnostic and prognostic biomarkers are related to the molecular mechanisms of the HPV virus. Common ones include HPV DNA, E6/E7 mRNA, p53, Rb, and p16INK4a.^802^ HPV DNA in cervical epithelial cells is the most widely and deeply applied biomarker.^803^ With the development of liquid biopsy, circulating cell-free HPV DNA (cf HPV DNA) detection has emerged as a highly promising non-invasive method. It has a reported positive predictive value of 87.5% and a negative predictive value of 89.3% for disease progression.^804^ CircRNAs are also significant molecular markers for HPV-related cancers, useful for early diagnosis and prognosis prediction.^805^ Moreover, HPV E6/E7 mRNA can detect HPV infection and reflect the E6/E7 gene’s expression and activity, which helps predict the progression of cervical lesions and the clinical outcome of CIN2 p16-positive patients. Similar to HBV,^806^ vaccination is highly effective in preventing HPV infections.^807^ Beyond vaccination, emerging strategies in CC treatment include targeting tumor cell signaling pathways, immune checkpoint blockade, and boosting the cytotoxic T-cell response against HPV oncogenes.^808^ PLWH have a higher risk of HPV infection compared to HIV-negative individuals.^809^ Consequently, HPV-related CC is more prevalent among PLWH, and it not only progresses more rapidly but also results in a poorer prognosis.^810,811^ Currently, the treatment of HIV^+^ HPV^-^related CC mainly involves combining ART with antitumor therapy, with a focus on restoring immune function as part of individualized treatment.^812^

#### Virus vaccines

The oncogenic potential of HPV primarily depends on the continuous expression of viral proteins E6 and E7. Direct inhibition of E6 and E7 expression or their inactivation can help prevent HPV-related CC progression.^84^ Although no therapeutic vaccine is currently available for CC, research is advancing. The HPV nanoparticle vaccine cPANHPVAX, based on the E7 oncogene, shows promise in preclinical studies and is moving toward clinical trials.^813^ Phase I/IIa clinical trial confirmed the safety and efficacy of the therapeutic DNA vaccines VB10.16 and MEDI0457 (INO-3112) in HPV-related CC.^682,814^ Other vaccines, like Vvax001 and GX-188E, have demonstrated effectiveness in early clinical trials.^815,816^ Additionally, the HPV 16/18/31/33/45/52/58 vaccine has shown great efficacy, providing long-lasting protection.^817^ Moreover, combining vaccines with immunotherapy has shown potential by increasing CD4/CD8 T cell infiltration and preliminary antitumor activity.^818,819^

#### Targeting tumor cell signaling

Currently, the CRISPR/Cas9 system offers a targeted approach for treating HPV-related CC by specifically cutting the HPV E7 oncogene, thereby reducing E7 expression and inhibiting CC cell proliferation.^680^ Other CRISPR/Cas9 systems targeting E6 and E7 have also been proven to inhibit HPV-related CC tumor growth.^820–822^ Clinical trials exploring these CRISPR-based approaches are currently underway, potentially paving the way for novel therapeutic strategies. In addition, cervical cancer cells often upregulate PARP-1 and PARP-2, which participate in double-strand DNA break repair and promote tumor cell growth.^823–825^ The genomic instability of cells caused by HPV infection makes cancer cells more reliant on the DNA repair mechanism mediated by PARP. And PARP-1 has been found to be associated with HPV infection and the development of CC, especially significantly correlated with HPV-positive status in high-grade squamous intraepithelial lesions.^825^ Clinical studies have shown that the PARP inhibitor veliparib, when used in combination with paclitaxel and cisplatin, is effective in managing R/R CC.^826^

HIV promotes angiogenesis and ECM remodeling through various pathways, which contributes to the progression of CIN to cervical cancer. HIV protease inhibitors (HIV-PI) inhibit the MMP-9/VEGF angiogenesis axis by upregulating TIMP-3, making them potential drugs for preventing the progression and metastasis of CIN and cervical cancer.^827^ HIV protease inhibitors, such as nelfinavir, saquinavir, and lopinavir, have also been shown to reduce HPV16 E6 and E7 protein levels, restore wild-type p53 expression, and effectively induce apoptosis in HPV-CC.^828^ Tat, a key molecule in HIV’s acceleration of HPV-related CC, is a promising target for controlling the progression of HPV-related CC.^829^ Various approaches targeting Tat, such as Tat-conjugated H3 peptides,^830^ HIV TAT peptide-coated gold nanoparticles (GNPs)-ROR1 siRNA,^831^ TAT-chitosan-SPION nanoparticles,^831^ and anticancer agent P-bi-TAT,^832^ have demonstrated strong anticancer activity in vitro and in vivo, suggesting their potential as therapeutic candidates for HIV-related CC.

#### Immunotherapy

Immunostimulants and ICIs have shown potential in enhancing immune cell efficacy and improving treatment outcomes of HPV-related CC.^833^ Currently, the main therapeutic agents include PD-1 antibodies, PD-L1 antibodies, CTLA-4 antibodies, bispecific antibodies, and 4-1BB agonists.

HIV-related HPV^+^ CC shows increased PD-L1 expression.^834^ PD-1/PD-L1 inhibitors like pembrolizumab not only show therapeutic effects in HPV-related CC but also directly inhibit the positive transcription elongation factor b (P-TEFb) signaling pathway, activating HIV transcription.^835^ Reversing HIV latency could help clear HIV reservoirs.^836,837^ Similarly, a CTLA-4 inhibitor targets HIV-related cancers and reverses HIV latency, which helps eliminate cells with replication-competent HIV.^838^ Additionally, a Phase I clinical trial (GOG-9929) found that the CTLA-4 inhibitor ipilimumab exhibited immunomodulatory activity in patients with locally advanced CC, suggesting its potential as a promising treatment option.^839^ Research into dual-target immunotherapy is also advancing. Cadonilimab, a bispecific antibody targeting PD-1 and CTLA-4, showed an ORR of 66.7% in R/R CC patients when combined with chemotherapy in a Phase II clinical study.^840^

Preclinical investigations have revealed that immunostimulants can inhibit the proliferation of CC cells. Specifically, the immunostimulant imiquimod enhances local antiviral and antitumor immune responses by stimulating Toll-like receptor 7 (TLR7).^841^ A Phase I/II clinical study demonstrated that PF-04518600 (OX40 agonist) had an ORR of 11% in R/R CC patients, with no dose-limiting toxicity or grade 3-5 immune-related adverse events, suggesting it is safe and effective.^842^ In another Phase II clinical study, imiquimod was found to significantly increase the population of CD4^+^/CD8^+^ T cells in cases of high-grade CIN, leading to regression to grade 1 or lower CIN.^819^ Additionally, the combination of ipilimumab with chemotherapy displayed the capacity to elevate E6/E7-specific CTL responses, thus enhancing the treatment outcomes for CC patients.^839^

#### Cell therapy

Engineered TCR-T cells are currently being developed as a therapeutic approach for CC.^843–845^ Preclinical studies have shown that E7 TCR-T cells can specifically recognize and destroy HPV-positive tumor cell lines by targeting the E7 11-19 epitope complexed with HLA-A*02:01. In a Phase I clinical trial, E7 TCR-T cells demonstrated excellent safety and efficacy, achieving PR in 50% of patients.^846^ Building on these findings, Mansour Poorebrahim et al. combined E7-TCR with a CAR targeting TROP2 to develop E7-TCR/TROP2-CAR NK-92 cells, which demonstrated enhanced cytotoxic activity against HPV-positive CC cells.^847^ Similarly, Isaac Quiros-Fernandez et al. developed E7-TCR/L1CAM-CAR NK 92 cells with enhanced the cytotoxic capability.^848^ Numerous other HPV-specific engineered TCR-T cells are also undergoing clinical trials (Table 5).

**Table 5:** The completed and recruiting clinical trials of virus-related CC

Conditions	Interventions	Phases	Patients (N)	NCT number	Status	Study period	URL
HPV^+^ Cervical or Oropharyngeal Cancer	Lenti-HPV-07	PHASE 1 PHASE 2	72	NCT06319963	NOT_YET_RECRUITING	2024/03/01 −2026/12/01	https://clinicaltrials.gov/study/NCT06319963
cfHPV-DNA plasma positive patients	Cisplatin gemcitabine	PHASE 3	365	NCT05764044	RECRUITING	2024/03/27 −2026/12/31	https://clinicaltrials.gov/study/NCT05764044
HPV16^+^/HPV18^+^ CC failure to first-line platinum-based chemotherapy	BAVC-C^+^ Durvalumab	PHASE 2	37	NCT04800978	NOT_YET_RECRUITING	2021/06/14 −2024/08/31	https://clinicaltrials.gov/study/NCT04800978
R/M HPV16^+^ CC	Cemiplimab ISA101b Vaccine	PHASE 2	113	NCT04646005	ACTIVE_NOT_RECRUITING	2021/06/28 −2024/10/22	https://clinicaltrials.gov/study/NCT04646005
HIV ^+^ CIN	Fluorouracil cream Placebo	NA	180	NCT05413811	RECRUITING	2023/03/22 −2025/12/31	https://clinicaltrials.gov/study/NCT05413811
HIV ^+^ CIN	Intravaginal 5-Fluorouracil	PHASE 1	12	NCT05362955	ACTIVE_NOT_RECRUITING	2023/04/26 −2024/08/01	https://clinicaltrials.gov/study/NCT05362955
Advanced solid tumors in people living with HIV	Cabozantinib Nivolumab	PHASE 1	18	NCT04514484	ACTIVE_NOT_RECRUITING	2021/11/22 −2025/11/02	https://clinicaltrials.gov/study/NCT04514484
HPV-related cancers	E7 TCR cells Aldesleukin Fludarabine Cyclophosphamide	PHASE 1 PHASE 2	180	NCT02858310	RECRUITING	2017/01/27 −2026/01/01	https://clinicaltrials.gov/study/NCT02858310

### EBV-related NPC

Over 90% of undifferentiated NPC cases are associated with EBV infection.^357,849–851^ There is currently no curative treatment for NPC.^852^ For stage I NPC, definitive radiotherapy alone is often effective.^853^ In stage II NPC, there is debate over adding concurrent chemotherapy to radiotherapy, while T2N1 NPC often requires radiotherapy combined with cisplatin-based chemotherapy due to a higher risk of distant metastasis.^854^ For locally advanced (stage III-IVa) NPC, concurrent platinum-based chemoradiotherapy is required.^855^ Depending on the stage and individual patient circumstances, chemotherapy intensity may be further increased by adding induction or adjuvant chemotherapy to concurrent chemoradiotherapy.^762^ Challenges, such as drug resistance and adverse reactions limit chemotherapy’s effectiveness in NPC treatment.^856–858^ EBV infection is the primary cause of NPC, leading to the malignant proliferation of nasopharyngeal mucosal cells and eventually resulting in malignancy.^859^ Due to the pervasive nature of EBV and its transmission challenges, vaccination is considered the most effective preventive measure against EBV infection and the most cost-effective method for treating EBV-related diseases.^95,860^ Advances in understanding NPC’s molecular mechanisms have highlighted the potential of preventive and therapeutic vaccines, along with other strategies like tumor cell signaling targeting, immune checkpoint blockade, and cellular immunotherapy.^861,862^

#### Virus vaccines

Despite numerous attempts to develop an EBV vaccine, none have been approved to date. Preventive peptide vaccines targeting EBV, such as gp350, EBV gH/gL and gB, have been discussed in EBVaGC. This section will focus on therapeutic vaccines for NPC. Virus-like particles (VLPs) represent promising vaccine candidates. VLP-based vaccines for HBV and HPV have been widely applied in clinical practice, suggesting that a similar approach might be effective for an EBV vaccine.^863–865^ The first complete EBV VLP was developed by knocking out EBV’s terminal oncogenes and repeat sequences, such as LMP1 and BZLF1. These EBV VLPs elicited specific responses in mice.^95^ LMP1, LMP2, EBNA1, and EBNA3, either alone or in combination, could serve as target antigens for an anti-EBV vaccine due to their crucial roles in EBV infection and the subsequent malignant transformation of infected cells.^36^ Clinical trial results have shown that immunization with EBV peptide-pulsed DC cells can induce a reduction in serum EBV DNA levels and lead to tumor regression of EBV-related NPC.^866,867^ Additionally, EBV-related NPC aberrantly expresses CD137, which is induced by LMP.^868^ CD137L-DCs exhibit superior T-cell stimulation capabilities in vitro compared to conventional monocyte-derived DCs, indicating potential application in NPC.^869^ Besides DC vaccines, recombinant viral vector vaccines, such as those based on modified vaccinia Ankara (MVA), have also shown promise in NPC.^870,871^ One study demonstrated that MVA fusion proteins could effectively reactivate CD4^+^ memory T-cell responses in vitro, with MVA fusion proteins containing the C-terminal of EBNA1 and full-length LMP2 (MVA-EL).^872^ Two clinical trials involving the MVA-EL vaccine showed enhanced CD4^+^ and CD8^+^ T-cell responses to antigens in NPC patients.^873^ Additionally, the results of two clinical trials related to this vaccine, NCT01800071 and NCT01094405, remain undisclosed. We anticipate the release of the relevant findings (Table 6).

**Table 6:** The completed and recruiting clinical trials of EBV-associated NPC

Conditions	Interventions	Phases	Patients (N)	NCT number	Status	Study period	URL
EBV^+^ NPC	DNR.NPC-specific T cells	PHASE 1	14	NCT02065362	ACTIVE_NOT_RECRUITING	2015/02/01 −2033/02/01	https://clinicaltrials.gov/study/NCT02065362
Locally advanced NPC with detectable EBV DNA	Camrelizumab Nimotuzumab	PHASE 3	459	NCT05772208	RECRUITING	2022/01/01 −2028/01/01	https://clinicaltrials.gov/study/NCT05772208
Locally advanced NPC with detectable EBV DNA	Toripalimab	PHASE 2	198	NCT05628922	RECRUITING	2022/07/02 −2027/07/01	https://clinicaltrials.gov/study/NCT05628922
EBV^+^ Lymphoma EBV^+^ Malignancies	MABEL CTLs Cyclophosphamide Fludarabine	PHASE 1	42	NCT02287311	RECRUITING	2015/02/01 −2029/03/01	https://clinicaltrials.gov/study/NCT02287311
EBV^+^ NPC	EBV CAR-T-cell EBV TCR-T-cell	EARLY_PHASE 1	24	NCT05587543	RECRUITING	2022/12/28 −2030/10/01	https://clinicaltrials.gov/study/NCT05587543
EpCAM^+^ patients with Advanced Solid Tumors	EpCAM CAR-T cells	PHASE 1	30	NCT02915445	ACTIVE_NOT_RECRUITING	2016/07/01 −2025/07/01	https://clinicaltrials.gov/study/NCT02915445
R/R EBV^+^ NPC	EBV CAR-T/TCR-Tcell	EARLY_PHASE 1	24	NCT05587543	RECRUITING	2022/12/28 −2030/10/01	https://clinicaltrials.gov/study/NCT05587543

#### Targeting tumor cell signaling

Currently, there is no specific antiviral treatment for EBV-related NPC. After anti-tumor treatment, EBV levels can drop to normal.^857^ Molecular targeted therapy aims to inhibit tumor cell growth by specifically binding antibodies or ligands to target molecules on tumor cells, thereby blocking downstream signaling pathways crucial for tumor progression.^854,874^ This approach is primarily used for locally recurrent or metastatic NPC (RM-NPC) and includes therapies, such as EGFR monoclonal antibodies and anti-angiogenic agents.^875,876^

In most studies, EBNA1 and LMP1 have been identified as key targets for expanding EBV-specific CTLs (EBV-CTL).^877^ A clinical trial using EBV-CTL to treat NPC patients showed that EBV DNA levels in the plasma of all participants decreased to undetectable levels.^878^ Additionally, EBV-CTL treatment for NPC can stimulate LMP2-specific immune responses, which are beneficial in managing disease progression in stage IV NPC that is resistant to conventional therapies.^879,880^ Chia et al. conducted a phase II clinical trial revealing that the combination of chemotherapy and EBV-CTLs achieved a 71.4% effectiveness rate, with five patients not requiring further chemotherapy within 34 months of starting CTL treatment. This trial achieved the best outcomes among similar studies in advanced NPC patients, suggesting that chemotherapy combined with EBV-CTL is a highly promising treatment method.^881^

#### Immunotherapy

Immunotherapy has had a significant impact on the treatment of RM-NPC, particularly in cases induced by EBV. In EBV-induced NPC, high levels of PD-L1 and extensive lymphocyte infiltration make PD-1 blockade immunotherapy a potentially beneficial approach.^882^ Preclinical studies have shown that EBV proteins, such as LMP1 and EBNA1/2 can regulate PD-L1 levels, thus influencing the extent of immune evasion.^883^ ICIs have garnered considerable attention in the treatment of NPC. These inhibitors can disrupt immune defenses and reinvigorate the body’s natural anti-tumor immune response. The primary targets for ICIs include PD-1/PD-L1 and cytotoxic T-lymphocyte-associated protein 4 (CTLA-4).

Clinical trials targeting RM-NPC patients with anti-PD-1 therapy have reported ORRs ranging from 20.5% to 34.1%.^884–886^ To date, several ICIs have received approval from the FDA. Pembrolizumab has shown good anti-tumor activity and safety in previously treated RM-NPC patients.^887–889^ In one case report, an NPC patient with high PD-L1 expression achieved complete tumor remission after receiving treatment with the PD-L1 inhibitor nivolumab, with no recurrence after 22 months of treatment. This successful outcome paved the way for further trials involving a larger number of NPC patients.^890^ A phase III clinical trial also demonstrated significantly prolonged PFS in RM-NPC patients treated with chemotherapy combined with camrelizumab.^891^ While single ICIs therapy may have limited activity in EBV-related NPC, dual ICIs therapy has shown increased efficacy in solid tumors. In a phase II trial, patients with EBV-positive RM-NPC who had previously failed chemotherapy were treated with a combination of nivolumab and ipilimumab, resulting in a best overall response (BOR) rate of 38%, a median PFS of 5.3 months, and a median OS of 19.5 months, with good tolerance. Biomarker analysis indicated that these results were independent of PD-L1 expression or tumor mutational burden. Although the BOR did not reach the predetermined estimate, patients with low plasma EBV-DNA titers (<7800 IU/ml) tended to have better responses and PFS.^892^ Recently, two registered phase I trials found that advanced nasopharyngeal carcinoma patients treated with anti-PD-1 had better survival and treatment responses if they had a rapid decrease in EBV DNA and high levels of certain EBV genes in their blood and tumors.^893^

#### Cell therapy

Currently, CAR-T cell therapy has not been approved for the treatment of solid tumors. Nevertheless, eight registered clinical trials are investigating its efficacy for NPC (Table 5). A phase I trial demonstrated the feasibility and tolerability of epithelial cell adhesion molecule (EpCAM)-CAR-T therapy in patients with NPC or breast cancer.^894^ In vitro studies have shown that LMP1-specific CAR-T cells killed 70% of NPC cells overexpressing LMP1. Although the effectiveness of LMP1-specific CAR-T cells against NPC cells with lower LMP1 expression remains uncertain, these findings are promising.^895^ Clinical trials of CAR-T cells targeting LMP1 for EBV-related malignancies are ongoing (NCT02980315).

TCR-T cell therapy has quietly entered the field of cancer treatment and has shown unprecedented potential for solid tumors, due to its ability to target multiple tumor antigens. Research into TCR-T cell therapy for NPC is expanding, with the LMP2 antigen emerging as a promising target. LMP2-specific TCR-T cells have been shown to inhibit tumor progression in NPC cell lines expressing LMP2/LMP1 in mouse models.^896^ Currently, four clinical trials are evaluating TCR-T therapy for NPC (Table 6). The most recent trial, initiated in October 2022, aims to compare the efficacy of CAR-T and TCR-T cells in treating NPC (NCT05587543). TCR-T therapy represents an exciting and rapidly advancing field.

### KSHV-related KS

KS is a malignancy strongly associated with KSHV infection. It is frequently observed in patients with HIV/AIDS and those undergoing chronic immunosuppression.^897,898^ Currently, there is no definitive cure for KS. Chemotherapy is commonly employed for advanced cases, with most drugs demonstrating some level of efficacy. However, treatment options are limited for patients who do not respond to chemotherapy, particularly those who develop resistance to multiple chemotherapeutic agents.^899^ Given that KSHV infection is the primary cause of KS, preventing and managing KSHV infection is crucial,^541,900^ and exploring viral vaccines presents a promising treatment option.^533^ For HIV-positive patients, ART should be initiated alongside anti-tumor therapy.^41^ Systemic treatments for KS primarily include immunomodulation, anti-inflammatory therapies, and targeting VEGF receptors.^901^ Immunotherapeutic options mainly include IFN, PD-1 inhibitors, pomalidomide, among others.^902^

#### Virus vaccines and antiviral treatment

KSHV vaccines are developed to target specific KSHV viral proteins (like gB, gH, gL, K8.1, ORF27, ORF28, ORF68) and have been shown to alleviate disease in KS patients.^903^ Additionally, VGC, an anti-KSHV drug, modulates CD4^+^ T cell subsets and the TNF/TNFR axis, enhancing the regulation of inflammatory states and serving as an effective treatment for AIDS-related KS.^904,905^ Primaquine (PQ) also shows positive effects in treating KS.^906^

For AIDS-related KS patients, combined ART also exhibits anti-cancer effects.^41,105,907–909^ Regardless of CD4^+^ T cell levels, it is essential to initiate anti-HIV therapy immediately, after excluding conditions, such as tuberculous and cryptococcal meningitis.^910^ Lifelong treatment is required, and efforts should be made to avoid interruptions due to anti-tumor treatments.^911^ Paclitaxel combined with ART has been shown to improve OS in AIDS-related KS patients.^912^ Additionally, early administration of etoposide alongside ART can reduce mortality in AIDS-related KS patient.^913^

#### Targeting tumor cell signaling

Inhibitors targeting the signaling pathways of KS tumor cells include cytokine Inhibitors, VEGF Inhibitors and proteasome Inhibitors. Tacrolimus, which inhibits IL-2 release and suppresses T lymphocyte activity, has shown a 100% ORR in the treatment of superficial KS with topical application.^914^ VEGF, a key factor in KS endothelial cell proliferation, makes VEGFR inhibitors ideal targets for KS treatment.^531,915,916^ Liposomal doxorubicin combined with the VEGF inhibitor bevacizumab has shown efficacy in treating advanced KS.^917^ Proteasome inhibitors have direct anti-tumor activity, induces lytic activation of KSHV, and inhibits HIV infectivity, thereby improving control of KS and HIV. Bortezomib has demonstrated good tolerability and effectiveness in treating AIDS-related KS.^918^

#### Immunotherapy

Immune dysfunction has been closely associated with the development of KS.^908^ Given that immunogenic viral neoantigens can be harnessed, immune checkpoint inhibitors (ICIs) have emerged as a primary treatment modality for virus-related tumors, including KS. Increased PD-L1 expression in KS-infected monocytes and its association with an inflammatory environment suggest that lytic virus replication may trigger PD-1/PD-L1 activation in KS.^919^ Pembrolizumab have achieved a 71% ORR in KS with promising efficacy and acceptable safety.^920^ Nivolumab and Ipilimumab has demonstrated an 87% ORR in KS, though 22% experienced grade 3-4 adverse events.^921^ Despite the potential of ICIs, the rarity of KS and the absence of randomized clinical trials mean that ICIs treatment for KS still lacks robust clinical evidence. Another challenge is the lack of evidence regarding the optimal systemic treatment sequence for KS. For example, interferon treatment has been shown to induce PD-1 expression in certain malignancies (e.g., ovarian cancer and melanoma).^922,923^ Before using ICIs in KS patients, the therapeutic benefits of drugs like interferon should be considered. Overall, ICIs are an effective treatment option for KS patients. However, further research is needed to determine the efficacy of ICIs and their optimal treatment sequence to maximize therapeutic outcomes. Ongoing clinical trials, such as NCT03469804, NCT02595866, NCT03316274, and NCT03367754, aim to address these uncertainties and refine ICIs treatment strategies for KS (Table 7).

**Table 7:** The completed and recruiting clinical trials of virus-related KS

Conditions	Interventions	Phases	Patients (N)	NCT number	Status	Study period	URL
KS KICS MCD	Pacritinib	PHASE2	65	NCT06052618	NOT_YET_RECRUITING	2024/08/14 −2034/01/01	https://clinicaltrials.gov/study/NCT06052618
HIV-related KS HIV-negative KS	Abemaciclib	PHASE1|PHASE2	43	NCT04941274	RECRUITING	2021/09/29 −2028/06/01	https://clinicaltrials.gov/study/NCT04941274
HIV-related KS	Pomalidomide	PHASE2	26	NCT03601806	ACTIVE_NOT_RECRUITING	2021/04/26 −2026/01/02	https://clinicaltrials.gov/study/NCT03601806
Refractory HIV related KS	Dostarlimab cART	PHASE1	20	NCT05646082	RECRUITING	2023/05/26 −2025/09/01	https://clinicaltrials.gov/study/NCT05646082
HIV-related tumors	ARROW strategies One-time education	NA	4100	NCT06004011	RECRUITING	2024/05/01 −2027/07/31	https://clinicaltrials.gov/study/NCT06004011
People living with HIV	Pembrolizumab	PHASE1	60	NCT03367754	RECRUITING	2018/08/06 −2025/11/20	https://clinicaltrials.gov/study/NCT03367754

### Lymphoma

The increased risk of lymphoma is associated with various viral infections, including EBV, HIV and HCV. Among them, EBV is the most extensively studied virus associated with lymphoma, especially in ENKTCL, BL and cHL. ENKTCL is a type of T-cell lymphoma that is closely linked to EBV infection. Almost all patients with ENKTCL test positive for EBV. BL and cHL are additional types of B-cell lymphomas associated with EBV. BL is highly invasive and sensitive to chemotherapy. cHL is the most common subtype of HL, with the majority (75%-80%) of cHL patients achieving good tumor remission following first-line standard chemotherapy.^924^ However, the prognosis for patients with relapsed/refractory lymphoma remains poor.^925^ Currently, new treatment strategies under development, such as EBV-CTL, pathway inhibitors, ICIs, and CAR-T therapies, hold promise for these patients.^926,927^ The two most common subtypes of HIV-related non-Hodgkin lymphoma are HIV-related DLBCL and HIV-related BL.^928^ The immune functional defects in HIV-infected individuals exhibit distinct characteristics when comparing HIV-related lymphoma to non-HIV infections. Therefore, anti-lymphoma therapy must consider these immune deficiencies, making antiviral treatment a key component of the overall strategy.

#### Antiviral treatment

As elaborated in the previous sections regarding EBVaGC and NPC, the vaccines targeting EBV have been thoroughly discussed. In the context of EBV-related lymphomas, routine antiviral therapeutic interventions are not recommended at present. Virus clearance plays a significant role in lymphomas associated with HCV or HIV. In a study conducted in Italy, 250 NHL patients related to HCV infection were included in the research, with DLBCL and Marginal Zone Lymphoma being the most common types. The majority of patients had tested positive for HCV before the diagnosis of NHL, with a median time of 11 years. Antiviral therapy was effective for indolent NHL patients, achieving an overall response rate of 90%, and significantly prolonging the overall survival after a 7-year follow-up.^929^ And a study on patients with HCV-RNA positive diffuse large B-cell lymphoma showed that, compared to those who did not receive antiviral therapy, undergoing antiviral therapy after achieving remission from chemotherapy could improve their 2-year OS and PFS, leading to a better prognosis.^930^ Additionally, after confirming an HIV infection, it is important to start ART immediately, regardless of CD4 + T lymphocyte levels, as long as tuberculous and cryptococcal meningitis have been ruled out.^910^ According to the AMC-034 study, patients with HIV-related lymphoma who received combined ART experienced accelerated immune recovery while undergoing the EPOCH regimen.^931^

#### Targeting tumor cell signaling

Potential inhibitors of signaling pathways for EBV-related lymphomas include CD30 inhibitors, HDAC inhibitors, XPO1 inhibitors, and the NF-κB signaling pathway, among others. Around 70% of patients with NKTCL show CD30 overexpression.^932^ Brentuximabvedotin (BV) is the first antibody-drug conjugate that targets CD30. It has demonstrated sustained clinical activity and manageable toxicity in patients with relapsed/refractory CD30^+^ and EBV^+^ lymphomas.^933,934^ In patients with R/R ENKTCL, the potent HDAC inhibitor panobinostat, when combined with bortezomib, can provide clinical benefits and reduce EBV PCR titers.^935^ However, HDAC inhibition can trigger EBV reactivation; romidepsin (an HDAC inhibitor) has been reported to cause EBV reactivation.^936,937^ Therefore, the risk of EBV reactivation must be carefully considered when using HDAC inhibitors. While evidence for signaling pathway inhibitors is currently limited, using them in combination with ICIs or other therapies may help strengthen patient outcomes.

Additionally, adding Rituximab to the CODOX-M/IVAC regimen for HIV-positive BL can enhance survival rates without increasing treatment-related toxicity.^938–940^ Based on the high frequency of TP53 mutations in patients with HIV-related DLBCL, researchers are currently exploring the combination of R-EPOCH with Selinexor. A prospective, single-arm, open-label clinical study in China assessed the efficacy and safety of the XR-EPOCH regimen, which combines Selinexor with R-EPOCH, in newly diagnosed patients with HIV-DLBCL. As of May 2024, 10 patients had been enrolled in the study, and all demonstrated sustained treatment responses with manageable safety profiles.^941^

#### Immunotherapy

Approximately 40% of tumor cells in lymphoma patients express LMP1 and LMP2 during latent phase II, making them ideal targets for immunotherapy.^942,943^ EBV-targeted cell therapies include autologous EBV-specific T cell therapy ballaleucel-T, adoptive transfer of EBV LMP1/2-specific CTLs, and autologous LMP-specific CTLs. Among these therapies, autologous EBV-specific T cell therapy ballaleucel-T can induce remission in patients with R/R ENKTCL. The adoptive transfer of EBV LMP1-specific CTLs has been confirmed as a treatment option for EBV-positive cHL.^944^ Furthermore, the adoptive transfer of EBV LMP1/2-specific CTLs has demonstrated efficacy in maintaining sustained clinical responses in ENKTCL following remission.^945^ Additionally, autologous LMP-specific CTLs can induce sustained remission in patients with relapsed or refractory EBV-related lymphoma without obvious toxicity.^946^ However, the clinical application of these therapies may be limited by long manufacturing cycles and high manufacturing failure rates, making them inaccessible to most patients. Prockop et al. utilized an off-the-shelf donor-derived EBV-CTL bank, which can produce durable CR without noticeable toxicity in EBV-related lymphoma patients resistant to rituximab after transplantation.^947^ One of the most vulnerable groups affected by EBV is hematopoietic stem cell transplant (HSCT) recipients. Primary or reactivated EBV in immunocompromised transplant patients may be associated with post-transplant lymphoproliferative disorders (PTLD).^948^ Once a diagnosis of possible or confirmed EBV-PTLD is made, rituximab and reduction of immunosuppression are recommended as first-line treatments.^949^ Second-line options include adoptive cell therapy (like EBV-CTL) and chemotherapy with or without rituximab.^950^ Tabelecleucel is the first allogeneic, off-the-shelf EBV-specific T-cell immunotherapy approved for the treatment of relapsed or refractory EBV-positive post-transplant lymphoproliferative disorders.^951,952^ An ongoing global, multicenter, open-label phase 3 trial (NCT03394365) and expanded access study (NCT02822495) have demonstrated clinical benefits for patients with relapsed or refractory EBV-positive post-transplant lymphoproliferative disorders who have no other approved treatment options.^951,952^ EBV-targeted cell therapy is a favorable treatment strategy for EBV-related lymphoma patients. The availability of an off-the-shelf donor-derived EBV-CTL bank could provide an attractive and innovative treatment option that reduces the risk of graft-versus-host disease.^947,953^

Many ENKTCL patients express PD-L1, with estimates ranging from 39% to 100%.^954^ This suggests that the PD-1/PD-L1 axis is a promising target for immunotherapy.^616,955^ Research indicates that high serum PD-L1 levels correlate with a poor prognosis in ENKTCL patients.^956^ Several monoclonal antibodies targeting PD-1 and PD-L1, including the dual-targeting antibody IBI318, have demonstrated improved clinical outcomes in patients with R/R ENKTCL.^957–960^ Numerous ongoing clinical trials are investigating PD-1/PD-L1 inhibitors in combination with other therapies (Table 8). Furthermore, research on HIV-related DLBCL is currently scarce, with ongoing clinical trials for gene therapy, BTK inhibitors and CAR-T cell therapy (Table 8).

**Table 8:** The completed and recruiting clinical trials of lymphoma, especially viral-related lymphoma

Conditions	Interventions	Phases	Patients (N)	NCT number	Status	Study period	URL
Extranodal NK-T-cell lymphoma	mid-term PET and EBV DNA-directed therapy	PHASE 2	89	NCT06069830	RECRUITING	2023/12/6 −2026/10/01	https://clinicaltrials.gov/study/NCT06069830
EBV^+^ lymphoma	C7R-EBV T cells	PHASE 1	44	NCT04664179	RECRUITING	2022/10/31 −2039/03/30	https://clinicaltrials.gov/study/NCT04664179
EBV^+^ cancer	EBViNT Cell	PHASE 1 PHASE 2a	72	NCT03789617	RECRUITING	2018/12/14 −2024/12/01	https://clinicaltrials.gov/study/NCT03789617
EBV^+^ lymphoma EBV^+^ malignancies	MABEL CTLs Cyclophosphamide Fludarabine	PHASE 1	42	NCT02287311	RECRUITING	2015/02/01 −2029/03/01	https://clinicaltrials.gov/study/NCT02287311
EBV^+^ R/R lymphoma	Nanatinostat Valganciclovir	PHASE 2	140	NCT05011058	RECRUITING	2021/05/28 −2026/12/01	https://clinicaltrials.gov/study/NCT05011058?cond=NCT05011058&rank=1
R/R EBV^+^ T-cell lymphoma	hNeo-T Cyclophosphamide Fludarabine	EARLY PHASE 1	6	NCT06224049	RECRUITING	2023/12/01 −2026/06/30	https://clinicaltrials.gov/study/NCT06224049?cond=NCT06224049&rank=1
Early-stage NK/T-cell lymphoma	Anti-PD-1 antibody Peg-Asparaginase Chidamide	PHASE 2	35	NCT04414969	RECRUITING	2020/06/26 −2025/07/01	https://clinicaltrials.gov/search?cond=NCT04414969
Extranodal natural killer T cell lymphoma	Chidamide Anti-PD1 antibody Pegaspargase DDGP	PHASE 3	142	NCT06255795	NOT YET RECRUITING	2024/02/15 −2028/12/31	https://clinicaltrials.gov/search?cond=NCT06255795
Natural killer/T-cell lymphoma, nasal and nasal-Type	Linperlisib Camrelizumab Pegaspargase	PHASE 1 PHASE 2	43	NCT06376721	RECRUITING	2024/04/14 −2027/10/31	https://clinicaltrials.gov/search?cond=NCT06376721
EBV^+^ Lymphoma disorders EBV^+^ NHL	Nivolumab	PHASE 2	40	NCT03258567	RECRUITING	2018/04/26 −2031/06/01	https://clinicaltrials.gov/study/NCT03258567
R/R CD30^+^ lymphoma	CD30 CAR-EBVST cells	PHASE 1	18	NCT04288726	RECRUITING	2020/09/16 −2037/06/01	https://clinicaltrials.gov/study/NCT04288726
CD30^+^ lymphoma	C7R. CD30 CAR-EBVST cells	PHASE 1	90	NCT06176690	NOT_YET_RECRUITING	2024/06/01 −2043/06/01	https://clinicaltrials.gov/study/NCT06176690
ARL	Busulfan Lentivirus vector rHIV7-shI-TAR-CCR5RZ-transduced hematopoietic progenitor cells	PHASE 1	3	NCT01961063	ACTIVE_NOT_RECRUITING	2015/12/31 −2024/12/30	https://clinicaltrials.gov/study/NCT01961063
Stem cell transplantation ARL	ASCT Carmustine	PHASE1 |PHASE 2	11	NCT02797470	ACTIVE_NOT_RECRUITING	2016/06/23 −2025/06/30	https://clinicaltrials.gov/study/NCT02797470
B-cell NHL HIV	CAR-T Cyclophosphamide Fludarabine	PHASE 1	20	NCT05077527	RECRUITING	2024/050/3 −2027/01/31	https://clinicaltrials.gov/study/NCT05077527
HIV^+^ DLBCL	EPOCH R- EPOCH R-da- EPOCH	PHASE 1	54	NCT03220022	RECRUITING	2018/03/16 −2025/06/20	https://clinicaltrials.gov/study/NCT03220022
AIDS-related NHL	R-EPOCH rHIV7-shI-TAR-CCR5RZ	PHASE 1	10	NCT02337985	ACTIVE_NOT_RECRUITING	2015/11/20 −2024/12/31	https://clinicaltrials.gov/study/NCT02337985

#### CAR-T therapy

Recent preclinical studies indicate that four CAR-T cell lines (CD38-CAR, LMP1-CAR, and their tandem variant CAR 2) exhibited significant cytotoxicity against NKTCL cells in both in vitro and in vivo settings.^961^ CD38-CAR-T cells exhibited a remarkable inhibitory effect on NKTCL in vitro and mouse xenograft models.^962^ Currently, a phase I trial is underway to evaluate the safety and feasibility of using fully human anti-CD30 CAR cells for treating advanced CD30-expressing lymphomas. CD30 CAR-T cells have demonstrated antitumor efficacy and low toxicity in R/R HL. However, the use of off-the-shelf allogeneic T-cell therapies is limited due to the risk of graft-versus-host disease.^963^ To address this issue, promising results from a study presented at the 17th International Conference on Malignant Lymphoma (ICML) showed that modifying EBV-specific T cells (EBVST) with a CD30-CAR (CD30CAR EBVST) could provide a safe and effective treatment for R/R HL.^964^ A single-center study conducted in China revealed that CD19/CD22 CAR-T therapy is effective and has manageable toxicity in adults with R/R BL.^965^ For children R/R BL, patients who have reached CR after infusion of CD19 CAR-T continue to infuse the CD22 CAR-T, which can produce lasting effects. And the patient with the suffering of the central nervous system has benefited obviously.^966^ Although clinical trial data on CAR-T therapy for EBV-related lymphomas are limited, the outlook remains optimistic.

Currently, CAR-T therapy for HIV-related lymphomas (HAL) is supported only by case reports, as key trials have not included HIV-positive patients. In six reported cases, three patients achieved CR, one achieved PR, and the treatment was considered safe.^967^ As research continues, increased data will enable us to better evaluate the long-term effects and safety of CAR-T therapy for virus-associated lymphoma.

### Adult T-cell leukemia/lymphoma (ATLL)

After the Human T-cell leukemia virus type 1 (HTLV-1) invades the human body, the incubation period can last for decades, with about 3% of HTLV-1 infected individuals eventually developing ATLL.^968^ Currently, there is no curative treatment for HTLV-1 and ATLL. Many ATLL patients experience infections, requiring aggressive infection control and enhancement of organ function. When the disease progresses, prompt and aggressive treatment measures, such as chemotherapy, targeted therapy, and biological therapy are necessary.^969^ Although some patients experience alleviation of clinical symptoms during the treatment process, these therapeutic methods still have certain limitations. For instance, patients who undergo chemotherapy suffer from a severe weakening of their immunity, while stem cell transplantation is prone to lead to a high recurrence of the disease.^635^ As HTLV-1 infection is the primary cause of ATLL, it is crucial to prioritize the prevention and control of HTLV-1 infection. Currently, there are no available antiviral drugs or vaccines for HTLV-1, making research into antiviral drugs and vaccines a potential therapeutic direction.^970^

#### Virus vaccines and antiviral treatment

Patients with ATLL typically opt for a combination therapy of Interferon-alpha (IFN-α) and Zidovudine (AZT) as the first-line treatment plan.^971^ Compared to monotherapy with first-line chemotherapy or chemotherapy followed by antiviral maintenance therapy, first-line antiviral treatment primarily benefits patients with acute, chronic, and smoldering types of ATLL.^972^ It has been reported that the five-year survival rate for patients with chronic and smoldering ATLL who received combined IFN-α and AZT therapy is 100%, significantly improving the long-term prognosis of patients.^973^ Although patients treated with the combination of IFN-α and AZT show a clear survival advantage, and there is theoretical research supporting the synergistic antiviral effect of IFN-α and AZT, the potential threat of cytotoxicity cannot be ruled out due to the often ineffective low doses of IFN-α and AZT. As a long-term management strategy, whether this approach can lead to a complete cure for patients remains to be further studied, and it is necessary to conduct clinical trials to evaluate new targeted therapies.^974^ Epigenetic abnormalities contribute to the pathogenesis of ATLL, and a phase 2 clinical study evaluated the efficacy and safety of Valemetostat, an EZH2 inhibitor, in patients with R/R ATLL. The study found an ORR of 45.8%, and the median DOR has not yet been reached. These results suggest that Valemetostat demonstrates promising efficacy and tolerability in patients with R/R ATLL.^975^

Therapeutic vaccines represent a potential therapeutic strategy against HTLV-1 infection, aiming to control ATLL by activating the body’s immune response. A novel immunogenic chimeric peptide vaccine that includes epitopes from Tax, gp21, gp46, and gag (p19) encompasses multiple antigenic epitopes related to HTLV-1. The vaccine is immunized with MPLA and ISCOMATRIX adjuvants, and experimental results indicate that this chimeric peptide vaccine can induce systemic and mucosal immune responses, especially when MPLA and IMX adjuvants are used in combination.^976^ This suggests that the vaccine has potential in eliciting cellular and mucosal immune responses, offering new insights for combating HTLV-1 infection. Additionally, a multi-epitope vaccine delivered via PLGA nanoparticles can significantly enhance systemic and mucosal immune responses.^977^ The application of multi-epitope vaccines and immunostimulants can improve immune responses, providing a new avenue for the development of therapeutic vaccines for HTLV-1.

#### Targeting tumor cell signaling

In the process of developing anti-HTLV-1 treatment methods, the greatest obstacle is the difficulty in controlling the viral permanence and the reactivated provirus. However, the emergence of a new treatment method, gene editing technology, has brought hope for treating this difficult problem.^978,979^ As early as 2013, zinc finger nucleases (ZFNs) specifically recognizing the HTLV-1 LTR were confirmed to be able to disrupt the LTR promoter function of HTLV-1 and inhibit the proliferation of HTLV-1-positive cell lines.^980^ Subsequently, the gDNA developed based on the CRISPR/Cas9 technology and targeting the HTLV-1 oncogenes HBZ and Tax was also confirmed to be able to inhibit the proliferation of ATLL cells to varying degrees.^981^ The latest research also reported that using substrate-linked directed evolution (SLiDE) to engineer Cre-derived site-specific recombinases, named RecHTLV recombinase, and recombinase can accurately target and effectively excise the HTLV-1 provirus in HTLV-1-infected cells, potentially preventing the development of HTLV-1-associated diseases.^982^ In conclusion, gene editing technology has broad application prospects in the treatment of anti-HTLV-1.

## Conclusions and perspectives

Since the identification of the first human oncogenic virus, EBV, in the lymphocytes of Burkitt lymphoma patients in 1964, the number of recognized oncogenic viruses has increased with advancements in high-throughput technologies, deepening our understanding of these viruses. IARC has classified seven viruses as Group 1 carcinogens, including four DNA viruses and three RNA viruses. The distribution of virus-induced tumors varies globally, indicating that multiple factors influence the oncogenic risks and incidence rates of these viruses in different regions, necessitating further research to enhance our understanding.

The role of oncogenic viruses in cancer development is complex and multifaceted. On the molecular level, this review discusses how these viruses infect target cells and induce carcinogenesis. Different oncoviruses vary in genomic structure, ways of invasion, replication, and assembly; and these differences directly influence their subsequent carcinogenic mechanisms. First, various oncoviruses enter cells by binding to specific receptors on the surface of host cells through different structural proteins. DNA viruses (HBV, HPV, EBV, KSHV, MCV) can directly integrate into the genome of target cells, while retroviruses (HIV, HTLV-1) need to be reverse-transcribed into DNA first and then integrate into the host genome. After the oncovirus integrates into the host genome, it may activate oncogenes or inactivate tumor-suppressor genes, triggering carcinogenesis. Although HCV does not integrate, the chronic inflammation caused by persistent infection can increase the risk of cell mutation and thus lead to cancer. Meanwhile, the cell tropism of the virus determines the type of cancer it causes. For instance, EBV mainly infects B lymphocytes and epithelial cells and is associated with NPC and B-cell lymphoma; HPV infects basal epithelial cells, and persistent infection with high-risk types increases the risk of CC.

Oncogenic viruses affect host cells in various ways, including directly inducing genomic instability, regulating the expression of oncogenes and tumor suppressor genes, disrupting cell cycles, causing immune suppression, triggering metabolic reprogramming, and promoting chronic inflammation. These mechanisms provide critical insights into how oncogenic viruses lead to cancer. Although these viruses share many direct or indirect carcinogenic mechanisms, there are differences between DNA and RNA viruses. DNA viruses tend to cause cellular transformation by encoding viral oncoproteins, such as the HBx protein of HBV and E6/E7 proteins of HPV, which directly alter host cell signaling pathways and cell cycles. In contrast, RNA viruses often promote oncogenesis through chronic infection, leading to sustained inflammation or immune suppression, facilitating mutations and cancer. This tendency is most evident in the case of HIV, which, despite being classified as an oncogenic virus, is widely believed not to cause cancer directly but rather to promote certain cancers indirectly by compromising the immune system. Some viruses have also been found to invade the nervous system, and their presence might disrupt normal neural-regulatory mechanisms, which could influence the microenvironment conducive to cancer initiation or progression. Meanwhile, a dysbiotic microbiome might either enhance or suppress the replication and oncogenic potential of viruses. Some bacteria in the microbiome could produce metabolites that directly interfere with virus-host interactions or indirectly modulate the host’s immune system. These mechanisms not only promote cancer development but also complicate and challenge cancer treatment.

Targeted virus therapy for virus-related tumors offers significant advantages. Virus vaccines can trigger a highly specific immune response against relevant antigens, helping to maintain long-term immune surveillance. Precision gene-targeted therapy modifies viruses to target tumor-causing genes without harming normal cells. ICIs can be combined with chemotherapy to enhance anti-tumor immunity. Meanwhile, the treatment of virus-related cancers encounters numerous clinical obstacles. Oncogenic viruses, such as HPV, exhibit high variability with multiple subtypes and genetic mutations, which not only complicates the determination of stable therapeutic targets but also render existing drugs ineffective when mutations occur. Additionally, the latent infection state, as seen in EBV, poses a significant challenge. During latency, the virus is scarcely detectable by the immune system and resistant to antiviral drugs. Moreover, cancers linked to these viruses often possess intricate immune-escape mechanisms, like HBV-induced HCC downregulating MHC molecules to evade immune surveillance. These treatment challenges are closely associated with the carcinogenic mechanisms in which viruses integrate into the host genome. Thus, the key points of treatment are to block virus integration to impede cancer development and to mitigate virus-induced inflammation to lower the risk of cell mutation.

While significant progress has been made in understanding viral oncogenesis and developing therapeutic strategies, many challenges and unknowns remain. Developing therapeutic vaccines will be a crucial breakthrough in the future, helping to enhance immune protection in virus-infected populations and reduce the incidence of virus-related cancers. In addition to vaccines, antiviral therapies and emerging anticancer treatments are also areas of interest. For example, antiviral therapy for chronic hepatitis has been proven to reduce the risk of liver cancer significantly. Therefore, antiviral therapy should be maintained throughout the course of curative or systemic treatment. In terms of targeted and immunotherapies, although current therapies offer limited survival benefits, further understanding of the immune system may improve treatment outcomes through checkpoint blockade strategies and engineered T-cell immunotherapy. Lastly, strengthening public health education, promoting vaccination, and expanding antiviral treatment access will provide a solid foundation for reducing the incidence of virus-related cancers. In the future, in-depth research into carcinogenic mechanisms, accurate identification of key targets, and the development of novel therapies capable of breaking virus latency, overcoming immune escape, and blocking virus integration will be essential for surmounting these treatment challenges.
